# Global hospital admissions and in-hospital mortality associated with all-cause and virus-specific acute lower respiratory infections in children and adolescents aged 5–19 years between 1995 and 2019: a systematic review and modelling study

**DOI:** 10.1136/bmjgh-2021-006014

**Published:** 2021-07-14

**Authors:** Xin Wang, You Li, Xin Mei, Erin Bushe, Harry Campbell, Harish Nair

**Affiliations:** 1 Centre for Global Health, Usher Institute, The University of Edinburgh, Edinburgh, Edinburgh, UK; 2 School of Public Health, Nanjing Medical University, Nanjing, Jiangsu, China

**Keywords:** pneumonia, epidemiology, paediatrics, systematic review, respiratory infections

## Abstract

**Introduction:**

The burden of acute lower respiratory infections (ALRI), and common viral ALRI aetiologies among 5–19 years are less well understood. We conducted a systematic review to estimate global burden of all-cause and virus-specific ALRI in 5–19 years.

**Methods:**

We searched eight databases and Google for studies published between 1995 and 2019 and reporting data on burden of all-cause ALRI or ALRI associated with influenza virus, respiratory syncytial virus, human metapneumovirus and human parainfluenza virus. We assessed risk of bias using a modified Newcastle-Ottawa Scale. We developed an analytical framework to report burden by age, country and region when there were sufficient data (all-cause and influenza-associated ALRI hospital admissions). We estimated all-cause ALRI in-hospital deaths and hospital admissions for ALRI associated with respiratory syncytial virus, human metapneumovirus and human parainfluenza virus by region.

**Results:**

Globally, an estimated 5.5 million (UR 4.0–7.8) all-cause ALRI hospital admissions occurred annually between 1995 and 2019 in 5–19 year olds, causing 87 900 (UR 40 300–180 600) in-hospital deaths annually. Influenza virus and respiratory syncytial virus were associated with 1 078 600 (UR 4 56 500–2 650 200) and 231 800 (UR 142 700–3 73 200) ALRI hospital admissions in 5–19 years. Human metapneumovirus and human parainfluenza virus were associated with 105 500 (UR 57 200–181 700) and 124 800 (UR 67 300–228 500) ALRI hospital admissions in 5–14 years. About 55% of all-cause ALRI hospital admissions and 63% of influenza-associated ALRI hospital admissions occurred in those 5–9 years globally. All-cause and influenza-associated ALRI hospital admission rates were highest in upper-middle income countries, Asia-Pacific region and the Latin America and Caribbean region.

**Conclusion:**

Incidence and mortality data for all-cause and virus-specific ALRI in 5–19 year olds are scarce. The lack of data in low-income countries and Eastern Europe and Central Asia, South Asia, and West and Central Africa warrants efforts to improve the development and access to healthcare services, diagnostic capacity, and data reporting.

**Key questions**

**What is already known?**

- Acute lower respiratory infections (ALRI) caused an estimated 55 800 deaths, accounting for 5% of all-cause deaths in children aged 5–19 years globally in 2019.
- No global estimates are available to quantify hospital admissions due to ALRI in this age group, and only the Institute for Health Metrics and Evaluation estimated global influenza-attributable hospital admissions (about 2.0 million).

**What are the new findings?**

- Based on data from 202 studies, we estimated that globally about 5.5 million all-cause ALRI hospital admissions occurred annually between 1995 and 2019 in the 5–19 years age group and 87 900 in-hospital deaths; 19% (1.1/5.5 million) of ALRI hospital admissions were associated with influenza virus and 4% (231 800) for respiratory syncytial virus in the 5–19 years age group; human metapneumovirus (105,500) and human parainfluenza virus (124,800) was associated with 2% of ALRI hospital admissions in the 5–14 years age group separately.
- 55% of ALRI hospital admissions and 63% of the influenza-associated ALRI hospital admissions occurred in children aged 5–9 years.
- Estimates of all-cause ALRI hospital admissions were developed in 39 countries and estimates of influenza-associated ALRI hospital admissions were developed in 26 countries, accounting for 57% and 52% of the global population 5–19 years of age.

**Key questions**

**What do the new findings imply?**

- Incidence and mortality data for all-cause and virus associated ALRI in 5–19 year olds are scarce; the lack of data in low-income countries and Eastern Europe and Central Asia, South Asia, and West and Central Africa warrants efforts to improve the development, access to healthcare services, diagnostic capacity and data reporting.
- The estimates provide evidence to support introduction of ALRI intervention programmes in the 5–19 years age group.

## Introduction

Although the global acute lower respiratory infection (ALRI) mortality burden for 5–19 years reduced by about 43% between 1990 and 2019, it was estimated that ALRI caused around 56 000 deaths in children and adolescents aged 5–19 years globally in 2019, accounting for 5% of global all-cause deaths in this age group.^1^ Several efforts have been made to improve estimation of all-cause and virus-specific ALRI burden for children under 5 years; however, the burden in those aged 5–19 years are less well understood. In the absence of a baseline disease burden estimate, it is difficult to understand the impact of change in virulence of an existing pathogen or emergence of a novel pathogen (eg, SARS-CoV-2) and their impact on all-cause/aetiology-specific ALRI disease burden. No global estimates of all-cause ALRI hospital admissions have been made for 5–19 years, and very few global estimates quantify the burden of common ALRI viral aetiologies for this age group. Estimates have only been made for influenza attributable ALRI burden in one study by Institute for Health Metrics and Evaluation (IHME).^2^ This study estimated that globally in 2017, approximately 2.0 million lower respiratory infection hospital admissions attributable to influenza for 5–19 years.^2^ No regional or country-specific estimates were reported in the paper. These estimates were developed through a complex modelling process. Despite the importance of models in global burden estimation, data allowing for direct estimation of influenza-related hospital admissions, for example, hospital admission rate for 5–19 years, offer an opportunity to reduce model-related uncertainties and to improve the robustness of estimates.

Given the differences in access to health services between countries, we included country-level layer in the analytical framework when data were sufficient. Hospital admission and in-hospital mortality estimates at country and region levels would inform the burden on health services and provide information for intervention impact analyses. In resource-limited settings, hospital care data would inform allocation of healthcare recourses, and warrant efforts to improve access to healthcare and development of healthcare registration systems.

## Methods

### Systematic review and assessment of risk of bias

We conducted a systematic review of burden of all-cause ALRI and of cause-specific ALRI associated with four viruses (ie, ALRI with laboratory-confirmed influenza virus, respiratory syncytial virus, human metapneumovirus, or human parainfluenza virus) in children and adolescents aged 5–19 years (online supplemental appendix pp1-4). The four viruses are commonly associated with ALRI in children under 5 years.^3^ We searched Medline (Ovid), Embase (Ovid), Global Health (Ovid: 1973 onwards), Cumulative Index to Nursing and Allied Health Literature, Web of Science, Global Health Library, three Chinese language databases (CNKI, Wanfang and Chongqing VIP), and Google search (for grey literature). No language or publication restrictions were applied, and three reviewers (XW, XM and EB) screened the titles and abstracts for eligibility, and extracted data independently. Disagreements were resolved by discussion between reviewers.

10.1136/bmjgh-2021-006014.supp1 Supplementary data

We included studies that were published between 1 January 1995, and 31 December 2019, and reported any of the following data for 5–19 years: hospital admission rates of all-cause and cause-specific ALRI; in-hospital case-fatality ratios (hCFRs) of all-cause ALRI; and proportions of hospitalised ALRI cases positive for the four respiratory viruses. Community-based studies with data on incidence rates of all-cause ALRI and hCFRs of virus-associated ALRI were also identified (online supplemental appendix p23; 42–44), but were not included in the quantitative analysis due to the small number of studies.

Studies using hospitalised ALRI (pneumonia and bronchiolitis) or similar definitions (eg, acute respiratory infections or chest X-ray confirmed pneumonia requiring hospital admission) were eligible (online supplemental appendix pp20-22). Studies that reported data on hospital admission rates and hCFRs of all-cause ALRI, and proportion of virus positives were required to report data for at least a 12 consecutive month period. Studies that reported data on hospital admission rates of virus-associated ALRI were required to show data for at least one full virus season if in a temperate region with defined seasons, or at least a 12 consecutive month period. There were no restrictions on the duration of studies reporting hCFRs of virus-associated ALRI. We excluded studies: where the denominator was unavailable or could not be calculated; reporting modelled burden estimates; in which viral infections were diagnosed based on serology alone; which reported only single infections or only coinfections of a virus; or which only including population subgroups with high-risk conditions. For influenza, we additionally excluded studies which reported data during the 2009 pandemic (the 2009–2010 season in temperate regions and the year 2009 in tropical regions) to estimate the burden of influenza during average seasons; and those reporting data on a non-dominant influenza subtype. This study was registered with PROSPERO, CRD42018093336.

We used a modified Newcastle-Ottawa Scale to assess the risk of bias in six domains, including study design, adjustment for healthcare utilisation, patient groups excluded, case definition, and sampling strategy and test methods for studies with virus-specific data (online supplemental appendix p45).^4 5^

### Statistical analysis

#### ALRI hospital admissions

**Figure 1:**
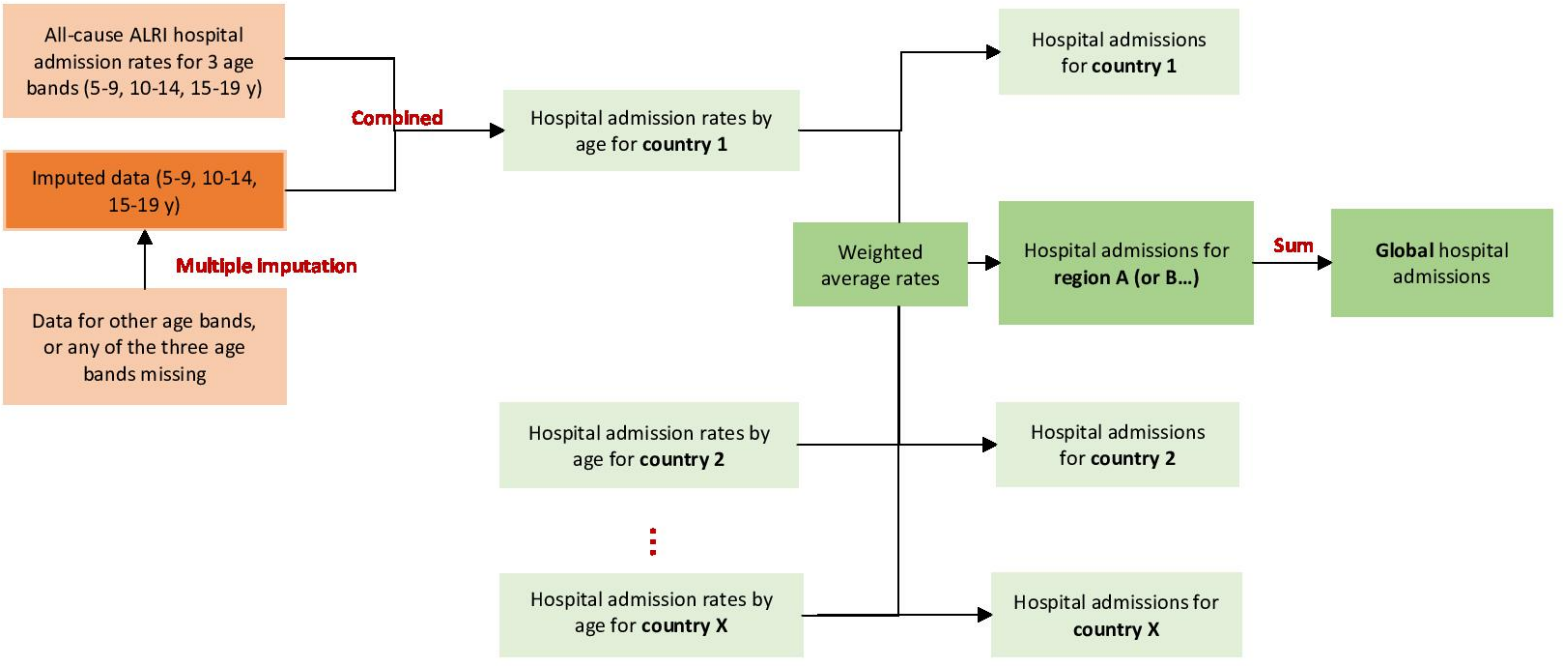
Estimation of global, regional, and national all-cause acute lower respiratory infections (ALRI) hospital admissions in children and adolescents aged 5–19 years. ‘Orange’ boxes show input data, and ‘green’ boxes show the outputs. All analyses were done by three age bands (5–9, 10–14 and 15–19 years). Analyses were done at national level and regional level (World Bank income region and UNICEF region). Global estimates were calculated as sum of estimates by age and World Bank income region. Estimation of influenza-associated ALRI hospital admissions were generally similar to estimation of ALRI hospital admissions, with the hospital admission rates of influenza-associated ALRI as the input. We made one adaptation: prior to meta-analyses, influenza hospital admission rates were adjusted for testing practice in each study where available. y: year.

Figure 1 summarises our approach to estimation of ALRI hospital admissions and influenza virus-associated ALRI hospital admissions. We stratified analyses by three 5-year age groups (5–9 years, 10–14 years and 15–19 years) for which data were most frequently reported. Not all studies reported ALRI hospital admissions rates for the three age groups. We imputed missing data for any of the three age groups for each study using a multiple imputation approach; the input data are rate ratios between the three age groups estimated using a network meta-analysis and United Nations Population Division country population structures by single-year of age (online supplemental appendix pp5-7).^5–7^ We estimated average rate ratios based on studies where age-stratified data were available (nine studies for ALRI and eight studies for influenza-associated ALRI). The rate ratios were then extrapolated to other studies to estimate hospital admission rates by age groups. We assumed the hospital admission rate was similar within each 5 year age bands. Estimates were comparable after the imputation was done (online supplemental appendix p7).

Meta-analyses were conducted by country and by World Bank income regions and UNICEF regions.^8 9^ Due to the insufficient data for South Asia, we combined this region with the region of East Asia and the Pacific (as Asia-Pacific); we combined the region of Eastern and Southern Africa and the region of West and Central Africa (as Sub-Saharan Africa) due to insufficient data in West and Central Africa. First, we pooled hospital admission rates for each country using a generalised linear mixed model (binomial-normal model: within-study binomial distribution and between-study normal distribution) when there were two or more studies.^10^ When there was only one study for a country, the reported hospital admission rates in the study were used to yield country-specific hospital admissions. The weighted average of available country-specific rates and the sum of weighted uncertainties in each region was extrapolated to yield the number of ALRI hospital admissions in the region.^11^ Global results were calculated as the sum of estimates by age group and World Bank income regions. We also reported estimates by UNICEF regions where data were available. The numbers of cases and deaths were rounded to the nearest hundred.

After meta-analyses, the number of all-cause ALRI hospital admissions was estimated using the Monte Carlo Simulation to combine the meta-estimates of ALRI hospital admission rates and UN Population Division census estimates for 2019 as done previously.^5 6^ The median value of 1000 samples simulated from a log–normal distribution was used as the point estimate and the 2∙5th and 97∙5th percentiles as the 95% uncertainty ranges (UR) for disease burden estimates.

#### All-cause ALRI in-hospital mortality

The available hCFR data for all-cause ALRI were insufficient to allow for disaggregation by 5-year age group or country. We pooled hCFR for the overall age group (5–19 years) by World Bank income (as well as UNICEF) regions, and applied the hCFR meta-estimate to the number of ALRI hospital admissions to yield all-cause ALRI in-hospital mortality in each region as previously.^5^

### Influenza-associated ALRI hospital admissions

Estimation of influenza-associated ALRI hospital admissions is generally similar to that for all-cause ALRI hospital admissions except for one adaptation: hospital admission rates of influenza were additionally adjusted for levels of testing per study (where such data were available) before meta-analyses. We did not make this adjustment if this was already accounted for and reported by the study.

### ALRI hospital admissions associated with respiratory syncytial virus, human metapneumovirus and human parainfluenza virus

For the three above-mentioned viruses, data on hospital admission rates were limited; thus, we estimated virus-associated ALRI hospital admissions using a proportion-based approach, by combining the proportion positives of a virus and the number of all-cause ALRI hospital admissions. URs were estimated using the Monte Carlo Simulation. We conducted analyses by World Bank income (and UNICEF) regions where data were available. For respiratory syncytial virus, we estimated hospital admissions for the overall age group 5–19 years; for human metapneumovirus and parainfluenza virus, we estimated the hospital admissions for 5–14 years as data were most commonly reported for this age group.

### Sensitivity analyses

For all-cause ALRI hospital admissions, we excluded potential outlier studies in the sensitivity analysis. For influenza-associated ALRI hospital admissions, we excluded potential outlier studies and studies using the definition of ‘International Classification of Diseases (ICD)-coded laboratory confirmed respiratory hospital admissions’ in sensitivity analyses. Additionally, we estimated influenza-associated ALRI hospital admissions by combining the pooled estimates of proportion positives of influenza and estimates of all-cause ALRI hospital admissions in a sensitivity analysis. This study was conducted and reported in accordance with the Guidelines for Accurate and Transparent Health Estimates Reporting recommendations (online supplemental appendix p59).^12^

### The role of funding source

The funder of the study had no role in study design, data collection, data analysis, data interpretation, or writing of the report. XW and HN had full access to all the data in the study and HN had final responsibility for the decision to submit for publication.

### Patient and public involvement

Patients and the public were not involved in this study.

## Results

**Figure 2:**
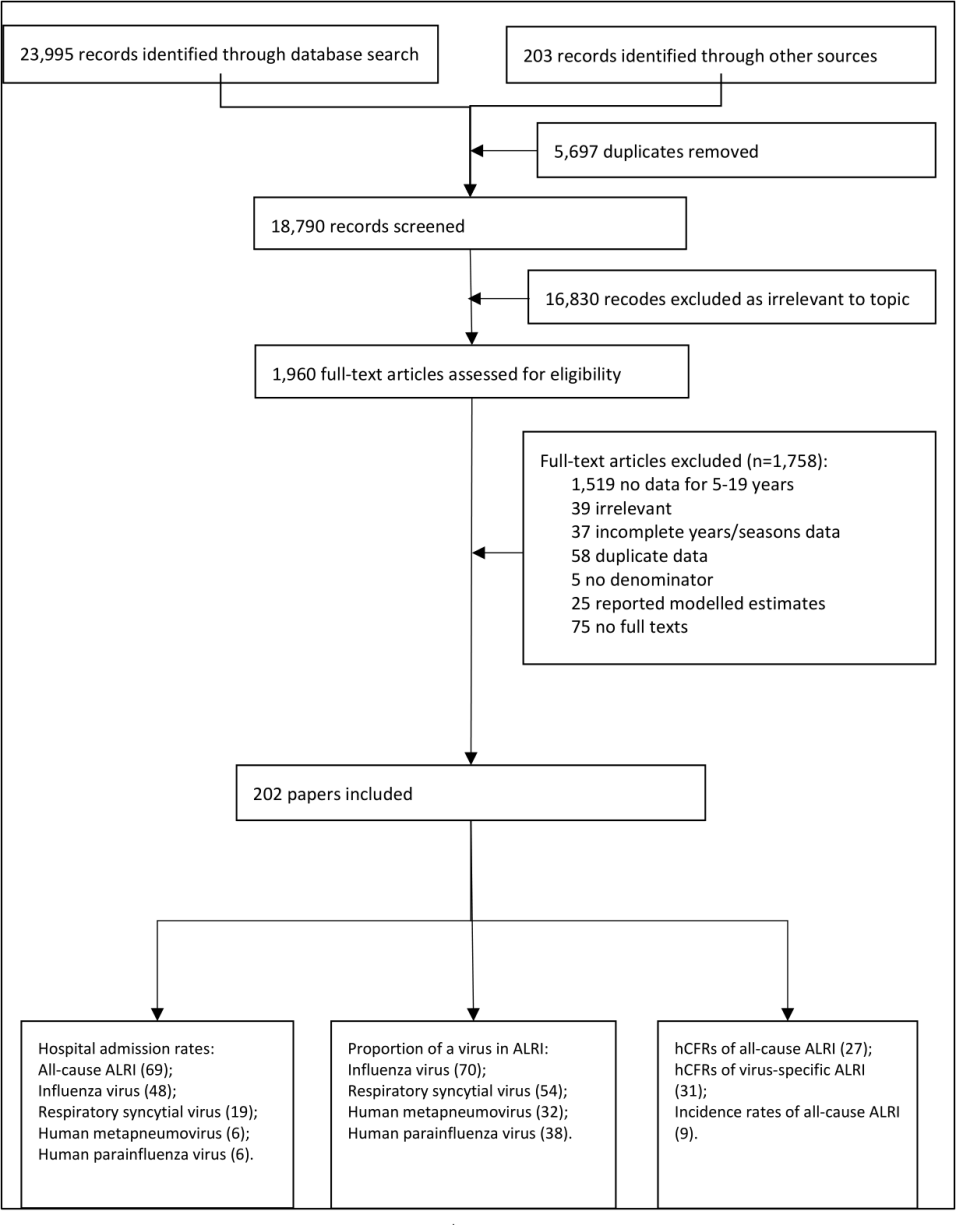
Flow diagram for study selection. ALRI, acute lower respiratory infection. hCFR, in-hospital case-fatality ratio.

We identified 202 studies with relevant data between 1995 and 2019 (figure 2). There were 90 studies with data for all-cause ALRI (ie, incidence rates, hospital admission rates, and hCFRs), 114 studies for influenza virus, 66 studies for respiratory syncytial virus, 37 studies for human metapneumovirus, and 42 studies for human parainfluenza virus. By World Bank income region, six (3%), 38 (19%), 62 (31%) and 96 studies (47%) were from low-income, lower middle-income, from upper middle-income, and from high-income countries, respectively. By UNICEF region, most of the studies were from high-income countries (41%) and East Asia and the Pacific (27%). Figure 2 shows the study selection for the systematic review. Quantitative analyses were not conducted for incidence rates of all-cause ALRI or hCFRs of virus-specific ALRI given the paucity of data. Details of all the included studies are in the online supplemental appendix pp19-43.

There were 69 studies with data on all-cause ALRI hospital admission rates, including 7 studies reporting data for pre-2000 period, 34 studies for 2001–2010, and 28 studies for 2011–2018. Data were from 39 countries, and nationally representative data (as reported in individual studies) were found in 15 countries. Of these studies, 33 studies had a low risk of bias in study design, 64 studies had a low risk in adjustment for healthcare utilisation, and 68 studies had a low risk of bias in patient groups excluded. Thirty-nine studies used definitions similar to that we specified, 26 studies used less specific definitions, and 4 studies used more specific definitions. Across 39 countries, all-cause ALRI hospital admission rate meta-estimates ranged from 0.1 (95% CI 0.1 to 0.1) per 1000 children per year to 42.7 (95% CI 35.1 to 51.8) for 5–9 years, from 0.1 (95% CI 0.1 to 0.1) to 17.4 (95% CI 12.9 to 23.6) for 10–14 years, and from 0.1 (95% CI 0.1 to 0.1) to 18.1 (95% CI 13.1 to 25) for 15–19 years (table 1). By World Bank income regions, the estimated all-cause ALRI hospital admission rate for 5–19 years was highest in upper middle-income countries (3.9 per 1000 persons per year (95% CI 2.6 to 5.6)), followed by lower middle-income countries (2.9 (95% CI 2.1 to 4.1)), low-income countries (1.9 (95% CI 1.4 to 2.6)), and high-income countries (1.4 (95% CI 1.2 to 1.7)). By UNICEF regions, we estimated higher all-cause ALRI hospital admission rates for 5–19 years in Asia-Pacific, and Latin America and Caribbean (3.0–3.6 per 1000 persons per year), followed by Sub-Saharan Africa (2.2 (95% CI 1.9 to 2.6)), high-income countries (1.3 (95% CI 1.2 to 1.6)), and Middle East and North Africa (0.7 (95% CI 0.6 to 1.1)). Estimates were not calculated for Eastern Europe and Central Asia due to the non-availability of data. Globally, we estimated 5.5 million (UR 4.0–7.8) all-cause ALRI hospital admissions in children and adolescents 5–19 years of age annually, and about 55% (3.0/5.5 million), 23% (1.3 million) and 23% (1.3 million) of the hospital admissions occurred among 5–9 years, 10–14 years and 15–19 years, respectively.

**Table 1:** Estimates of acute lower respiratory infections (ALRI) hospital admission rates (per 1000 persons/year) and hospital admissions in children and adolescents aged 5–19 years, by three age groups, country, UNICEF region, and World Bank income region

Country/region		5–9 years	10–14 years	15–19 years	5–19 years
Australia	No. of data points*	3 (3)	3 (3)	3 (3)	
	Hospital admission rate†	2.3 (1.6–3.4)	0.9 (0.7–1.3)	1 (0.7–1.4)	
	Hospital admissions	3800 (2700–5400)	1500 (1000–2100)	1500 (1000–2100)	6800 (4700–9600)
Bolivia	No. of data points*	6 (6)	6 (6)	6 (6)	
	Hospital admission rate	42.7 (35.1–51.8)	17.4 (12.9–23.6)	18.1 (13.1–25)	
	Hospital admissions	50 400 (41 500–60700)	20 300 (15 000–27100)	20 500 (14 900–27900)	91 200 (71 400–1 15 700)
Brazil	No. of data points*	13 (8)	13 (8)	13 (8)	
	Hospital admission rate	3.2 (2.9–3.4)	1.2 (0.9–1.5)	1.3 (1.1–1.4)	
	Hospital admissions	46 200 (42 800–49700)	18 200 (14 300–23000)	20 900 (18 600–23300)	85 300 (75 700–96000)
Cambodia	No. of data points*	3 (3)	3 (3)	3 (3)	
	Hospital admission rate	2.1 (0.3–14.3)	0.9 (0.1–6.1)	0.9 (0.1–6.6)	
	Hospital admissions	3700 (500–23400)	1500 (200–9200)	1300 (200–9100)	6500 (900–41700)
Canada	No. of data points*	30 (24)	30 (24)	30 (24)	
	Hospital admission rate	1 (0.9–1.1)	0.4 (0.4–0.4)	0.4 (0.3–0.4)	
	Hospital admissions	1900 (1700–2100)	800 (700–900)	800 (700–900)	3500 (3100–3900)
China	No. of data points*	10 (10)	10 (10)	10 (10)	
	Hospital admission rate	7.3 (4.8–11.1)	3.1 (2–4.7)	3.2 (2.1–4.9)	
	Hospital admissions	638 200 (4 21 600–9 48 600)	258 200 (1 69 600–3 86 000)	263 500 (170 900–398 600)	1 159 800 (762 100–1 733 200)
China, Hong Kong SAR	No. of data points*	6 (6)	6 (6)	6 (6)	
	Hospital admission rate	4.3 (3.7–4.8)	1.8 (1.5–2)	1.8 (1.6–2.1)	
	Hospital admissions	1300 (1100–1400)	500 (400–600)	500 (400–600)	2300 (2000–2600)
China, Taiwan Province of China	No. of data points*	8 (8)	8 (8)	8 (8)	
	Hospital admission rate	6.9 (5.5–8.6)	2.9 (2.3–3.6)	3 (2.3–3.8)	
	Hospital admissions	7000 (5600–8700)	2900 (2300–3600)	3800 (3000–4800)	13 700 (10 900–17 000)
Denmark	No. of data points*	13 (12)	13 (12)	13 (12)	
	Hospital admission rate	1.9 (1.7–2.1)	0.8 (0.7–0.9)	0.8 (0.7–0.9)	
	Hospital admissions	600 (500–600)	300 (200–300)	300 (200–300)	1100 (1000–1300)
Ecuador	No. of data points*	5 (5)	5 (5)	5 (5)	
	Hospital admission rate	1.9 (1.7–2)	0.8 (0.7–0.9)	0.8 (0.7–0.9)	
	Hospital admissions	3000 (2700–3200)	1200 (1100–1300)	1300 (1100–1400)	5400 (4900–6000)
Egypt	No. of data points*	1 (1)	1 (1)	1 (1)	
	Hospital admission rate	1.4 (1.3–1.4)	0.6 (0.5–0.6)	0.6 (0.5–0.7)	
	Hospital admissions	16 400 (15 800–17 100)	5400 (5000–5900)	5200 (4700–5700)	27 000 (25 400–28 600)
France	No. of data points*	6 (6)	6 (6)	6 (6)	
	Hospital admission rate	1.8 (1.6–1.9)	0.7 (0.7–0.8)	0.8 (0.7–0.8)	
	Hospital admissions	6900 (6500–7400)	2900 (2600–3200)	2900 (2600–3300)	12 800 (11 800–13 900)
Germany	No. of data points*	1 (1)	1 (1)	1 (1)	
	Hospital admission rate	8.9 (8.6–9.3)	3.7 (3.4–4)	3.8 (3.4–4.3)	
	Hospital admissions	33 400 (32 100–34 700)	14 100 (13 000–15 100)	15 800 (14 100–17 800)	63 300 (59 200–67 600)
Ghana	No. of data points*	1 (1)	1 (1)	1 (1)	
	Hospital admission rate	3.2 (3–3.3)	1.3 (1.2–1.4)	1.4 (1.2–1.6)	
	Hospital admissions	12 100 (11 600–12 500)	4500 (4100–4900)	4200 (3600–4900)	20 700 (19 300–22 300)
Guatemala	No. of data points*	4 (4)	4 (4)	4 (4)	
	Hospital admission rate	0.5 (0.4–0.6)	0.2 (0.2–0.3)	0.2 (0.2–0.3)	
	Hospital admissions	1000 (800–1200)	400 (300–500)	400 (300–600)	1900 (1500–2300)
Hungary	No. of data points*	6 (6)	6 (6)	6 (6)	
	Hospital admission rate	2.8 (2.3–3.5)	1.2 (0.9–1.5)	1.2 (1–1.5)	
	Hospital admissions	1300 (1000–1600)	600 (500–700)	600 (500–700)	2400 (2000–3000)
India	No. of data points*	7 (7)	7 (7)	7 (7)	
	Hospital admission rate	4.9 (3.5–7)	2.2 (1.6–3.1)	2.3 (1.6–3.2)	
	Hospital admissions	590 100 (415 600–825 200)	283 400 (202 600–390 600)	289 100 (204 000–403 400)	1 162 600 (822 200–1 619 200)
Indonesia	No. of data points*	3 (3)	3 (3)	3 (3)	
	Hospital admission rate	2.2 (1.3–3.9)	0.9 (0.5–1.6)	1 (0.5–1.7)	
	Hospital admissions	54 100 (30 800–92 800)	21 500 (12 200–37 000)	22 400 (12 600–39 100)	98 100 (55 500–1 68 900)
Iran	No. of data points*	2 (2)	2 (2)	2 (2)	
	Hospital admission rate	0.8 (0.3–2.4)	0.4 (0.1–1)	0.4 (0.1–1.1)	
	Hospital admissions	5800 (2000–15 900)	2200 (700–5900)	2000 (700–5600)	10 000 (3500–27500)
Israel	No. of data points*	1 (1)	1 (1)	1 (1)	
	Hospital admission rate	2 (1.9–2.1)	0.8 (0.8–0.9)	0.9 (0.8–1)	
	Hospital admissions	1600 (1600–1700)	600 (600–600)	600 (500–600)	2800 (2600–3000)
Italy	No. of data points*	9 (9)	9 (9)	9 (9)	
	Hospital admission rate	1.7 (1.5–1.9)	0.7 (0.6–0.8)	0.7 (0.6–0.9)	
	Hospital admissions	4600 (4000–5200)	2000 (1700–2300)	2100 (1700–2500)	8700 (7500–10 000)
Kenya	No. of data points*	23 (22)	23 (22)	23 (22)	
	Hospital admission rate	1.3 (1.1–1.7)	0.6 (0.5–0.7)	0.6 (0.5–0.8)	
	Hospital admissions	9300 (7400–11 400)	3800 (3000–4800)	3600 (2800–4700)	16 700 (13 200–21 000)
Malawi	No. of data points*	1 (1)	1 (1)	1 (1)	
	Hospital admission rate	0.1 (0.1–0.1)	0.1 (0.1–0.1)	0.1 (0.1–0.1)	
	Hospital admissions	400 (400–400)	100 (100–200)	100 (100–100)	700 (600–700)
Mongolia	No. of data points*	5 (5)	5 (5)	5 (5)	
	Hospital admission rate	4.6 (3.5–6)	1.9 (1.4–2.6)	2 (1.5–2.7)	
	Hospital admissions	1600 (1200–2100)	500 (400–700)	400 (300–600)	2500 (1900–3300)
Netherlands	No. of data points*	9 (9)	9 (9)	9 (9)	
	Hospital admission rate	0.6 (0.5–0.6)	0.2 (0.2–0.3)	0.3 (0.2–0.3)	
	Hospital admissions	500 (500–600)	200 (200–300)	300 (200–300)	1000 (900–1100)
New Zealand	No. of data points*	1 (1)	1 (1)	1 (1)	
	Hospital admission rate	1.9 (1.9–2)	0.8 (0.7–0.9)	0.8 (0.7–1)	
	Hospital admissions	600 (600–600)	300 (200–300)	300 (200–300)	1100 (1000–1200)
Nicaragua	No. of data points*	2 (2)	2 (2)	2 (2)	
	Hospital admission rate	1.1 (1–1.2)	0.5 (0.4–0.6)	0.5 (0.4–0.6)	
	Hospital admissions	700 (700–800)	300 (200–400)	300 (200–400)	1300 (1100–1500)
Norway	No. of data points*	2 (2)	2 (2)	2 (2)	
	Hospital admission rate	2 (1.7–2.2)	0.8 (0.7–1)	0.9 (0.7–1)	
	Hospital admissions	600 (500–700)	300 (200–300)	300 (200–300)	1200 (1000–1300)
Oman	No. of data points*	4 (4)	4 (4)	4 (4)	
	Hospital admission rate	1.2 (1.2–1.3)	0.5 (0.5–0.6)	0.5 (0.5–0.6)	
	Hospital admissions	500 (400–500)	200 (100–200)	100 (100–100)	700 (700–800)
Philippines	No. of data points*	6 (6)	6 (6)	6 (6)	
	Hospital admission rate	9 (7.6–10.8)	3.8 (3.1–4.5)	3.9 (3.1–4.8)	
	Hospital admissions	103 000 (86 500–121 800)	40 700 (33 700–48 800)	40 500 (32 600–49 800)	184 200 (152 800–220 400)
Poland	No. of data points*	2 (2)	2 (2)	2 (2)	
	Hospital admission rate	4.4 (3.7–5.1)	1.8 (1.5–2.3)	1.9 (1.5–2.3)	
	Hospital admissions	8400 (7300–9800)	3500 (2800–4300)	3300 (2600–4100)	15 200 (12 700–18 200)
South Africa	No. of data points*	1 (1)	1 (1)	1 (1)	
	Hospital admission rate	7.1 (6.8–7.4)	2.9 (2.7–3.2)	3 (2.7–3.3)	
	Hospital admissions	41 000 (39 300–42 700)	15 800 (14 500–17 200)	14 700 (13 300–16 200)	71 600 (67 200–76 100)
South Korea	No. of data points*	5 (5)	5 (5)	5 (5)	
	Hospital admission rate	3.7 (1.9–7.2)	1.5 (0.8–3)	1.6 (0.8–3.1)	
	Hospital admissions	8400 (4300–16 000)	3500 (1800–6700)	4100 (2100–7900)	16 100 (8200–30 600)
Spain	No. of data points*	15 (15)	15 (15)	15 (15)	
	Hospital admission rate	1.1 (1–1.3)	0.5 (0.4–0.6)	0.5 (0.4–0.6)	
	Hospital admissions	2700 (2400–3000)	1200 (1000–1400)	1100 (900–1300)	4900 (4300–5600)
Sweden	No. of data points*	1 (1)	1 (1)	1 (1)	
	Hospital admission rate	1.1 (1.1–1.1)	0.5 (0.4–0.5)	0.5 (0.4–0.5)	
	Hospital admissions	700 (700–700)	300 (200–300)	300 (200–300)	1200 (1100–1200)
Thailand	No. of data points*	3 (3)	3 (3)	3 (3)	
	Hospital admission rate	5.9 (4–8.6)	2.5 (1.7–3.6)	2.5 (1.7–3.8)	
	Hospital admissions	23 100 (15 700–33 300)	10 300 (6900–15 000)	11 400 (7600–16 700)	44 700 (30 200–64 900)
Tunisia	No. of data points*	1 (1)	1 (1)	1 (1)	
	Hospital admission rate	0.4 (0.4–0.4)	0.2 (0.1–0.2)	0.2 (0.1–0.2)	
	Hospital admissions	400 (400–400)	100 (100–100)	100 (100–100)	600 (600–600)
Uganda	No. of data points*	12 (12)	12 (12)	12 (12)	
	Hospital admission rate	4.1 (3–5.7)	1.7 (1.2–2.4)	1.8 (1.2–2.5)	
	Hospital admissions	28 600 (20 600–38 900)	10 300 (7400–14 200)	9000 (6300–12 500)	47 800 (34 400–65 600)
UK	No. of data points*	35 (35)	35 (35)	35 (35)	
	Hospital admission rate	0.7 (0.6–0.7)	0.3 (0.2–0.3)	0.3 (0.2–0.3)	
	Hospital admissions	2700 (2500–2900)	1100 (900–1200)	1000 (900–1200)	4800 (4400–5400)
Uruguay	No. of data points*	4 (4)	4 (4)	4 (4)	
	Hospital admission rate	5.1 (4.5–5.7)	2.1 (1.8–2.5)	2.2 (1.8–2.6)	
	Hospital admissions	1200 (1100–1300)	500 (400–600)	500 (400–600)	2200 (1900–2600)
USA	No. of data points*	15 (15)	15 (15)	15 (15)	
	Hospital admission rate	1.3 (1.2–1.5)	0.6 (0.5–0.6)	0.6 (0.5–0.7)	
	Hospital admissions	27 300 (24 700–30 100)	11 900 (10 500–13 400)	12 300 (10 600–14 100)	51 500 (45 800–57 600)
By UNICEF region‡					
Asia-Pacific	No. of countries§	9	9	9	9
	Hospital admission rate	5.7 (3.9–8.3)	2.5 (1.7–3.5)	2.5 (1.7–3.7)	3.6 (2.4–5.2)
	Hospital admissions	1 812 400 (1 247 300–2 621 900)	783 300 (541 600–1 127 200)	797 800 (544 000–1 163 000)	3 387 200 (2 328 600–4 903 000)
Sub-Saharan Africa	No. of countries§	5	5	5	5
	Hospital admission rate	3.5 (3–4)	1.4 (1.2–1.7)	1.5 (1.2–1.8)	2.2 (1.9–2.6)
	Hospital admissions	545 100 (474 000–633 000)	199 300 (167 800–237 600)	179 500 (148 200–218 000)	915 400 (782 300–1 079 200)
Eastern Europe and Central Asia¶	No. of countries§	0	0	0	0
	Hospital admission rate	–	–	–	–
	Hospital admissions	–	–	–	–
High income countries	No. of countries§	17	17	17	17
	Hospital admission rate	2.2 (1.9–2.5)	0.9 (0.8–1.1)	1 (0.8–1.2)	1.3 (1.2–1.6)
	Hospital admissions	129 100 (113 600–149 700)	54 600 (46 600–65 000)	57 700 (47 900–70 500)	241 300 (208 200–285 200)
Latin America and Caribbean	No. of countries§	6	6	6	6
	Hospital admission rate	5.1 (4.4–5.8)	2 (1.5–2.6)	2 (1.6–2.5)	3 (2.5–3.6)
	Hospital admissions	264 200 (231 000–301 500)	104 200 (79 800–134 700)	107 600 (87 100–133 000)	472 700 (394 900–565 500)
Middle East and North Africa	No. of countries§	4	4	4	4
	Hospital admission rate	1.2 (0.9–1.7)	0.5 (0.4–0.7)	0.5 (0.4–0.8)	0.7 (0.6–1.1)
	Hospital admissions	52 800 (42 500–77500)	18 700 (14 200–28 800)	17 400 (13 100–27 100)	89 500 (70 400–134 400)
By World Bank income region					
Low	No. of countries§	2	2	2	2
	Hospital admission rate	3.0 (2.2–4.1)	1.2 (0.9–1.7)	1.3 (0.9–1.8)	1.9 (1.4–2.6)
	Hospital admissions	307 800 (223 600–418 500)	112 700 (81 000–154 600)	102 200 (72 400–142 300)	525 100 (378 800–718 500)
Lower middle	No. of countries§	11	11	11	11
	Hospital admission rate	4.6 (3.3–6.4)	2.1 (1.5–2.9)	2.1 (1.5–3)	2.9 (2.1–4.1)
	Hospital admissions	1 404 700 (1 021 900–1 948 700)	617 100 (446 700–855 300)	612 700 (436 100–862 300)	2 608 700 (1 885 700–3 630 800)
Upper middle	No. of countries§	7	7	7	7
	Hospital admission rate	6.3 (4.3–9)	2.6 (1.8–3.8)	2.7 (1.8–3.9)	3.9 (2.6–5.6)
	Hospital admissions	1183300 (819 200–1 708 800)	474 800 (321 600–696 000)	474 900 (321 300–699 000)	2 125 400 (1 456 800–3 092 900)
High	No. of countries§	21	21	21	21
	Hospital admission rate	2.3 (2–2.7)	0.9 (0.8–1.1)	1 (0.8–1.2)	1.4 (1.2–1.7)
	Hospital admissions	154 900 (135 700–180 600)	64 700 (55 000–77 400)	67 900 (56 200–83 200)	286 800 (246 200–340 400)
Global**	Hospital admissions	3 050 800 (2 200 400–4 256 500)	1 269 300 (904 400–1 783 300)	1 257 800 (886 000–1 786 900)	5 546 100 (3 967 500–7 782 700)

*The number in parentheses is the number of imputed data points. A study was counted as an imputed study when it requires imputation for any of the three age groups. Yearly variation was incorporated in the estimates when annual data were available.

†Estimates of hospital admission rates were either derived from meta-analyses (when there were two or more studies) or as reported in one study for each country.

‡Asia-Pacific: the region of South Asia and the region of East Asia and the Pacific were combined due to the insufficient data. Sub-Saharan Africa: the region of Eastern and Southern Africa and the region of West and Central Africa were combined due to insufficient data.

§Number of countries with data.

¶Estimates were not calculated in Eastern Europe and Central Asia as no data were found in this region.

**Global estimates were calculated as the sum of estimates by World Bank income regions.

We identified multiyear (4 years or more) all-cause ALRI hospital admission rates in 21 countries between 1981 and 2017 (online supplemental appendix pp15-16). The trend of all-cause ALRI hospital admission rates varied by geographical locations: we found no significant change in all-cause ALRI hospital admission rates in 70% of these countries, a decrease in Brazil, Canada, France and Kenya, and an increase in Denmark, the Netherlands, and Taiwan, China.

There were 27 studies with data on hCFRs of all-cause ALRI, including 16 studies reporting data for 5–14 years and 12 studies for 5–19 years. hCFRs ranged from 0.05% (95%CI 0.05 to 0.06) to 11.76% (95%CI 7.11 to 18.85) across studies (online supplemental appendix pp24-25). Of the 27 studies, 9 studies had a low risk of bias in study design, and all studies had a low risk of bias in patient groups excluded. Seventeen studies used definitions similar to that we specified, eight studies used less specific definitions, and two studies used more specific definitions. Using all data from a mixture of age groups, we estimated an hCFR of 1.6% (95% CI 0.7 to 4.1), 1.6% (95% CI 0.6 to 4.2) and 0.4% (95% CI 0.2 to 0.6) for low-income and lower middle-income, upper middle-income, and high-income countries, respectively (table 2). These estimates translated to 87 900 (UR 40 300–180 600) all-cause ALRI in-hospital deaths for children and adolescents aged 5–19 years per year globally. By UNICEF regions, the hCFR estimates had wide CIs in the region of Sub-Saharan Africa, Latin America and Caribbean, and Asia-Pacific.

**Table 2:** Estimates of acute lower respiratory infections (ALRI) in-hospital case-fatality ratios (hCFRs, %) and in-hospital mortality in children and adolescents aged 5–19 years by UNICEF region and World Bank income region

Region	No. of studies	hCFR (%)	In-hospital mortality
By UNICEF region†			
Asia-Pacific	5	0.6 (0.2–2.5)	21 000 (5300–87 400)
Sub-Saharan Africa	6	2.1 (0.8–5.6)	19 300 (7200–49 100)
Eastern Europe and Central Asia	0	–	–
High-income countries	8	0.3 (0.1–0.5)	700 (300–1200)
Latin America and Caribbean	4	1.6 (0.4–5.8)	7500 (2100–26 300)
Middle East and North Africa	4	1.3 (0.7–2.3)	1100 (600–2200)
By World Bank income region			
Low income and lower middle income	7	1.6 (0.7–4.1)	51 100 (18 300–126 600)
Upper middle income	8	1.6 (0.6–4.2)	32 500 (11 900–90 400)
High income	12	0.4 (0.2–0.6)	1000 (600–1800)
Global‡			87 900 (40 300–180 600)

*hCFR estimates were from meta-analyses. In-hospital mortality were estimated by combining hCFR estimates and the ALRI hospital admissions for 5–19 years.

†Asia-Pacific: the region of South Asia and the region of East Asia and the Pacific were combined due to the insufficient data. Sub-Saharan Africa: the region of Eastern and Southern Africa and the region of West and Central Africa were combined due to insufficient data.

‡Global estimates were calculated as the sum of estimates by World Bank income region. The point value of the global estimate was slightly different from the sum of the point estimates by region due to the substantial uncertainties in the estimates across regions.

We identified 48 studies (26 countries) with data on influenza-associated ALRI hospital admission rates (table 3). Thirty-three of the 48 studies had a low risk of bias in study design, 47 studies had a low risk in adjustment for healthcare utilisation, and 45 studies had a low risk of bias in patient groups excluded, 14 studies had a low risk of bias in sampling strategy, and 30 studies had a low risk in test methods. Thirteen studies used definitions similar to that we specified, 34 studies used less specific definitions (including 4 studies using hospitalised ICD-coded confirmed influenza respiratory cases), and 1 studies used more specific definitions.

**Table 3:** Estimates of influenza-associated acute lower respiratory infections (ALRI) hospital admission rates (per 1000 persons per year) and hospital admissions in children and adolescents aged 5–19 years, by three age groups, country, UNICEF region and World Bank income region

Country/region		5–9 years	10–14 years	15–19 years	5–19 years
Australia	No. of data points†	7 (7)	7 (7)	7 (7)	
	Hospital admission rate‡	0.1 (0.1–0.2)	0.1 (0–0.1)	0 (0–0.1)	
	Hospital admissions	200 (100–300)	100 (100–200)	0 (0–100)	400 (200–600)
Bolivia	No. of data points†	6 (6)	6 (6)	6 (6)	
	Hospital admission rate‡	8.1 (4.5–14.5)	3.5 (1.6–7.5)	1.7 (0.4–8.2)	
	Hospital admissions	9600 (5400–16800)	4100 (1900–8500)	2000 (400–8800)	15 700 (7700–34 100)
Cambodia	No. of data points†	3 (3)	3 (3)	3 (3)	
	Hospital admission rate‡	0.4 (0.1–2.9)	0.2 (0–1.6)	0.1 (0–1.1)	
	Hospital admissions	800 (100–4800)	300 (0–2400)	100 (0–1500)	1300 (200–8600)
Canada	No. of data points†	1 (1)	1 (1)	1 (1)	
	Hospital admission rate‡	0.1 (0.1–0.1)	<0.05	<0.05	
	Hospital admissions	100 (100–100)	100 (0–100)	<50	200 (200–300)
China	No. of data points†	6 (6)	6 (6)	6 (6)	
	Hospital admission rate‡	1.8 (0.8–3.9)	0.7 (0.3–1.8)	0.4 (0.1–1.1)	
	Hospital admissions	1 56 300 (71 500–330 300)	62 900 (26 000–146 000)	31 700 (10 400–91 800)	250 900 (108 000–568 100)
China, Hong Kong SAR	No. of data points†	7 (7)	7 (7)	7 (7)	
	Hospital admission rate‡	2.7 (2.2–3.3)	1.1 (0.7–1.8)	0.5 (0.3–1.1)	
	Hospital admissions	800 (600–1000)	300 (200–500)	200 (100–300)	1300 (900–1700)
Finland	No. of data points†	1 (1)	1 (1)	1 (1)	
	Hospital admission rate‡	0.2 (0.2–0.2)	0.1 (0.1–0.1)	0 (0–0.1)	
	Hospital admissions	100 (100–100)	<50	<50	100 (100–100)
Germany	No. of data points†	2 (2)	2 (2)	2 (2)	
	Hospital admission rate‡	0.2 (0.1–0.6)	0.1 (0–0.3)	0 (0–0.1)	
	Hospital admissions	900 (400–2200)	400 (100–900)	200 (0–600)	1400 (500–3700)
Ghana	No. of data points†	1 (1)	1 (1)	1 (1)	
	Hospital admission rate‡	0.4 (0.3–0.5)	0.2 (0.1–0.3)	0.1 (0–0.3)	
	Hospital admissions	1600 (1300–1900)	600 (300–1000)	300 (100–900)	2500 (1700–3800)
Greece	No. of data points†	2 (2)	2 (2)	2 (2)	
	Hospital admission rate‡	1 (0.6–1.5)	0.4 (0.2–0.8)	0.2 (0.1–0.6)	
	Hospital admissions	500 (300–700)	200 (100–400)	100 (0–300)	800 (400–1500)
India	No. of data points†	6 (6)	6 (6)	6 (6)	
	Hospital admission rate‡	1 (0.3–3)	0.4 (0.1–1.6)	0.3 (0.1–1.1)	
	Hospital admissions	123 800 (42 300–345 700)	53 000 (13 800–192 500)	34 600 (8900–126 700)	211 500 (65 000–664 900)
Indonesia	No. of data points†	9 (9)	9 (9)	9 (9)	
	Hospital admission rate‡	0.5 (0.3–0.7)	0.2 (0.1–0.3)	0.1 (0–0.2)	
	Hospital admissions	11 400 (7300–17 600)	4600 (2600–7800)	2300 (1000–5300)	18 400 (10 900–30 700)
Ireland	No. of data points†	1 (1)	1 (1)	1 (1)	
	Hospital admission rate‡	1.7 (1.6–1.7)	0.7 (0.4–1.1)	0.3 (0.2–0.7)	
	Hospital admissions	600 (600–600)	200 (200–400)	100 (100–200)	900 (800–1200)
Kenya	No. of data points†	4 (4)	4 (4)	4 (4)	
	Hospital admission rate‡	0.3 (0.1–0.7)	0.1 (0–0.3)	0.1 (0–0.2)	
	Hospital admissions	1900 (700–4900)	800 (300–2100)	300 (100–1100)	3000 (1100–8000)
Malawi	No. of data points†	1 (1)	1 (1)	1 (1)	
	Hospital admission rate‡	<0.05	<0.05	<0.05	
	Hospital admissions	100 (100–100)	0 (0–100)	<50	200 (100–200)
Mongolia	No. of data points†	1 (1)	1 (1)	1 (1)	
	Hospital admission rate‡	0.9 (0.9–0.9)	0.4 (0.3–0.6)	0.2 (0.1–0.4)	
	Hospital admissions	300 (300–300)	100 (100–200)	0 (0–100)	500 (400–600)
Oman	No. of data points†	14 (14)	14 (14)	14 (14)	
	Hospital admission rate‡	0.1 (0–0.2)	0 (0–0.1)	0 (0–0.1)	
	Hospital admissions	0 (0–100)	<50	<50	100 (0–100)
Peru	No. of data points†	1 (1)	1 (1)	1 (1)	
	Hospital admission rate‡	0.3 (0.1–0.7)	0.1 (0–0.8)	0.1 (0–1.6)	
	Hospital admissions	800 (400–1900)	300 (0–2200)	200 (0–3600)	1400 (400–7700)
Philippines	No. of data points†	5 (5)	5 (5)	5 (5)	
	Hospital admission rate‡	1.7 (1–3)	0.7 (0.4–1.3)	0.4 (0.2–0.8)	
	Hospital admissions	19 900 (11 600–33 200)	7900 (4400–14 000)	3800 (1700–8400)	31 600 (17 700–55 700)
Singapore	No. of data points†	1 (1)	1 (1)	1 (1)	
	Hospital admission rate‡	1.2 (1.1–1.2)	0.5 (0.3–0.7)	0.2 (0.1–0.5)	
	Hospital admissions	300 (300–300)	100 (100–200)	100 (0–100)	400 (400–600)
Spain	No. of data points†	6 (6)	6 (6)	6 (6)	
	Hospital admission rate‡	<0.05	<0.05	<0.05	
	Hospital admissions	100 (100–100)	<50	<50	100 (100–200)
Switzerland	No. of data points†	1 (1)	1 (1)	1 (1)	
	Hospital admission rate‡	0.2 (0.2–0.3)	0.1 (0–0.2)	0 (0–0.2)	
	Hospital admissions	100 (100–100)	0 (0–100)	0 (0–100)	100 (100–300)
Thailand	No. of data points†	4 (0)	4 (0)	4 (0)	
	Hospital admission rate‡	0.4 (0.2–0.8)	0.3 (0.2–0.6)	0.1 (0.1–0.2)	
	Hospital admissions	1800 (900–3200)	1300 (700–2300)	500 (300–800)	3600 (2000–6300)
Uganda	No. of data points†	12 (12)	12 (12)	12 (12)	
	Hospital admission rate‡	0.5 (0.4–0.7)	0.2 (0.1–0.3)	0.1 (0–0.2)	
	Hospital admissions	3600 (2600–5100)	1300 (800–2000)	500 (200–1100)	5500 (3600–8200)
UK	No. of data points†	5 (5)	5 (5)	5 (5)	
	Hospital admission rate‡	0.1 (0.1–0.2)	0.1 (0–0.1)	0 (0–0.1)	
	Hospital admissions	500 (300–800)	200 (100–300)	100 (0–200)	800 (500–1300)
USA	No. of data points†	20 (20)	20 (20)	20 (20)	
	Hospital admission rate‡	0.2 (0.2–0.3)	0.1 (0.1–0.1)	0 (0–0.1)	
	Hospital admissions	4700 (3400–6400)	2100 (1400–3000)	1000 (500–1900)	7800 (5300–11 300)
Viet Nam	No. of data points†	5 (5)	5 (5)	5 (5)	
	Hospital admission rate‡	0.2 (0.1–0.4)	0.1 (0–0.2)	0 (0–0.2)	
	Hospital admissions	1700 (1000–2900)	700 (300–1400)	300 (100–1400)	2700 (1400–5700)
By UNICEF region§					
Asia-Pacific	No. of countries¶	9	9	9	9
	Hospital admission rate	1.2 (0.5–2.9)	0.5 (0.2–1.4)	0.3 (0.1–0.9)	0.7 (0.3–1.7)
	Hospital admissions	393 500 (168 600–917 900)	162 000 (59 500–453 400)	91 000 (27 800–291 800)	645 500 (255 400–1 661 100)
Sub-Saharan Africa	No. of countries¶	4	4	4	4
	Hospital admission rate	0.4 (0.2–0.6)	0.1 (0.1–0.3)	0.1 (0–0.2)	0.2 (0.1–0.4)
	Hospital admissions	55 800 (36 100–92 200)	19 900 (10 800–37 800)	8500 (3200–22 800)	83 300 (49 500–151 600)
Eastern Europe and Central Asia**	No. of countries¶	0	0	0	
	Hospital admission rate	–	–	–	–
	Hospital admissions	–	–	–	–
High income countries	No. of countries¶	11	11	11	11
	Hospital admission rate	0.2 (0.2–0.3)	0.1 (0.1–0.2)	0 (0–0.1)	0.1 (0.1–0.2)
	Hospital admissions	13 200 (9400–19 200)	5700 (3500–9200)	2800 (1400–5700)	21 600 (14 300–34 000)
Latin America and Caribbean	No. of countries¶	2	2	2	2
	Hospital admission rate	2.7 (1.5–4.9)	1.1 (0.5–2.8)	0.6 (0.1–3.4)	1.5 (0.7–3.7)
	Hospital admissions	1 41 500 (77 400–253 300)	59 500 (26 000–144 700)	33 100 (6400–182 800)	2 38 300 (112 600–582 200)
Middle East and North Africa	No. of countries¶	1	1	1	1
	Hospital admission rate	0.1 (0.1–0.2)	0 (0–0.1)	0 (0–0.1)	0.1 (0–0.1)
	Hospital admissions	4500 (2300–8600)	1900 (800–3900)	900 (300–2400)	7600 (3700–15 500)
By World Bank income region					
Low	No. of countries¶	2	2	2	2
	Hospital admission rate	0.4 (0.3–0.5)	0.2 (0.1–0.2)	0.1 (0–0.2)	0.2 (0.1–0.3)
	Hospital admissions	39 800 (28 300–55 200)	14 400 (9200–22 200)	6000 (2800–12 800)	60 700 (40 600–90 800)
Lower middle	No. of countries¶	9	9	9	9
	Hospital admission rate	1 (0.4–2.4)	0.4 (0.1–1.3)	0.2 (0.1–0.9)	0.5 (0.2–1.5)
	Hospital admissions	2 95 600 (121 300–738 400)	1 19 100 (39 200–3 79 100)	70 700 (19 800–248 400)	476 100 (176 100–1 346 000)
Upper middle	No. of countries¶	3	3	3	3
	Hospital admission rate	1.7 (0.8–3.6)	0.7 (0.3–1.7)	0.4 (0.1–1.1)	0.9 (0.4–2.1)
	Hospital admissions	323 500 (148 200–682 600)	1 30 100 (54 000–303 600)	64 400 (21 300–190 600)	515 500 (222 300–1 172 300)
High	No. of countries¶	13	13	13	13
	Hospital admission rate	0.2 (0.2–0.3)	0.1 (0.1–0.2)	0 (0–0.1)	0.1 (0.1–0.2)
	Hospital admissions	16 200 (11 700–23 300)	6900 (4300–11 200)	3300 (1600–6800)	26 300 (17 500–41 100)
Global††	Hospital admissions	675 000 (309 400–1 499 400)	2 70 500 (106 700–716 100)	144 400 (45 500–458 600)	1 078 600 (456 500–2 650 200)

*Estimates were derived using incidence-based approach.

†The number in parentheses is the number of imputed data points. A study was counted as an imputed study when it requires imputation for any of the three age groups. Yearly variation was incorporated in the estimates when annual data were available.

‡Estimates of hospital admission rates were either derived from meta-analyses (when there were two or more studies) or as reported in one study for each country.

§Asia-Pacific: the region of South Asia and the region of East Asia and the Pacific were combined due to the insufficient data. Sub-Saharan Africa: the region of Eastern and Southern Africa and the region of West and Central Africa were combined due to insufficient data.

¶Number of countries with data.

**Estimates were not calculated in Eastern Europe and Central Asia as no data were found in this region.

††Global estimates were calculated as the sum of estimates by World Bank income regions. The point values of global estimates were slightly different from the sum of the point estimates by region due to the substantial uncertainties in the estimates across regions.

Influenza-associated ALRI hospital admission rate meta-estimates ranged from 0 to 8.1 (95%CI 4.5 to 14.5) per 1000 persons per year for 5–9 years across 26 countries, from 0 to 3.5 (95% CI 1.6 to 7.5) for 10–14 years, and from 0 to 1.7 (95% CI 0.4 to 8.2) for 15–19 years. Similar to all-cause ALRI hospital admission rates, by World Bank income regions we estimated highest influenza-associated ALRI hospital admission rate in upper middle-income countries (0.9 per 1000 persons per year (95% CI 0.4 to 2.1)), followed by lower middle-income countries (0.5 (95% CI 0.2 to 1.5)), low-income countries (0.2 (95% CI 0.1 to 0.3)), and high-income countries (0.1 (95% CI 0.1 to 0.2)). By UNICEF regions, influenza-associated ALRI hospital admission rates for 5–19 years were higher in Latin America and Caribbean, and Asia-Pacific (0.7–1.5 per 1000 persons per year) compared with other regions (0.1–0.2) except for Eastern Europe and Central Asia where estimates were not calculated due to the unavailability of data. Globally, we estimated 1 078 600 (UR 456 500–2 650 200) influenza-associated ALRI hospital admissions among 5–19 years annually, accounting for 19% (about 1.1 million of 5.5 million) of all-cause ALRI hospital admissions in this age group. About 63% (675 000/1 078 600), 25% (270 500), and 13% (144 400) of the hospital admissions occurred among 5–9 years, 10–14 years and 15–19 years, respectively.

We identified 54 studies with data on proportion positives of respiratory syncytial virus-associated ALRI, including 3 studies for 5–9 years, 34 studies for 5–14 years, and 17 studies for 5–19 years. The proportion positives of respiratory syncytial virus-associated ALRI ranged from 0% to 47.3% (95% CI 45.2 to 49.4) across studies (online supplemental appendix pp34-36). The meta-estimates of proportion positives of respiratory syncytial virus-associated ALRI were similar by World Bank income regions (3.9%–4.5%) (table 4). Based on these estimates, the annual global number of respiratory syncytial virus-associated ALRI hospital admissions was 231 800 (UR 1 42 700–3 73 200) among 5–19 years. By UNICEF regions, the estimated proportion positives of respiratory syncytial virus was higher in Sub-Saharan Africa, Eastern Europe and Central Asia, and Latin America and Caribbean than other regions. The estimates in these regions were based on limited data, therefore need to be verified with additional data.

**Table 4:** Estimates of proportion positives of acute lower respiratory infections (ALRI) associated with respiratory syncytial virus, human metapneumovirus and human parainfluenza virus, and the hospital admissions in children and adolescents by World Bank income region and UNICEF region

Virus (age group)	Region	No. of studies	Proportion*	Hospital admissions
Respiratory syncytial virus (5–19 years)†	**By UNICEF region‡**			
	Asia-Pacific	28	3.4 (2.1–5.7)	115 100 (58 900–217 100)
	Sub-Saharan Africa	5	6.9 (5.9–8.0)	62 700 (50 000–78 400)
	Eastern Europe and Central Asia§	2	9.2 (4.5–19.0)	–
	High-income countries	11	3.2 (1.4–7.2)	7700 (3300–17 000)
	Latin America and Caribbean	3	6.1 (3.3–11.4)	28 600 (15 100–54 500)
	Middle East and North Africa	5	6.4 (1.8–23.3)	6000 (1600–22 800)
	**By World Bank income region**			
	Low and lower middle	15	4.0 (2.4–6.8)	125 000 (66 800–222 600)
	Upper middle	27	4.5 (2.8–7.2)	94 500 (51 100–170 200)
	High	12	3.9 (1.6–9.2)	10 800 (4800–27 600)
	**Global¶**			231 800 (142 700–373 200)
Human metapneumovirus (5–14 years)**	**By UNICEF region**			
	Asia-Pacific	17	1.3 (0.7–2.5)	33 700 (15 400–72 000)
	Sub-Saharan Africa	3	3.1 (2–4.7)	22 500 (14 000–34 300)
	Eastern Europe and Central Asia	1	–	–
	High-income countries	3	3.8 (1.8–8.1)	6900 (3300–14 400)
	Latin America and Caribbean	0	–	–
	Middle East and North Africa	1	–	–
	**By World Bank income region**			
	Low and lower middle	6	3.1 (1.6–5.9)	74 000 (35 800–145 700)
	Upper middle	14	1.3 (0.6–2.5)	20 500 (9500–44 400)
	High	3	3.8 (1.8–8.1)	8100 (4000–18 500)
	**Global**			105 500 (57 200–181 700)
Human parainfluenza virus (5–14 years)	**By UNICEF region**			
	Asia-Pacific	19	3 (1.9–4.6)	75 700 (40 600–137 200)
	Sub-Saharan Africa	3	3.9 (2.7–5.7)	28 800 (18 700–42 300)
	Eastern Europe and Central Asia	0	–	–
	High income countries	5	2.9 (1.1–7.6)	5200 (2100–12 900)
	Latin America and Caribbean	0	–	–
	Middle East and North Africa	0	–	–
	**By World Bank income region**			
	Low and lower middle	5	2.6 (1–6.7)	61 500 (21 300–158 500)
	Upper middle	17	3.3 (2.2–5.1)	54 400 (30 200–94 700)
	High	5	2.9 (1.1–7.6)	6100 (2500–17 300)
	**Global**			124 800 (67 300–228 500)

*Estimates from meta-analyses.

†Proportion positives were estimated using data from mixed age groups. Three studies reported data for 5–9 years, 33 studies for 5–14 years, and 16 studies for 5–19 years.

‡Asia-Pacific: the region of South Asia and the region of East Asia and the Pacific were combined due to the insufficient data. Sub-Saharan Africa: the region of Eastern and Southern Africa and the region of West and Central Africa were combined due to insufficient data.

§We were unable to calculate respiratory syncytial virus hospital admissions in the region of Eastern Europe and Central Asia because of the lack of data on all-cause ALRI hospital admissions.

¶Global estimate was calculated as the sum of estimates by World Bank income regions. The point values of global estimates were slightly different from the sum of the point estimates by region due to the substantial uncertainties in the estimates across regions.

**Proportion positives were estimated using data for 5–14 years.

††Proportion positives were estimated using data for 5–14 years.

We identified 32 studies with data on proportion positives of human metapneumovirus-associated ALRI, and 38 studies for proportion positives of human parainfluenza-virus-associated ALRI (online supplemental appendix pp37-40). Using the proportion-based approach, an estimated 105 500 (UR 57 200–181 700) human metapneumovirus-associated ALRI hospital admissions and 124 800 (UR 67 300–228 500) human parainfluenza-virus-associated ALRI hospital admissions occurred in children and adolescents aged 5–14 years annually (table 4).

In a sensitivity analysis, we excluded two potential outlier studies with the highest rates, and the estimate of global all-cause ALRI hospital admissions (5.1 million (UR 3.9–6.8) for 5–19 years) was similar to the main analysis (online supplemental appendix p10). The estimated global influenza-associated ALRI hospital admissions was 811 800 (UR 4 00 300–1 867 400) when excluding two potential outlier studies with the highest rates, and was 1 077 100 (UR 4 57 200–2 643 100) when excluding studies using the definition of “ICD-coded influenza-confirmed respiratory hospital admissions” (online supplemental appendix pp12-13). Based on proportion positives of influenza-associated ALRI in 70 studies, the estimate of global influenza-associated ALRI hospital admissions was 796 600 (UR 525 900–1 177 500) using the proportion-based approach (online supplemental appendix p14).

## Discussion

Globally, an estimated 5.5 million (UR 4.0–7.8) all-cause ALRI hospital admissions occurred annually between 1995 and 2019 in children and adolescents aged 5–19 years, leading to 87 900 (UR 40 300–180 600) in-hospital deaths. Influenza virus and respiratory syncytial virus were estimated to account for 19% (1 078 600/5 546 100) and 4% (231 800) of ALRI hospital admissions in the 5–19 years age group. Human metapneumovirus (105 500) and human parainfluenza virus (124 800) accounted for about 2% of ALRI hospital admissions in the 5–14 years age group globally. Age-stratified results suggest that an estimated 55% (3 050 800) of ALRI hospital admissions and 63% (675 000) of influenza-associated ALRI hospital admissions occurred in children aged 5–9 years. By adding a country-level layer to our analytical model, we also report national estimates of all-cause ALRI hospital admissions for 39 countries and influenza-associated ALRI hospital admissions for 26 countries, accounting for 57% and 52% of the global population 5–19 years of age. We found that all-cause ALRI hospital admission rates for 5–19 years were similar over years in 15 of 21 countries where four or more consecutive years’ data were identified, and most of the countries are from World Bank upper middle-income and high-income regions. Additional multiyear data from low-income and lower middle-income countries are needed to understand the trend of all-cause ALRI hospital admission rates in these regions.^13^

Several factors could have affected the national, regional and global burden estimates, including the lack of data and the presence of methodological differences between studies. For many countries, data on all-cause ALRI hospital admissions and deaths and influenza-specific hospital admissions for 5–19 years are not available in published reports. In particular, the large uncertainty in our global all-cause ALRI in-hospital mortality estimate (87 900 (UR 40 300–180 600)) reflects the difference in hCFRs of all-cause ALRI between studies and the paucity of hCFR data, especially the lack of data by narrow age groups. By region, the paucity of data is most extreme in World Bank low-income region and in the UNICEF regions of Eastern Europe and Central Asia, South Asia, and West and Central Africa. Estimates for these regions were based on extrapolation of estimates in neighbouring regions, and could have been biased. For example, estimates for the region of West and Central Africa were based on estimates for the region of Eastern and Southern Africa; the estimates in the two regions could have been different given differences in the disease epidemiology (eg, seasonal variations, HIV prevalence). About 60%–70% of the country-specific estimates were based on subnational research studies, which might not be generalisable to the entire country. Additionally, all-cause and influenza-specific ALRI hospital admissions for some countries were estimated using historic data (eg, Germany, Israel and New Zealand for all-cause ALRI; Finland, Greece and Switzerland for influenza-associated ALRI), therefore need to be interpreted with caution.

Differences in case definitions between studies are noted, including less specific definitions (eg, hospitalised acute respiratory infections with/without fever), and more specific definitions (eg, hospitalised chest X-ray confirmed pneumonia). The heterogeneity in case definitions is more pronounced between studies reporting influenza-specific hospital admission rates. For example, 60% of the included studies used ‘hospitalised acute respiratory infections’ and severe acute respiratory infections as defined by WHO (or hospitalised acute respiratory infection with fever) and a further 10% used hospitalised ICD-coded influenza-confirmed respiratory hospital admissions.^14^ In a sensitivity analysis, exclusion of the studies using the least specific case definition (eg, hospitalised ICD-coded influenza-confirmed respiratory hospital admissions) yielded an estimate of global influenza-associated ALRI hospital admissions similar to that in the main analysis. Further restriction by case definitions in analyses was impossible, and the WHO definition of severe acute respiratory infections, developed for use in influenza surveillance in resource-limited settings, can be used more frequently in future influenza studies.^14^ Generally, the use of the less specific definitions could have caused an overestimation of influenza-associated ALRI hospital admissions. On the other hand, influenza-associated ALRI hospital admissions could have been underestimated due to the underdetection of influenza virus and the use of non-polymerase chain reaction (non-PCR) tests. Although we have adjusted for underdetection, levels of underdetection are unavailable in 33% of the included studies, likely causing an underestimation of the influenza-associated ALRI hospital admissions. Additionally, the adjustment is based on the assumption that the proportion positives of influenza virus in patients who were tested was the same as that in those untested. The estimates could have been biased as the assumption may not hold in 38% of the included studies. Thirty-eight per cent of the included studies used culture, immunofluorescence, rapid influenza test and mixed methods, which could have caused an underestimation to influenza hospital admissions as they have lower sensitivity and similar specificity compared with PCR.^15^ Most of the virus-specific data are from 2000 onwards, and the lack of data for the period before 2000 could have biased our estimates.

Countries in the region of Sub-Saharan Africa have the highest HIV prevalence in young people.^16^ The analyses by UNICEF regions show that about 5.1 million all-cause ALRI hospital admissions and 996 000 influenza-associated ALRI hospital admissions occurred globally excluding the region of Eastern Europe and Central Asia (96% of the global population 5–19 years of age), suggesting that our estimates of global all-cause and influenza-associated ALRI hospital admissions are generally unaffected by HIV.

Our estimate of global influenza-associated ALRI hospital admissions for 5–19 years is lower than the IHME estimate for 2017 (which was about 1.9 million influenza-attributable hospital admissions).^2^ Our estimate indicates that 862 900 ALRI hospital admissions could be attributed to influenza after accounting for the same influenza-specific causal fraction as used by IHME (80%). In our analysis, influenza-specific hospital admissions were modelled independently from all-cause ALRI hospital admission estimates, while the IHME estimate for influenza was modelled on ALRI hospital admission estimates. Despite the higher number of influenza-specific hospital admissions by IHME, their estimated proportion of influenza-positive hospital admissions in all-cause ALRI hospital admissions (about 11%) was lower than our estimate (19%). Therefore, the higher estimate by IHME is speculated to be largely driven by the underlying estimate of all-cause ALRI hospital admissions that they used. Further exploration of the difference is impossible as it requires additional information on the estimation of all-cause ALRI hospital admissions by IHME, specifically for 5–19 years.

Viruses are usually isolated in upper respiratory tract samples. Isolation of viruses in upper respiratory tract does not indicate a causal relationship as viruses can also be isolated in upper respiratory tract of healthy children. According to a systematic review in children under 5 years, the attributable fraction in influenza-positive ALRI is 80%; the fraction is 90% for respiratory syntactical virus, 73% for human metapneumovirus-positive ALRI, and 70% for human parainfluenza virus-positive ALRI.^17^ After accounting for the attributable fraction in our estimates for laboratory confirmed cases of ALRI positive for these 4 viruses, about 16% of all-cause ALRI hospital admissions can be attributed to influenza virus (862 900) and 4% attributable to respiratory syntactical virus (208 600) in 5–19 years. Using the same approach, 1%–2% of all-cause ALRI hospital admissions are attributable to human metapneumovirus (77 000) and human parainfluenza virus (87 400) in 5–14 years. A comparison between global all-cause and virus-specific ALRI estimates for 5–19 years and those for children under 5 years suggests that the estimates for 5–19 years (by 5-year age intervals where available) are at least 65% lower except for influenza-associated hospital admissions in 5–9 year olds (675 000) where it is only 20% lower than for children under 5 years (870 000).^5 13 18^

## Conclusions

Our estimates provide evidence to support evaluation of all-cause ALRI intervention programmes for 5–19 years. In particular, the estimated high proportion of identification of influenza (19%) in all-cause ALRI hospital admissions for 5–19 years, especially in 5–14 years (22%) indicates the potential benefit of influenza vaccination in this age group.^19 20^ In addition to vaccines, a decrease in the prevalence of risk factors (eg, HIV and malnutrition) may also contribute to the reduction of all-cause ALRI burden in this age group.^21–24^ To improve the precision of the burden estimates and to track trends over time, more high-quality data are needed. Our regional and global estimates were based on extrapolation of available data from limited countries. The paucity of data as well as the low hospital admission rates in low-income countries reflects the limited access to healthcare, lack of high-quality healthcare systems, and the limited diagnostic capacity, which present a challenge to both the reduction and tracking of ALRI burden in this setting. Given these constraints, it is likely that the number of influenza-associated ALRI cases requiring hospital admission is underestimated in low-income countries. The data gaps for all-cause ALRI burden can be reduced as high-quality electronic healthcare registration systems are developed, and data are increasingly reported in places where such systems have been present.^25^ Increasing the diagnostic capacity in low-income and lower middle-income countries would improve the availability of viral burden data.^26^ The imputation by 5-year age groups was based on data from a small proportion of studies (nine studies for ALRI and eight studies for influenza-associated ALRI; online supplemental appendix p7). Increased reporting of data by standardised 5 year age groups will help improve estimates of all-cause and virus-specific ALRI hospital admissions, and allow for estimation of ALRI in-hospital deaths by age groups. Assessment of the trend of all-cause ALRI burden requires more than multiyear hospital admission rates; collection and reporting of concurrent hCFR data will help quantify the progress in reducing ALRI mortality in 5–19 years of age and improve ALRI mortality estimates. In terms of methodology, standardisation of case definitions, and assessment and reporting of underdetection of viruses will help improve all-cause and virus-specific ALRI burden estimates. Using existing influenza surveillance systems to incorporate testing for other viruses may help fill the larger data gaps, we observed for non-influenza viruses (ie, respiratory syncytial virus, human metapneumovirus and human parainfluenza virus). Our study includes the four viruses as among others, they are shown to be the viruses most strongly associated with ALRI in children under 5 years and therefore likely to result in substantial disease burden in older children as well.^3^ There are other viruses that are associated with ALRI (eg, adenovirus, rhinovirus, enterovirus and seasonal coronavirus), and the burden of these viruses needs to be investigated in future studies.

## Data Availability

Data are available in a public, open access repository. All data relevant to the study are included in the article or uploaded as supplementary information. Details of studies included in this review and references of these studies are uploaded as supplementary information. More details will be uploaded to Edinburgh DataShare (https://datashare.ed.ac.uk/) after publication.

## References

[1] Vos T, Lim SS, Abbafati C, et al. Global burden of 369 diseases and injuries in 204 countries and territories, 1990-2019: a systematic analysis for the global burden of disease study 2019. Lancet 2020;396:1204–22. 10.1016/S0140-6736(20)30925-9 33069326 PMC7567026

[2] GBD 2017 Influenza Collaborators. Mortality, morbidity, and hospitalisations due to influenza lower respiratory tract infections, 2017: an analysis for the global burden of disease study 2017. Lancet Respir Med 2019;7:69–89. 10.1016/S2213-2600(18)30496-X 30553848 PMC6302221

[3] Pneumonia Etiology Research for Child Health (PERCH) Study Group. Causes of severe pneumonia requiring hospital admission in children without HIV infection from Africa and Asia: the PERCH multi-country case-control study. Lancet 2019;394:757–79. 10.1016/S0140-6736(19)30721-4 31257127 PMC6727070

[4] Peterson J, Welch V, Losos M. The Newcastle-Ottawa scale (NOS) for assessing the quality of nonrandomised studies in meta-analyses, 2011.

[5] Wang X, Li Y, O'Brien KL, et al. Global burden of respiratory infections associated with seasonal influenza in children under 5 years in 2018: a systematic review and modelling study. Lancet Glob Health 2020;8:e497–510. 10.1016/S2214-109X(19)30545-5 32087815 PMC7083228

[6] United Nations Department of Economic and Social Affairs Population Division. World population prospects, 2019.

[7] Rücker G, Krahn U, König J, et al. Network Meta-Analysis using Frequentist Methods. In: R package version 1.2-1, 2020.

[8] The World Bank. World bank country and lending groups, 2019.

[9] United Nations Inter-agency Group for Child Mortality Estimation (UN IGME). Levels & trends in child mortality: report 2020, estimates developed by the United Nations inter-agency group for child mortality estimation. New York: United Nations Children’s Fund, 2020.

[10] Stijnen T, Hamza TH, Ozdemir P. Random effects meta-analysis of event outcome in the framework of the generalized linear mixed model with applications in sparse data. Stat Med 2010;29:3046–67. 10.1002/sim.4040 20827667

[11] Li Y, Johnson EK, Shi T, et al. National burden estimates of hospitalisations for acute lower respiratory infections due to respiratory syncytial virus in young children in 2019 among 58 countries: a modelling study. Lancet Respir Med 2021;9:175–85. 10.1016/S2213-2600(20)30322-2 32971018

[12] Stevens GA, Alkema L, Black RE, et al. Guidelines for accurate and transparent health estimates reporting: the gather statement. Lancet 2016;388:e19–23. 10.1016/S0140-6736(16)30388-9 27371184

[13] McAllister DA, Liu L, Shi T, et al. Global, regional, and national estimates of pneumonia morbidity and mortality in children younger than 5 years between 2000 and 2015: a systematic analysis. Lancet Glob Health 2019;7:e47–57. 10.1016/S2214-109X(18)30408-X 30497986 PMC6293057

[14] Fitzner J, Qasmieh S, Mounts AW, et al. Revision of clinical case definitions: influenza-like illness and severe acute respiratory infection. Bull World Health Organ 2018;96:122–8. 10.2471/BLT.17.194514 29403115 PMC5791775

[15] Vemula SV, Zhao J, Liu J, et al. Current approaches for diagnosis of influenza virus infections in humans. Viruses 2016;8:96. 10.3390/v8040096 27077877 PMC4848591

[16] Unaids. AIDSinfo, 2020. Available: https://aidsinfo.unaids.org/ [Accessed 1 Sep 2020].

[17] Shi T, McLean K, Campbell H, et al. Aetiological role of common respiratory viruses in acute lower respiratory infections in children under five years: a systematic review and meta-analysis. J Glob Health 2015;5:010408. 10.7189/jogh.05.010408 26445672 PMC4593292

[18] Wang X, Li Y, Deloria-Knoll M, et al. Global burden of acute lower respiratory infection associated with human metapneumovirus in children under 5 years in 2018: a systematic review and modelling study. Lancet Glob Health 2021;9:e33–43. 10.1016/S2214-109X(20)30393-4 33248481 PMC7783516

[19] Jefferson T, Smith S, Demicheli V, et al. Assessment of the efficacy and effectiveness of influenza vaccines in healthy children: systematic review. Lancet 2005;365:773–80. 10.1016/S0140-6736(05)17984-7 15733718

[20] Rolfes MA, Flannery B, Chung JR, et al. Effects of influenza vaccination in the United States during the 2017-2018 influenza season. Clin Infect Dis 2019;69:1845–53. 10.1093/cid/ciz075 30715278 PMC7188082

[21] Peterson I, Bar-Zeev N, Kennedy N, et al. Respiratory virus-associated severe acute respiratory illness and viral clustering in Malawian children in a setting with a high prevalence of HIV infection, malaria, and malnutrition. J Infect Dis 2016;214:1700–11. 10.1093/infdis/jiw426 27630199 PMC5341080

[22] Cohen C, Walaza S, Moyes J, et al. Epidemiology of severe acute respiratory illness (SARI) among adults and children aged ≥5 years in a high HIV-prevalence setting, 2009-2012. PLoS One 2015;10:e0117716. 10.1371/journal.pone.0117716 25706880 PMC4337909

[23] Macpherson L, Ogero M, Akech S, et al. Risk factors for death among children aged 5-14 years hospitalised with pneumonia: a retrospective cohort study in Kenya. BMJ Glob Health 2019;4:e001715. 10.1136/bmjgh-2019-001715 PMC6730574 31544003

[24] Caleyachetty R, Thomas GN, Kengne AP, et al. The double burden of malnutrition among adolescents: analysis of data from the global school-based student health and health behavior in school-aged children surveys in 57 low- and middle-income countries. Am J Clin Nutr 2018;108:414–24. 10.1093/ajcn/nqy105 29947727

[25] Kruk ME, Gage AD, Joseph NT, et al. Mortality due to low-quality health systems in the universal health coverage era: a systematic analysis of amenable deaths in 137 countries. Lancet 2018;392:2203–12. 10.1016/S0140-6736(18)31668-4 30195398 PMC6238021

[26] Okomo U, Idoko OT, Kampmann B. The burden of viral respiratory infections in young children in low-resource settings. Lancet Glob Health 2020;8:e454–5. 10.1016/S2214-109X(20)30037-1 32087816

