# Supplementary material for: Global hospital admissions and in-hospital mortality associated with all-cause and virus-specific acute lower respiratory infections in children and adolescents aged 5–19 years between 1995 and 2019: a systematic review and modelling study
Source: BMJ Glob Health. 2021 Jul 14;6(7):e006014. doi: 10.1136/bmjgh-2021-006014 (PMC8281096; doi:10.1136/bmjgh-2021-006014)
Supplement: Supplementary data [file bmjgh-2021-006014supp001.pdf]

Supplementary materials for “Global hospital admissions and in-hospital mortality associated with all-cause and virus-specific acute lower respiratory infections in children and adolescents aged 5-19 years between 1995 and 2019: a systematic review and modelling study”

# 1 Contents

Appendix 1 Search strategy ........ 1
Appendix 2 Data preparation and imputation........ 5
Appendix 3 Sensitivity analyses ........ 8
Appendix 4 Change of hospital admission rates of ALRI over time........ 15
Appendix 5 Details of included studies........ 18
Appendix 6 Assessment of risk of bias........ 44

## Appendix 1 Search strategy

**Medline (Ovid)**

| # | Search |
|---|---|
| | 1. clinical pneumonia.mp.<br>2. severe pneumonia.mp.<br>3. alri.mp.<br>4. lower respiratory infection$.mp.<br>5. exp Pneumonia/ or pneumonia.mp.<br>6. exp Bronchiolitis/ or Bronchiolitis, Viral/ |
| | 7. exp Influenza, Human/<br>8. exp Influenzavirus B/ or exp Influenzavirus A/ or exp Influenzavirus C/<br>9. *Influenza Vaccines/ or *Influenza A virus/ or *Influenza, Human/<br>10. exp Respiratory Syncytial Viruses/ or exp Respiratory Syncytial Virus Infections/ or respiratory syncytial virus.mp.<br>11. respiratory syncytial virus*.mp.<br>12. exp Parainfluenza Virus 1, Human/ or exp Parainfluenza Virus 2, Human/ or exp Parainfluenza Virus 3, Human/ or exp Parainfluenza Virus 4, Human/ or exp Parainfluenza virus infection/<br>13. parainfluenza virus.mp. or piv.mp.<br>14. metapneumovirus.mp. or exp metapneumovirus/ or MPV.mp.<br>15. respiratory virus.mp. |
| | 16. disease burden.mp.<br>17. exp morbidity/ or exp incidence/ or exp prevalence/ or exp proportion/ or exp general practice/ or exp outpatient/ or exp clinic /or exp hospitalization/ or exp hospital admission/ or exp hospital admission/ or exp mortality/ or exp death |
| 18 | 1 or 2 or 3 or 4 or 5 or 6 or 7 or 8 or 9 or 10 or 11 or 12 or 13 or 14 or 15 |
| 19 | 16 or 17 |
| 20 | 18 and 19 |
| 21 | limit 21 to (humans and yr="1995 - 2019" and ("child (6 to 12 years)" or "adolescent (13 to 18 years)")) |
| 22 | ep.fs. |
| 23 | 21 and 22 |

**Embase (Ovid)**

| # | Search |
|---|---|
| | 1. clinical pneumonia.mp.<br>2. severe pneumonia.mp.<br>3. alri.mp.<br>4. exp lower respiratory tract infection/ or lower respiratory infection$.mp.<br>5. exp Pneumonia/ or pneumonia.mp.<br>6. exp BRONCHIOLITIS/ or VIRAL BRONCHIOLITIS/ |
| | 7. exp Influenza virus A/ or exp influenza/ or exp Influenza virus/ or exp Influenza virus B/<br>8. exp parainfluenza virus infection/ or para influenza virus.mp. or parainfluenza virus.mp. or parainfluenzavirus.mp. or virus,parainfluenza.mp. or PIV.mp.<br>9. exp metapneumovirus/ or exp metapneumovirus infection/ or mpv.mp.<br>10. respiratory syncytial virus.mp. or exp Respiratory syncytial pneumovirus/ or exp respiratory syncytial virus infection/ or respiratory syncytial virus*.mp.<br>11. respiratory virus.mp. |
| | 12. exp incidence/ or exp prevalence/ or exp morbidity/ or exp general practice/ or exp outpatient/ or exp proportion/ or exp hospital admission/ or exp hospitalization/ or exp death/ or exp mortality/<br>13. disease burden.mp. |
| 14 | 1 or 2 or 3 or 4 or 5 or 6 or 7 or 8 or 9 or 10 or 11 |
| 15 | 12 or 13 |
| 16 | 14 and 15 |
| 17 | limit 16 to (human and yr="1995 - 2019" and (school child <7 to 12 years> or adolescent <13 to 17 years>)) |
| 18 | ep.fs. |
| 19 | 17 and 18 |

**Global Health (Ovid)**

| # | Search |
|---|---|
| | 1. clinical pneumonia.mp.<br>2. severe pneumonia.mp.<br>3. alri.mp.<br>4. exp lower respiratory tract infection/ or lower respiratory infection$.mp.<br>5. exp Pneumonia/ or pneumonia.mp.<br>6. exp BRONCHIOLITIS/ or VIRAL BRONCHIOLITIS/ |
| | 7. exp Influenza virus A/ or exp influenza/ or exp Influenza virus/ or exp Influenza virus B/<br>8. exp parainfluenza virus infection/ or para influenza virus.mp. or parainfluenza virus.mp. or parainfluenzavirus.mp. or virus,parainfluenza.mp. or PIV.mp.<br>9. exp metapneumovirus/ or exp metapneumovirus infection/ or mpv.mp.<br>10. respiratory syncytial virus.mp. or exp Respiratory syncytial pneumovirus/ or exp respiratory syncytial virus infection/ or respiratory syncytial virus*.mp.<br>11. respiratory virus.mp. |
| | 12. exp incidence/ or exp morbidity/ or exp hospital admission/ or exp general practice/ or exp outpatient/ or exp clinic/ or exp hospital admission/ or exp hospitalization/ or exp prevalence/ or exp proportion/ or exp mortality/ or exp death/<br>13. disease burden.mp. |
| 14 | (Children and adolescents or adolescent).mp. |
| 15 | 1 or 2 or 3 or 4 or 5 or 6 or 7 or 8 or 9 or 10 or 11 |
| 16 | 12 or 13 |
| 17 | 14 and 15 and 16 |
| 18 | limit 17 to yr="1995 - 2019" |



Wang X, *et al. BMJ Global Health* 2021; 6:e006014. doi: 10.1136/bmjgh-2021-006014

**CINAHL**

| # | Search |
|---|---|
| | SU pneumonia OR SU pneumonia virus$ OR SU pneumonia, bacterial OR SU bronchiolitis OR SU lower respiratory infection OR SU respiratory syncytial virus OR SU influenza, human OR SU parainfluenza OR SU metapneumovirus |
| | MH disease burden OR MH incidence OR MH prevalence OR MH morbidity OR MH mortality OR MH hospitalization OR MH hospital admission OR MH outpatient OR MH clinic |
| | Limit to 1995 – 2019<br>Limit to child: 6-12 years and adolescent: 13-18 years |

**Web of Science**

Topic =(lower respiratory infection OR pneumonia OR bronchiolitis OR influenza virus OR parainfluenza virus OR metapneumovirus OR respiratory syncytial virus OR respiratory aetiology)
AND Topic=(school aged OR adolescent OR teenager OR teen OR older children and adolescents)
AND Topic=(disease burden OR incidence OR prevalence OR morbidity OR mortality OR death OR hospital admission OR outpatient OR clinic OR general practice)
From 1995 to 2019
4031 papers

**Global Index Medicus, including LILACS regional index.**

| # | Search |
|---|---|
| | Title, abstract, subject: pneumonia OR bronchiolitis OR lower respiratory tract infection OR influenza OR respiratory syncytial virus OR metapneumovirus OR parainfluenza virus<br>AND Title, abstract, subject: children and adolescents OR adolescent<br>AND Title, abstract, subject: incidence OR prevalence OR proportion OR hospital admission OR mortality OR disease burden<br>1995 - 2019 |

**CNKI**
Topic =respiratory tract infections OR pneumonia OR bronchiolitis OR influenza virus OR respiratory syncytial virus OR metapneumovirus OR parainfluenza virus OR respiratory aetiologies
AND
Topic = adolescent OR teen OR school-age OR children and adolescents
1995-2019

**Wanfang**
Topic = respiratory tract infections OR pneumonia OR bronchiolitis OR influenza virus OR respiratory syncytial virus OR metapneumovirus OR parainfluenza virus OR respiratory aetiologies
AND
Topic = adolescent OR teen OR school-age OR children and adolescents
AND
Topic = prevalence OR incidence OR hospital admission OR mortality
1995-2019

**CQvip**
Topic = respiratory tract infections OR pneumonia OR bronchiolitis OR influenza virus OR respiratory syncytial virus OR metapneumovirus OR parainfluenza virus OR respiratory aetiologies
AND
Topic = adolescent OR teen OR school-age OR children and adolescents OR primary school OR secondary school
AND
Topic = prevalence OR incidence OR hospital admission OR outpatient OR mortality OR burden OR epidemiology OR aetiological study
1995-2019

**Google search (200 search results)**
All these words: Child AND pneumonia
Any of these words: influenza OR respiratory syncytial virus OR metapneumovirus OR parainfluenza OR etiology AND filetype:pdf
Time: ~ to 2019.

## Appendix 2 Data preparation and imputation

For hospital admission rates of ALRI and influenza-associated ALRI, we extracted the number of cases and population-at-risk per study. Rates were adjusted for healthcare utilisation where available. Influenza-specific rates were additionally adjusted for levels of testing where available.

We imputed data for any of the three age bands (5-9 years, 10-14 years, or 15-19 years) where missing. Since data on rate ratios between the three age bands were only available for hospital admission rates of ALRI and influenza-associated ALRI, rates and the number of cases were imputed for the two outcomes. We used a multiple imputation approach was as used previously for influenza virus burden estimation.[1,2] The imputation was done at the study level following three steps: (1) imputing the denominator; (2) imputing the rate; (3) calculating the case number by combining the denominator and rate. Details of each step of imputation are presented below.

(1) We imputed denominator for any of the three age bands using denominators reported in each study and the country-level population structure by single year of age.[3]

(2) We imputed rates using a multiple imputation approach by assuming the rates were missing at random.[1,2] Figure S2.1 shows the imputation of rates. First, we pooled rate ratios between three age bands using the network meta-analysis, with the age band 5-9 years as the reference.[4] Second, the pooled rate ratios were assumed to follow log-normal distributions, and 10 samples of each rate ratio were simulated. Third, we estimated rate ratios for any other age bands (e.g., 5-14 years versus 5-9 years) using the rate ratios between the three age bands and the UN Population Division country population structures by single year of age (Table S3.1).[3] We assumed the hospital admission rate was the similar within each five-year age band. Fourth, with samples of rate ratios and the observed rates in each study, we calculated 10 samples of rates for each of the three age bands for each study. Fifth, numbers of cases for each study were calculated using the denominator and imputed rates. Following this strategy, 10 datasets were imputed for each of the three age bands. Sixth, meta-analysis was done for each dataset, and the meta–estimates were combined together using the Rubin's rules.[5,6]

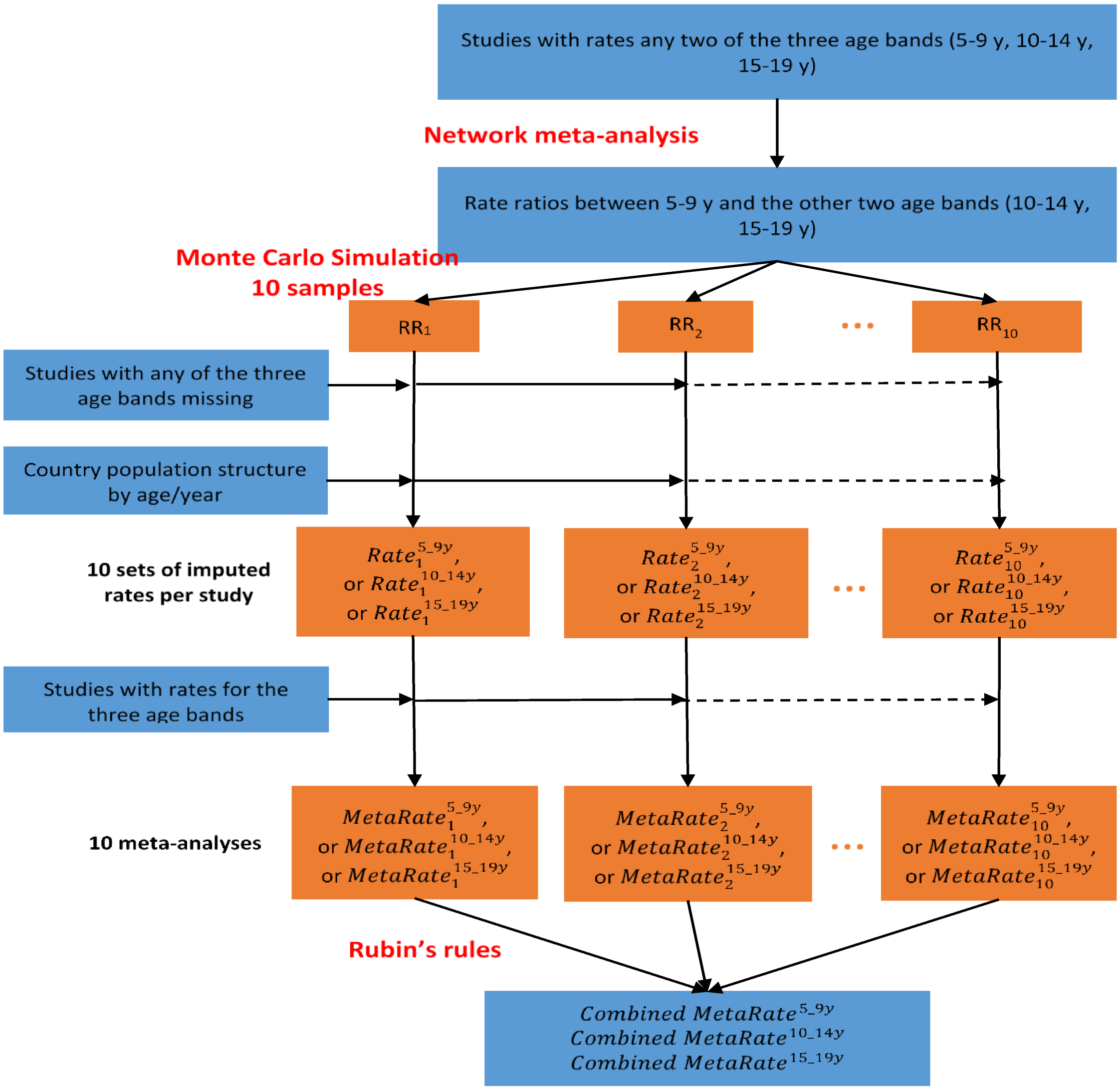


Error! No text of specified style in document.**Figure S2.1. Imputing missing rates for any of 5-9, 10-14, and 15-19 years using the multiple imputation approach.**

**Table S2.1. Estimation of rate ratios for other age bands.**

| Age band | Rate ratio (A)[*] | UNPD country population (B)[3] | Estimated rate ratio VS 5-9 y |
|---|---|---|---|
| 5-9 y | 1.0 | B1 | -- |
| 10-14 y | A2 | B2 | -- |
| 15-19 y | A3 | B3 | -- |
| 5-14 y | | | $\frac{(1*B1+A2*B2)}{(B1+B2)}$ |
| 5-19 y | | | $\frac{(1*B1+A2*B2+A3*B3)}{(B1+B2+B3)}$ |
| Other age bands… | | Corresponding B | … |

**Table S2.2. Comparison of rate ratios from the network meta-analysis and the conventional meta-analysis.**

| Age | Rate ratios from the network meta-analysis | Rate ratios from the conventional meta-analysis |
|---|---|---|
| **ALRI hospital admissions** | | |
| 5-9 y | 1.0 | 1.0 |
| 10-14 y | 0.41 (0.37-0.46) | 0.44 (0.32-0.58) (9 studies) |
| 15-19 y | 0.44 (0.37-0.51) | 0.52 (0.22-1.26) (4 studies) |
| **influenza-associated ALRI hospital admissions** | | |
| 5-9 y | 1.0 | 1.0 |
| 10-14 y | 0.41 (0.27-0.62) | 0.41 (0.26-0.64) (8 studies) |
| 15-19 y | 0.22 (0.10-0.46) | 0.25 (0.20-0.32) (2 studies) |

**Table S2.3 ALRI hospital admission rates and number of hospital admissions without imputation.**

| By World Bank income region | | No imputation[†] | Imputation |
|---|---|---|---|
| **Low** | No. of countries**Error! Bookmark not defined.** | 2 | 2 |
| | Hospital admission rate | 2.2 (1.6-3) | 1.9 (1.4-2.6) |
| | Hospital admissions | 586500 (425600-797100) | 525100 (378800-718500) |
| **Lower middle** | No. of countries**Error! Bookmark not defined.** | 11 | 11 |
| | Hospital admission rate | 3.4 (2.5-4.6) | 2.9 (2.1-4.1) |
| | Hospital admissions | 2991600 (2228900-4044400) | 2608700 (1885700-3630800) |
| **Upper middle** | No. of countries**Error! Bookmark not defined.** | 7 | 7 |
| | Hospital admission rate | 5 (3.3-7.4) | 3.9 (2.6-5.6) |
| | Hospital admissions | 2716100 (1810900-4057000) | 2125400 (1456800-3092900) |
| **High** | No. of countries**Error! Bookmark not defined.** | 21 | 21 |

[*] Estimated from the network meta-analysis.

[†] All data for any age bands between 5 and 19 years were analysed to generate an average rate for 5-19 years.

| | | | |
|---|---|---|---|
| | Hospital admission rate | 1.4 (1.3-1.6) | 1.4 (1.2-1.7) |
| | Hospital admissions | 289800 (262300-323800) | 286800 (246200-340400) |
| **Global** | Hospital admissions | 6584000 (4727700-9222300)‡ | 5546100 (3967500-7782700) |

‡ The slightly higher estimate when imputation was not done could be due to the more frequent report of data for 5-9y, which were higher than older age groups (10-14y).

## Appendix 3 Sensitivity analyses

### 1. ALRI hospital admissions

We excluded the two potential outlier studies with the highest rates (one in Bolivia and the other one in China) in a sensitivity analysis (Table S3.1). After excluding the two studies, we were unable to estimate burden in Bolivia as there were no other studies; the estimate in China after the exclusion was similar to that in the main analysis, with uncertainty ranges overlapping. The estimate of global ALRI hospital admissions remained similar after the exclusion.



Wang X, *et al. BMJ Global Health* 2021; 6:e006014. doi: 10.1136/bmjgh-2021-006014

**Table S3.1 Estimates of ALRI hospital admission rates (per 1,000 children and adolescents per year) and hospital admissions after excluding two potential outlier studies with the highest rates.[*]**

| Country / region | | 5-9 years | 10-14 years | 15-19 years | 5-19 years |
|---|---|---|---|---|---|
| **China** | No. of studies[†] | 9 (9) | 9 (9) | 9 (9) | |
| | Hospital admission rate[‡] | 6.2 (4.8-7.9) | 2.6 (2.1-3.4) | 2.7 (2.1-3.6) | |
| | Hospital admissions | 536300 (418500-679700) | 221700 (173300-280600) | 225600 (172500-291600) | 983500 (764300-1251800) |
| **By World Bank income region** | | | | | |
| Low | No. of studies | 2 | 2 | 2 | 2 |
| | Hospital admission rate | 3 (2.2-4.1) | 1.2 (0.9-1.7) | 1.3 (0.9-1.8) | 1.9 (1.4-2.6) |
| | Hospital admissions | 301200 (218800-409500) | 110000 (79100-150900) | 99600 (70500-138700) | 514000 (370800-703200) |
| Lower middle | No. of studies | 10 | 10 | 10 | 10 |
| | Hospital admission rate | 4.4 (3.1-6.1) | 2 (1.4-2.7) | 2 (1.4-2.9) | 2.8 (2-3.9) |
| | Hospital admissions | 1331800 (960100-1863500) | 586100 (423600-813900) | 579800 (412100-817600) | 2474200 (1778900-3462400) |
| Upper middle | No. of studies | 7 | 7 | 7 | 7 |
| | Hospital admission rate | 5.4 (4.3-6.8) | 2.3 (1.8-2.9) | 2.3 (1.8-3) | 3.4 (2.7-4.3) |
| | Hospital admissions | 1020900 (811800-1284500) | 415100 (324900-528500) | 418700 (324500-538600) | 1847600 (1455600-2342900) |
| High | No. of studies | 21 | 21 | 21 | 21 |
| | Hospital admission rate | 2.3 (2-2.7) | 1 (0.8-1.1) | 1 (0.8-1.2) | 1.4 (1.2-1.7) |
| | Hospital admissions | 154500 (135400-179800) | 64500 (54900-77000) | 68600 (56800-83900) | 286900 (246400-340000) |
| **Global** | Hospital admissions | 2808400 (2126100-3737200) | 1175700 (882600-1570300) | 1166600 (864000-1578700) | 5122800 (3851600-6848500) |

[*]Estimates in countries other than China and Bolivia were not presented in this table as they were the same with those in the main analysis. We were unable to estimate burden in Bolivia as there were no data after excluding the two studies.

[†] The number in parentheses is the number of imputed studies.

[‡] Estimates of hospital admission rates were from meta-analyses.

## 2. ALRI in-hospital deaths

**Table S3.2 Estimates of ALRI in-hospital case-fatality ratios (hCFRs, %) and in-hospital mortality by World Bank income region.***

| | World Bank income region | No. of studies | CFR% | In-hospital mortality |
|---|---|---|---|---|
| **Main analysis – including all CFR data†** | Low & lower middle | 7 | 1.6 (0.7-4.1) | 51100 (18300-126600) |
| | Upper middle | 8 | 1.6 (0.6-4.2) | 32500 (11900-90400) |
| | High | 12 | 0.4 (0.2-0.6) | 1000 (600-1800) |
| | Global | | | 87900 (40300-180600) |
| **Using CFR data from a mixture of 5-14 y and 5-19 y‡** | Low & lower middle | 7 | 1.6 (0.7-4.1) | 51100 (18300-126600) |
| | Upper middle | 5 | 1.7 (0.5-5.2) | 34800 (11100-109900) |
| | High | 11 | 0.4 (0.2-0.7) | 1000 (600-1900) |
| | Global | | | 90900 (41200-197300) |
| **Using CFR data for 5-19 y§** | Low & lower middle | 3 | 2.1 (0.9-5.0) | 64900 (23800-157100) |
| | Upper middle | 3 | 0.2 (0.1-0.6) | 9700 (6600-14200) |
| | High | 6 | 0.5 (0.4-0.8) | 1000 (500-2200) |
| | Global | | | 75100 (33700-169000) |

* CFR estimates were from meta-analyses. In-hospital mortality were estimated by combining CFR estimates and the ALRI hospital admissions for 5-19 years.

† Based on all 27 studies. Of 27 studies, 24 studies reported data for 5-14 years or 5-19 years, the other studies reported data for 5-9 years (2 studies), 6-15 years (1 study), and 5-12 years (1 study).

‡ Based on data from a mixture of 5-14 years and 5-19 years.

§ Based on data for 5-19 years.



Wang X, *et al. BMJ Global Health* 2021; 6:e006014. doi: 10.1136/bmjgh-2021-006014

## 3. Influenza-associated ALRI hospital admissions

**Table S3.3 Estimates of influenza-associated ALRI hospital admissions after excluding two potentially outlier studies with the highest rates.***

| Country / region | | 5-9 years | 10-14 years | 15-19 years | 5-19 years |
|---|---|---|---|---|---|
| **China** | No. of data points | 4 (4) | 4 (4) | 4 (4) | NA |
| | Hospital admission rates (/1,000) | 1 (0.7-1.3) | 0.4 (0.2-0.6) | 0.2 (0.1-0.5) | NA |
| | Hospital admissions | 82700 (61500-109900) | 32600 (19600-53100) | 16000 (5800-42200) | 131300 (86800-205200) |
| **By World Bank income region** | | | | | |
| **Low** | No. of countries | 2 | 2 | 2 | 2 |
| | Hospital admission rates (/1,000) | 0.4 (0.3-0.5) | 0.2 (0.1-0.2) | 0.1 (0-0.2) | 0.2 (0.1-0.3) |
| | Hospital admissions | 38900 (27700-54000) | 14100 (9000-21700) | 5900 (2700-12500) | 59500 (39800-88900) |
| **Lower middle** | No. of countries | 8 | 8 | 8 | 8 |
| | Hospital admission rates (/1,000) | 0.9 (0.4-2.3) | 0.4 (0.1-1.2) | 0.2 (0.1-0.8) | 0.5 (0.2-1.5) |
| | Hospital admissions | 280900 (112500-715700) | 112600 (36200-366200) | 67500 (19100-234000) | 452800 (164100-1297900) |
| **Upper middle** | No. of countries | 3 | 3 | 3 | 3 |
| | Hospital admission rates (/1,000) | 0.9 (0.7-1.2) | 0.4 (0.2-0.6) | 0.2 (0.1-0.5) | 0.5 (0.3-0.8) |
| | Hospital admissions | 172700 (127100-232900) | 68500 (40700-115300) | 33300 (12200-92600) | 273200 (178800-439400) |
| **High** | No. of countries | 13 | 13 | 13 | 13 |
| | Hospital admission rates (/1,000) | 0.2 (0.2-0.3) | 0.1 (0.1-0.2) | 0 (0-0.1) | 0.1 (0.1-0.2) |
| | Hospital admissions | 16200 (11700-23300) | 6900 (4300-11100) | 3400 (1600-6900) | 26400 (17600-41200) |
| **Global** | Hospital admissions | 508800 (278900-1025900) | 202000 (90000-514300) | 110000 (35600-346000) | 811800 (400300-1867400) |

*Estimates in countries other than China and Bolivia were not presented in this table as they were the same with those in the main analysis. We were unable to estimate burden in Bolivia as there were no data after excluding the two studies.

**Table S3.4 Estimates of influenza-associated ALRI hospital admission rates (per 1,000 children and adolescents per year) and hospital admissions after excluding studies with a high risk in case definition.***

| Region | | 5-9 y | 10-14 y | 15-19 y | 5-19 y |
|---|---|---|---|---|---|
| **By World Bank income region** | | | | | |
| **Low** | No. of countries | 2 | 2 | 2 | 2 |
| | Hospital admission rates (/1,000) | 0.4 (0.3-0.5) | 0.2 (0.1-0.2) | 0.1 (0-0.2) | 0.2 (0.1-0.3) |
| | Hospital admissions | 38900 (27700-54000) | 14100 (9000-21700) | 5900 (2700-12500) | 59500 (39800-88900) |
| **Lower middle** | No. of countries | 9 | 9 | 9 | 9 |
| | Hospital admission rates (/1,000) | 1 (0.4-2.4) | 0.4 (0.1-1.3) | 0.2 (0.1-0.9) | 0.5 (0.2-1.5) |
| | Hospital admissions | 295700 (121000-740000) | 118600 (39000-377900) | 70200 (19700-246600) | 475800 (175700-1346000) |
| **Upper middle** | No. of countries | 3 | 3 | 3 | 3 |
| | Hospital admission rates (/1,000) | 1.7 (0.8-3.6) | 0.7 (0.3-1.7) | 0.4 (0.1-1.1) | 0.9 (0.4-2.1) |
| | Hospital admissions | 321800 (147400-679100) | 129100 (53600-301400) | 64500 (21400-191000) | 512900 (221200-1166700) |
| **High** | No. of countries | 11 | 11 | 11 | 11 |
| | Hospital admission rates (/1,000) | 0.3 (0.2-0.3) | 0.1 (0.1-0.2) | 0.1 (0-0.1) | 0.1 (0.1-0.2) |
| | Hospital admissions | 17800 (13800-22900) | 7500 (5000-11200) | 3800 (1900-7500) | 28900 (20500-41500) |
| **Global** | Hospital admissions | 674100 (309900-1496000) | 269200 (106500-712100) | 144300 (45600-457600) | 1077100 (457200-2643100) |

* High risk of bias in case definition: ICD-coded influenza-confirmed respiratory hospital admissions (e.g., ICD-10 J09-10); hospitalised with fever or acute respiratory infection AND lab-confirmed influenza.

**Table S3.5 Estimates of influenza-associated ALRI hospital admission rates (per 1,000 children and adolescents per year) and hospital admissions after excluding studies with unknown testing levels**

| Region | | 5-9 y | 10-14 y | 15-19 y | 5-19 y |
|---|---|---|---|---|---|
| **By World Bank income region** | | | | | |
| **Low** | No. of countries | 1 | 1 | 1 | 1 |
| | Hospital admission rates (/1,000) | 0 (0-0) | 0 (0-0) | 0 (0-0) | 0 (0-0) |
| | Hospital admissions | 3800 (3400-4300) | 1400 (1000-1900) | 600 (300-1200) | 5900 (4800-7400) |
| **Lower middle** | No. of countries | 8 | 8 | 8 | 8 |
| | Hospital admission rates (/1,000) | 1 (0.4-2.5) | 0.4 (0.1-1.3) | 0.3 (0.1-0.9) | 0.6 (0.2-1.6) |
| | Hospital admissions | 305800 (124700-767600) | 122100 (40000-390600) | 72300 (20300-253500) | 490500 (180600-1391000) |
| **Upper middle** | No. of countries | 3 | 3 | 3 | 3 |
| | Hospital admission rates (/1,000) | 1.7 (0.8-3.6) | 0.7 (0.3-1.7) | 0.4 (0.1-1.1) | 0.9 (0.4-2.1) |
| | Hospital admissions | 321800 (147400-679100) | 129100 (53600-301400) | 64500 (21400-191000) | 512900 (221200-1166700) |
| **High** | No. of countries | 6 | 6 | 6 | 6 |
| | Hospital admission rates (/1,000) | 23900 (17600-32100) | 10000 (6500-15300) | 5000 (2400-10000) | 38500 (26200-57000) |
| | Hospital admissions | 0.4 (0.3-0.5) | 0.1 (0.1-0.2) | 0.1 (0-0.1) | 0.2 (0.1-0.3) |
| **Global** | Hospital admissions | 655300 (293100-1483100) | 262700 (101100-709300) | 142400 (44400-455800) | 1047700 (432700-2622200) |

**Table S3.6 Estimates of proportion positives of influenza-associated ALRI and the hospital admissions in children and adolescents aged 5-19 years by World Bank income region.** *

| World Bank income region | No. of studies | Proportion† | Influenza-associated ALRI hospital admissions |
|---|---|---|---|
| **Low & lower middle** | 31 | 19.2 (14.4-25.6) | 594500 (381800-908600) |
| **Upper middle** | 26 | 8 (5.4-11.9) | 169200 (94600-287500) |
| **High** | 13 | 10.9 (6.3-18.7) | 30800 (17600-55400) |
| **Global** | | | 796600 (525900-1177500) |

* Based on all 70 studies. Of 70 studies, three studies for 5-9 years, 50 studies for 5-14 years, and 17 studies for 5-19 years.

† Estimates from meta-analyses.

## 3. Respiratory syncytial virus-associated ALRI hospital admissions

**Table S3.7 Estimates of proportion positives of respiratory syncytial virus-associated ALRI and the hospital admissions in children and adolescents aged 5-19 years by World Bank income region.**

| | World Bank income region | No. of studies | Proportion* | Respiratory syncytial virus-associated ALRI hospital admissions |
|---|---|---|---|---|
| **The main analysis – including all proportion data†** | Low & lower middle | 15 | 4.0 (2.4-6.8) | 125000 (66800-222600) |
| | Upper middle | 27 | 4.5 (2.8-7.2) | 94500 (51100-170200) |
| | High | 12 | 3.9 (1.6-9.2) | 10800 (4800-27600) |
| | Global | | | 231800 (142700-373200) |
| **Using proportion data from a mixture of 5-14 y and 5-19 y‡** | Low & lower middle | 15 | 4 (2.4-6.6) | 123200 (66500-218400) |
| | Upper middle | 26 | 4.3 (2.7-6.9) | 90700 (48900-165000) |
| | High | 10 | 3.4 (1.3-9.3) | 9500 (3800-28100) |
| | Global | | | 227100 (138700-367300) |
| **Using proportion data for 5-19 y§** | Low & lower middle | 6 | 4.5 (1.9-10.9) | 141200 (52000-341200) |
| | Upper middle | 7 | 7.8 (3.2-18.7) | 161800 (65200-410700) |
| | High | 4 | 12.8 (7.1-23.1) | 36200 (19900-68000) |
| | Global | | | 357100 (182800-684300) |

* Estimates from meta-analyses.
† Based on all 54 studies. Of 52 studies, three studies for 5-9 years, 33 studies for 5-14 years, and 17 studies for 5-19 years.
‡ Based on data from a mixture of 5-14 years and 5-19 years.
§ Based on data for 5-19 years.

## Appendix 4 Trend of hospital admission rates of ALRI

Data on ALRI hospital admission rates over four or more consecutive years are plotted in Figure S4.1. The trend of ALRI hospital admission rates varied by geographic locations: a decrease was found in Brazil (annual percent change, -3%), Canada (-3%), France (-3%), and Kenya (-8%); an increase in Denmark (+4%), Netherlands (+4%), and Taiwan, China (+14%). We did not found significant change of ALRI hospital admission rates over time in other locations.

We plotted multi-year studies with influenza-associated ALRI hospital admission rates for four or more consecutive years in Figure S4.2.

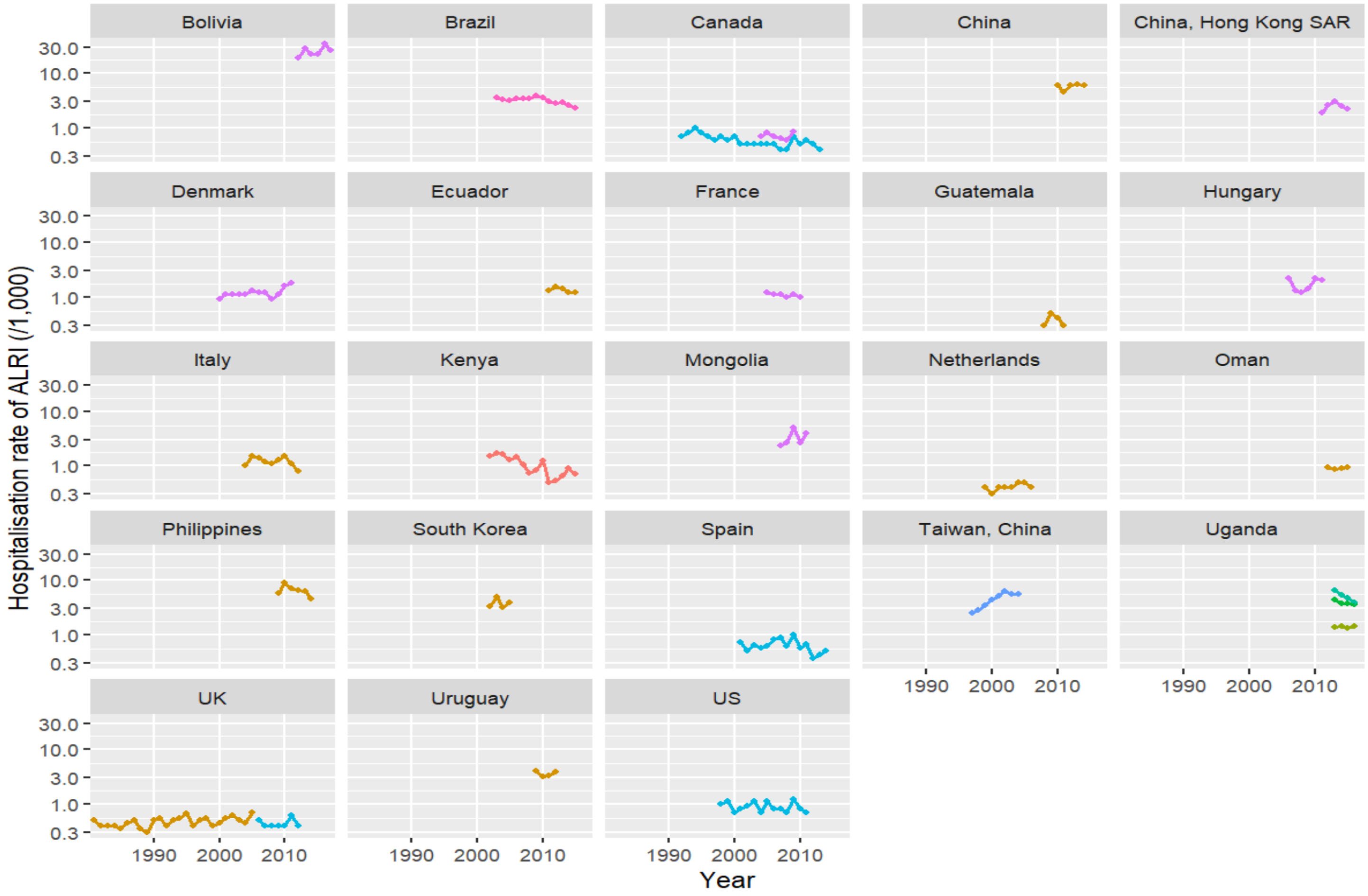


**Figure S4.1 Trend of ALRI hospital admission rates in children and adolescents between 5 and 19 years.**

For each region/country, the points represent the rates for each year, and the lines connecting these points represent the rates over years for one site or one age band. Different colours represent data from different sites or age bands. For example, in Canada the purple line represent the national rates for 5-19 years, and the blue line represent the rates among children and adolescents aged 5-17 years in Ontario, Canada.

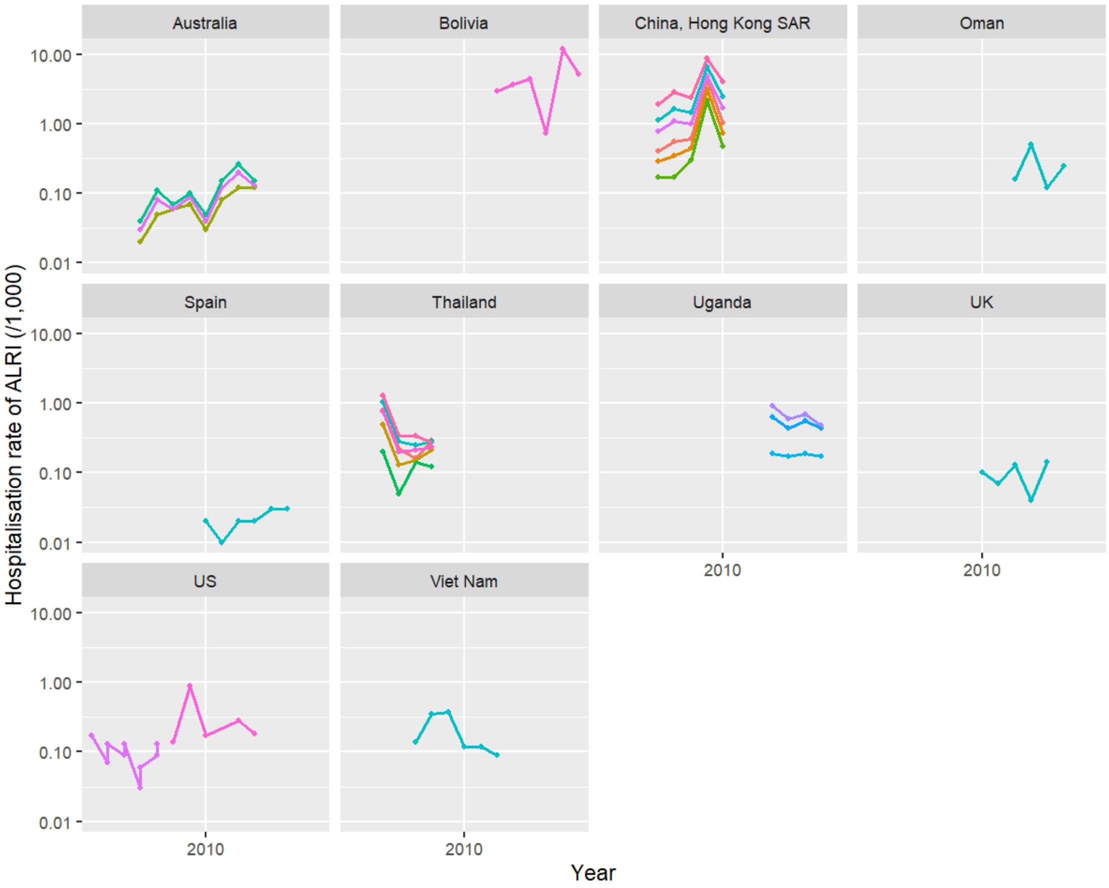


**Figure S4.2 Trend of influenza-associated ALRI hospital admission rates in children and adolescents between 5 and 19 years.**

For each region/country, the points represent the rates for each year, and the lines connecting these points represent the rates over years for one site or one age band. Different colours represent data from different sites or age bands.

**Appendix 5 Details of included studies**

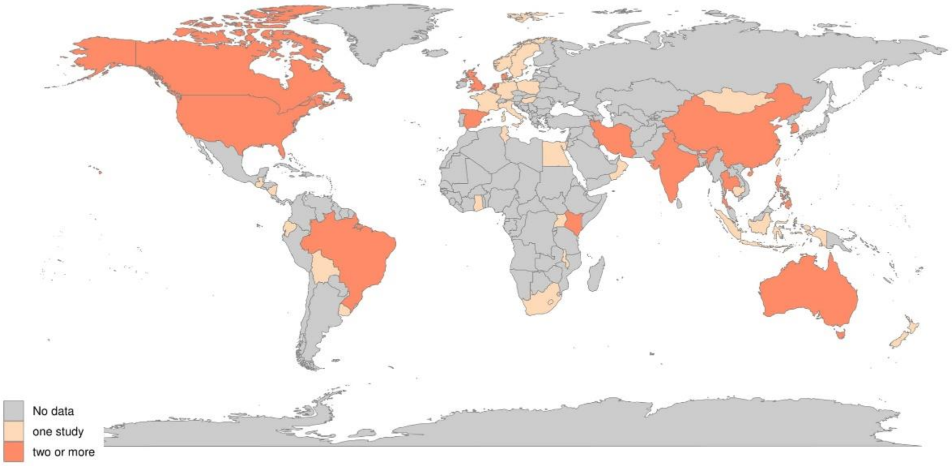


Figure S5.1 Countries of included all-cause ALRI hospital admission rate data

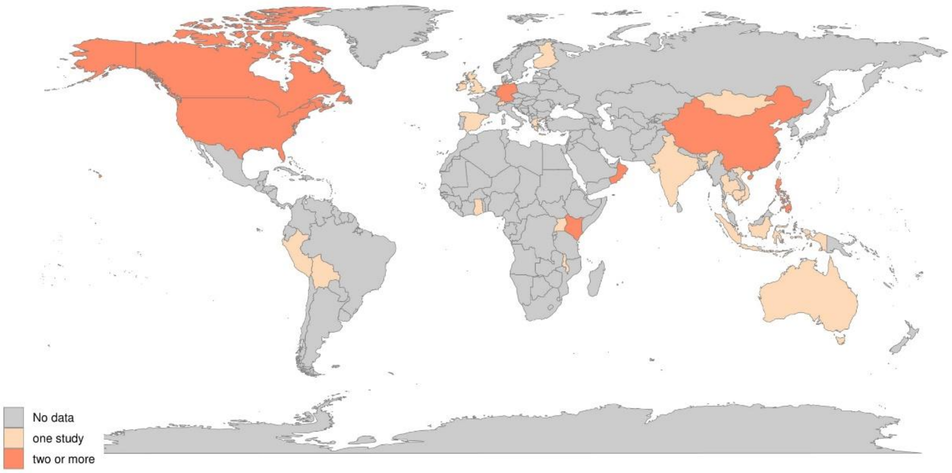


Figure S5.2 Countries of included influenza-associated ALRI hospital admission rate data

**Table S5.1 Details of studies with data on hospital admission rates of ALRI per 1,000 children and adolescents per year.***

| Study ID | Location (reference) | Case definition | 5-9 y | 10-14 y | 15-19 y | 5-14 y | 5-19 y | 5-17 y |
|---|---|---|---|---|---|---|---|---|
| **p1000** | Baguio, Philippines (2010-2011)[7] | ALRI | NA | NA | NA | 7.3 | NA | NA |
| **p1006** | Baguio, Philippines (2012-2014) [8] | ALRI | NA | NA | NA | 5.5 | NA | NA |
| **p1015** | Chrzanów County, Poland (2006-2008) [9] | ALRI | NA | NA | NA | 2.9 | NA | NA |
| **p113** | Canada (2004-2010) [10] | ALRI | 1.3 | 0.5 | 0.4 | 0.9 | 0.7 | NA |
| **p117** | Edmonton, Canada (2000-2002) [11] | CXR-pneumonia | NA | NA | NA | NA | NA | NA |
| **p125** | multisites, Canada (1995-2001) [12] | ARI | NA | NA | NA | 2.6 | NA | NA |
| **p1483** | South Africa (2007-2012) [13] | ARI | NA | NA | NA | NA | 4.3 | NA |
| **p1503** | South Korea (2002-2005) [14] | ALRI | NA | NA | NA | 3.9 | NA | NA |
| **p1505** | South Korea (2007-2014) [15] | ALRI | NA | NA | NA | NA | NA | NA |
| **p1516** | Spain (1995-1998) [16] | ALRI | 1.1 | NA | NA | NA | NA | NA |
| **p1517** | Spain (2001-2014) [17] | ARI | 1.2 | NA | NA | NA | NA | 0.6 |
| **p1522** | Sweden (2005) [18] | ARI | 1.1 | NA | NA | NA | NA | NA |
| **p1532** | Sa Kaeo, Thailand (2002-2003) [19] | ARI | NA | NA | NA | 3.3 | NA | NA |
| **p1541** | Sa Kaeo and Nakhon Phanom, Thailand (2003-2007) [20] | ARI | NA | NA | NA | NA | 2.7 | NA |
| **p1546** | Sa Kaeo and Nakhon Phanom, Thailand (2008-2011) [21] | ARI | NA | NA | NA | NA | 5.6 | NA |
| **p1548** | Sfax, Tunisia (2003-2015) [22] | ALRI | 0.4 | 0.2 | NA | 0.3 | NA | NA |
| **p1566** | Wakiso, Uganda (2013-2016) [23] | ALRI | NA | NA | NA | 2.4 | NA | NA |
| **p1571** | England, UK (2001-2013) [24] | ALRI | 1.6 | 0.7 | NA | 1.1 | NA | NA |
| **p1577** | North East England, UK (2001-2002) [25] | CXR-pneumonia | NA | NA | NA | NA | NA | NA |
| **p1578** | North East England, UK (2008-2009) [26] | CXR-pneumonia | NA | NA | NA | NA | NA | NA |
| **p1583** | Scotland, UK (1981-2005) [27] | ALRI | 0.6 | 0.3 | NA | 0.5 | NA | NA |
| **p1587** | Scotland, UK (2000-2012) [28] | ALRI | NA | NA | NA | NA | NA | 0.4 |
| **p1604** | two municipalities, Uruguay (2009-2012) [29] | ALRI | NA | NA | NA | 3.5 | NA | NA |
| **p1640** | US (1996-2011) [30] | ARI | NA | NA | NA | NA | NA | 0.9 |
| **p165** | Ontario, Canada (1992-2014) [31] | ALRI | NA | NA | NA | NA | NA | 0.6 |
| **p1654** | US (2012-2014) [32] | ALRI | NA | NA | NA | NA | 0.7 | NA |
| **p1871** | Beijing, China (2014-2016) [33] | ARI AND fever | NA | NA | NA | 3 | NA | NA |
| **p226** | Beijing, China (2017-2018) [34] | ARI AND fever | NA | NA | NA | 3.2 | NA | NA |
| **p286** | China, Hong Kong SAR (2005) [35] | ALRI | NA | NA | NA | 3.1 | NA | NA |

* ARI: any respiratory symptoms (e.g., cough, runny nose, sore throat, shortness of breath, or difficulty in breathing) requiring hospital admission. ARI AND fever: fever or history of fever AND any respiratory symptoms requiring hospital admission. ALRI: any of physician-diagnosed pneumonia (and bronchiolitis), ICD-coded pneumonia (and bronchiolitis), or fever or history of fever AND cough or sour throat AND shortness of breath or difficulty breathing. CXR-pneumonia: chest radiograph confirmed pneumonia. ARI OR fever: any respiratory symptoms OR fever requiring hospital admission. y: years.

| Study ID | Location (reference) | Case definition | 5-9 y | 10-14 y | 15-19 y | 5-14 y | 5-19 y | 5-17 y |
|---|---|---|---|---|---|---|---|---|
| **p321** | China, Hong Kong SAR (2011-2015) [36] | ALRI | NA | NA | NA | NA | 2.4 | NA |
| **p355** | Jing Zhou, China (2010-2012) [37] | ARI AND fever | 36.9 | 6.3 | NA | 21 | NA | NA |
| **p416** | Suzhou, China (2010-2014) [38] | ARI | NA | NA | NA | 5.8 | NA | NA |
| **p420** | Taiwan, China (1997-2004)[†] [39] | ALRI | NA | NA | NA | NA | NA | NA |
| **p433** | Denmark (1995-1999) [40] | ALRI | 1.8 | 0.6 | 0.5 | 1.2 | 1 | NA |
| **p440** | Denmark (1997-2011) [41] | ALRI | NA | NA | NA | NA | 1.2 | NA |
| **p447** | Ecuador (2011-2015) [42] | ARI | NA | NA | NA | 1.3 | NA | NA |
| **p45** | Mornington, Australia (2007-2011) [43] | ALRI | NA | NA | NA | 2.6 | NA | NA |
| **p454** | Damanhour, Egypt (2009-2012) [44] | ARI AND fever | NA | NA | NA | NA | 0.9 | NA |
| **p509** | Rhône-Alpes, France (2005-2010) [45] | ALRI | NA | NA | NA | NA | 1.1 | NA |
| **p524** | Kiel, Germany (1996-1999) [46] | ALRI | NA | NA | NA | NA | NA | NA |
| **p532** | Shai-Osudoku and Ningo-Prampram, Ghana (2013-2015) [47] | ARI AND fever | NA | NA | NA | 2.3 | NA | NA |
| **p55** | Western Australia (1996-2012) [48] | ALRI | 2 | NA | NA | NA | NA | NA |
| **p557** | Santa Rosa and Quetzaltenango, Guatemala (2007-2011) [49] | ARI | NA | NA | NA | 0.4 | NA | NA |
| **p566** | Hungary (2006-2011) [50] | ALRI | NA | NA | NA | NA | 1.7 | NA |
| **p567** | Ballabgarh, India (2010-2012) [51] | ARI | NA | NA | NA | 4.3 | NA | NA |
| **p572** | Ballabgarh, India (2012-2014) [52] | ALRI | 2.1 | NA | NA | NA | NA | NA |
| **p62** | Brazil (2003-2007 for hCFR; 2003/2004 for admission) [53] | ALRI | 3.4 | 1.3 | 1.2 | 2.3 | 2 | NA |
| **p630** | Wonosari, Indonesia (2013-2016) [54] | ARI AND fever | NA | NA | NA | 1.5 | NA | NA |
| **p633** | Iran (2015) [55] | ARI AND fever | NA | NA | NA | 0.3 | NA | NA |
| **p646** | multisites, Iran (2012-2013) [56] | ARI AND fever | NA | NA | NA | 1.3 | NA | NA |
| **p669** | Negev, Israel (1989-1991) [57] | ALRI | NA | NA | NA | 1.4 | NA | NA |
| **p672** | Veneto, Italy (2004-2012) [58] | ALRI | NA | NA | NA | 1.2 | NA | NA |
| **p689** | Bondo, Kenya (2001-2003) [59] | ALRI | 0.8 | 0.8 | 2 | 0.8 | 1.1 | NA |
| **p693** | Bondo, Kenya (2007-2009) [60] | ALRI | NA | NA | NA | NA | 2.9 | NA |
| **p704** | Dadaab, Kenya (2010-2012) [61] | ALRI | NA | NA | NA | 1.5 | NA | NA |
| **p705** | Karemo, Kenya (2009-2012) [62] | ARI | NA | NA | NA | NA | NA | 2 |
| **p706** | Kenya (2012-2014) [63] | ARI AND fever | NA | NA | NA | 0.7 | NA | NA |
| **p73** | Brazil (2005-2015) [64] | ALRI | 3.1 | NA | NA | NA | NA | 1.8 |
| **p731** | Kilifi, Kenya (2002-2015) [65] | ALRI | NA | NA | NA | NA | NA | NA |
| **p748** | Blantyre, Malawi (2011-2014) [66] | ALRI | 0.1 | 0.1 | NA | 0.1 | NA | NA |
| **p774** | multisites, Mongolia (2007-2012) [67] | ALRI | 4.8 | NA | NA | NA | 3.3 | NA |
| **p877** | Netherlands (2001-2007) [68] | ARI | NA | NA | NA | 0.4 | NA | NA |

[†] Data were reported for other age bands, so are not presented in this table.

| Study ID | Location (reference) | Case definition | 5-9 y | 10-14 y | 15-19 y | 5-14 y | 5-19 y | 5-17 y |
|---|---|---|---|---|---|---|---|---|
| **p882** | Netherlands (2008-2011) ‡ [69] | ALRI | NA | NA | NA | NA | NA | NA |
| **p939** | Auckland, New Zealand (1993-1996) [70] | ARI | NA | NA | NA | 1.4 | NA | NA |
| **p94** | La Paz, Bolivia (2012-2017) [71] | ARI AND fever | NA | NA | NA | NA | 25.4 | NA |
| **p945** | Leo´n, Nicaragua (2008-2015) [72] | CXR-pneumonia | NA | NA | NA | 0.8 | NA | NA |
| **p954** | Norway (2008-2009) [73] | ALRI | NA | NA | NA | NA | 1.2 | NA |
| **p983** | Oman (2012-2015) [74] | ARI | NA | NA | NA | 0.9 | NA | NA |
| **p99** | Svay Rieng, Cambodia (2015-2016) § [75] | ALRI | NA | NA | NA | NA | NA | NA |

‡ Data were reported for other age bands, so are not presented in this table.
§ Data were reported for other age bands, so are not presented in this table.

**Table S5.2 Details of studies with data on incidence rates of ALRI (per 1,000 per year) ***

| Study ID | Location (reference) | Case definition | Age | Rate (/1,000) |
|---|---|---|---|---|
| **P1595** | UK (1991-2003)[76] | ALRI | 5-14 y | 0.8 |
| **p1597** | UK (1995; 2000) [77] | ALRI | 5-16 y | 44.4 |
| **p1600** | UK (1997-2012) [78] | ALRI | 5-12 y | 4.6 |
| **p1638** | US (1994-2007) [79] | ALRI | 6-18 y | 10.8 |
| **p1657** | US (2008-2014) [80] | ALRI | 5-17 y | 13.5 |
| **p1706** | Washington State, US (1998-2004) [81] | ALRI | 5-17 y | 11.7 |
| **p572** | Ballabgarh, India (2012-2014) [52] | ALRI | 5-9 y | 30.2 |
| **p839** | Netherlands (1995-2001) [82] | ALRI (13% of influenza cases) | 5-17 y | 18.7 |
| **p877** | Netherlands (2001-2007) [68] | ALRI | 5-14 y | 4.6 |

* ARI: any respiratory symptoms (e.g., cough, runny nose, sore throat, shortness of breath, or difficulty in breathing) requiring hospital admission. ARI AND fever: fever or history of fever AND any respiratory symptoms requiring hospital admission. ALRI: any of physician-diagnosed pneumonia (and bronchiolitis), ICD-coded pneumonia (and bronchiolitis), or fever or history of fever AND cough or sour throat AND shortness of breath or difficulty breathing. CXR-pneumonia: chest radiograph confirmed pneumonia. y: years.

**Table S5.3 Details of studies with data on in-hospital case-fatality ratios (hCFRs) of ALRI.** [*]

| Study ID | Location (reference) | Case definition | Cases for 5-9 y | hCFR for 5-9 y (%) | Cases for 5-14 y | hCFR for 5-14 y (%) | Cases for 5-19 y | hCFR for 5-19 y (%) |
|---|---|---|---|---|---|---|---|---|
| **p1015** | Chrzanów County, Poland (2006-2008) [9] | ALRI | NA | NA | 269 | 0 | NA | NA |
| **p1071** | multisites, South Africa (2009-2012)[83] | ALRI | NA | NA | 577 | 2.1 | NA | NA |
| **p113** | Canada (2004-2010) [10] | ALRI | 10071 | 0.3 | 13274 | 0.5 | 17372 | 0.8 |
| **p1505** | South Korea (2007-2014)[†] [15] | ALRI | NA | NA | NA | NA | NA | NA |
| **p1513** | Spain (1995-1996) [84] | ALRI | 4303 | 0.3 | NA | NA | NA | NA |
| **p1527** | Sa Kaeo (2002-2003) [85] | CXR-pneumonia | 34 | 11.8 | NA | NA | NA | NA |
| **p1541** | Sa Kaeo and Nakhon Phanom, Thailand (2003-2007) [20] | ARI | NA | NA | NA | NA | 1069 | 0.4 |
| **p1548** | Sfax, Tunisia (2003-2015) [22] | ALRI | 369 | 0.3 | 562 | 0.9 | NA | NA |
| **p1565** | multisites, Uganda (2010-2015) [86] | ARI | NA | NA | 139 | 1.4 | NA | NA |
| **p1568** | England, UK (2000-2007) [87] | ARI | NA | NA | 52523 | 0.1 | NA | NA |
| **p1602** | Paysandú and Salto, Uruguay (2001-2004) [88] | ALRI | NA | NA | 361 | 1.4 | NA | NA |
| **p1654** | US (2012-2014) [32] | ALRI | NA | NA | NA | NA | 68483 | 0.7 |
| **p246** | Guangzhou, China (2009-2012) [‡] [89] | ALRI | NA | NA | NA | NA | NA | NA |
| **p321** | China, Hong Kong SAR (2011-2015) [36] | ALRI | NA | NA | NA | NA | 11337 | 0.9 |
| **p420** | Taiwan, China (1997-2004) [39] | ALRI | 2562 | 0.1 | 3015 | 0.1 | NA | NA |
| **p440** | Denmark (1997-2011) [41] | ALRI | NA | NA | NA | NA | 11725 | 0.4 |
| **p466** | multisites, Egypt (2007-2014) [90] | ARI AND fever | 693 | 0.7 | 1049 | 0.8 | 1366 | 1 |
| **p566** | Hungary (2006-2011) [50] | ALRI | NA | NA | NA | NA | 16473 | 0.5 |
| **p62** | Brazil (2003-2007 for hCFR; 2003/2004 for Hos) [53] | ALRI | 293030 | 0.3 | 405175 | 0.3 | 512235 | 0.5 |
| **p633** | Iran (2015) [55] | ARI AND fever | NA | NA | 3329 | 3 | NA | NA |
| **p689** | Bondo, Kenya (2001-2003) [59] | ALRI | 85 | 5.9 | 135 | 5.2 | 248 | 6 |
| **p739** | multisites, Kenya (2014-2018) [91] | ALRI | 1462 | 6.4 | 1825 | 7.9 | NA | NA |

[*] ARI: any respiratory symptoms (e.g., cough, runny nose, sore throat, shortness of breath, or difficulty in breathing) requiring hospital admission. ARI AND fever: fever or history of fever AND any respiratory symptoms requiring hospital admission. ALRI: any of physician-diagnosed pneumonia (and bronchiolitis), ICD-coded pneumonia (and bronchiolitis), or fever or history of fever AND cough or sour throat AND shortness of breath or difficulty breathing. CXR-pneumonia: chest radiograph confirmed pneumonia. y: years.

[†] Data were reported for other age bands, so are not presented in this table.

[‡] Data were reported for other age bands, so are not presented in this table.

| Study ID | Location (reference) | Case definition | Cases for 5-9 y | hCFR for 5-9 y (%) | Cases for 5-14 y | hCFR for 5-14 y (%) | Cases for 5-19 y | hCFR for 5-19 y (%) |
|---|---|---|---|---|---|---|---|---|
| **p780** | Maputo, Mozambique (2014-2016) [92] | ARI AND fever | NA | NA | 294 | 0 | NA | NA |
| **p794** | eight African countries (2009-2012) [93] | ARI AND fever | NA | NA | NA | NA | NA | NA |
| **p90** | Rio de Janeiro, Brazil (1996-2011) [§] [94] | ALRI | NA | NA | NA | NA | NA | NA |
| **p983** | Oman (2012-2015) [74] | ARI | NA | NA | 1837 | 0.8 | NA | NA |
| **p992** | Asuncion, Paraguay (2004-2013) [95] | CXR-pneumonia | NA | NA | 165 | 10.9 | NA | NA |

[§] Data were reported for other age bands, so are not presented in this table.

**Table S5.4 Details of studies with data on hospital admission rates of influenza-associated ALRI (per 1,000 children and adolescents per year).** *†

| Study ID | Location (reference) | Case definition | Specimen and diagnostic test | 5-9 y | 10-14 y | 15-19 y | 5-14 y | 5-19 y | 5-17 y |
|---|---|---|---|---|---|---|---|---|---|
| **p1000** | Baguio, Philippines (2010-2011) [7] | ALRI | NPS and OPS; PCR | NA | NA | NA | 1.5 | NA | NA |
| **p1006** | Baguio, Philippines (2012-2014) [8] | ALRI | NPS and OPS; PCR | NA | NA | NA | 1.5 | NA | NA |
| **p105** | Canada (2003-2008) [96] | ICD-coded lab-confirmed influenza | NA; NA | 0.1 | 0 | 0 | 0 | 0 | NA |
| **p1070** | Singapore (2013-2014) [97] | ARI AND fever | NS; DFA | 1.2 | NA | NA | NA | 0.5 | NA |
| **p108** | Canada (2003-2007; 2010-2014)‡ [98] | ARI | NA; NA | NA | NA | NA | NA | NA | NA |
| **p1519** | Spain (2010-2016) [99] | ALRI | NA; NA | NA | NA | NA | 0 | NA | NA |
| **p1525** | Basel, Switzerland (2001-2002) § [100] | ARI | NPS; PCR and rapid antigen test | NA | NA | NA | NA | NA | NA |
| **p1545** | Sa Kaeo and Nakhon Phanom, Thailand (2005-2008) [101] | ARI | NPS; mainly PCR (and culture) | 0.6 | 0.4 | 0.1 | 0.5 | 0.3 | NA |
| **p1566** | Wakiso, Uganda (2013-2016) [23] | ALRI | NPS and OPS; PCR | NA | NA | NA | 0.3 | NA | NA |
| **p1574** | England, UK (2010-2015) [102] | ARI AND fever | NA; NA | NA | NA | NA | 0.1 | NA | NA |
| **p1610** | Colorado, US (2004-2008) [103] | ARI | NA; PCR, DFA, and rapid test | NA | NA | NA | NA | NA | 0.1 |
| **p1612** | Memphis, Nashville, Salt Lake City, US (2010-2012) [104] | CXR-pneumonia | NPS and OPS and serum; PCR and serology | 0.1 | NA | NA | NA | NA | 0.1 |
| **p1618** | US (1996-1998) ** [105] | ALRI | mostly NPS; PCR, culture and EIA (enzyme immunoassay) | NA | NA | NA | NA | NA | NA |
| **p1625** | Philadelphia, US (2000-2004) [106] | ARI | Nasal aspirate; culture, DFA, and solid-phase immunoassay (SPIA) | NA | NA | NA | NA | NA | 0.2 |
| **p1637** | Salt Lake County, Utah, US (2001-2004) [107] | ARI | NPA; culture and DFA | NA | NA | NA | 0.1 | NA | NA |

* ARI: any respiratory symptoms (e.g., cough, runny nose, sore throat, shortness of breath, or difficulty in breathing) requiring hospital admission. ARI AND fever: fever or history of fever AND any respiratory symptoms requiring hospital admission. ALRI: any of physician-diagnosed pneumonia (and bronchiolitis), ICD-coded pneumonia (and bronchiolitis), or fever or history of fever AND cough or sour throat AND shortness of breath or difficulty breathing. CXR-pneumonia: chest radiograph confirmed pneumonia. y: years.

† NPS: nasopharyngeal swab. OPS: oropharyngeal swab. PCR: polymerase chain reaction. NS: nasal swab. DFA: direct fluorescent antibody test. EIA: enzyme immunoassay. SPIA: solid-phase immunoassay. TS: throat swab. NPA: nasopharyngeal aspirate. NPW: nasopharyngeal wash.

‡ Data were reported for other age bands, so are not presented in this table.

§ Data were reported for other age bands, so are not presented in this table.

** Data were reported for other age bands, so are not presented in this table.

| Study ID | Location (reference) | Case definition | Specimen and diagnostic test | 5-9 y | 10-14 y | 15-19 y | 5-14 y | 5-19 y | 5-17 y |
|---|---|---|---|---|---|---|---|---|---|
| **p1656** | US (2003-2008) [108] | ARI | NA; PCR (sensitivity adjusted) | NA | NA | NA | NA | NA | 0.1 |
| **p1668** | US (2008-2011) [109] | ARI | NA; PCR (sensitivity adjusted) | NA | NA | NA | NA | 0.2 | NA |
| **p1671** | US (2012-2013) [110] | ARI | NA; PCR (sensitivity adjusted) | NA | NA | NA | NA | 0.3 | NA |
| **p1672** | US (2013-2014) [111] | ARI | NA; PCR (sensitivity adjusted) | NA | NA | NA | NA | 0.2 | NA |
| **p1686** | US (2017-2018) [112] | ARI | NA; PCR (sensitivity adjusted) | NA | NA | NA | NA | NA | 0.4 |
| **p1690** | US (2018-2019) [113] | ARI | NA; PCR (sensitivity adjusted) | NA | NA | NA | NA | NA | 0.4 |
| **p1700** | Utah, US (2016-2017) [114] | ARI | NA; PCR (sensitivity adjusted) | NA | NA | NA | NA | NA | 0.3 |
| **p1735** | Nha Trang, Viet Nam (2007-2012) [115] | ALRI | NPS; PCR | NA | NA | NA | 0.2 | NA | NA |
| **p1871** | Beijing, China (2014-2016) [33] | ARI AND fever | TS; PCR | NA | NA | NA | 0.6 | NA | NA |
| **p226** | Beijing, China (2017-2018) [34] | ARI AND fever | pharyngeal swab; PCR | NA | NA | NA | 0.8 | NA | NA |
| **p328** | China, Hong Kong SAR (2003-2006) [116] | ARI AND fever | NPA; DFA and culture | 2.7 | 0.5 | NA | 1.5 | NA | 1.3 |
| **p330** | China, Hong Kong SAR (2005-2011) [117] | ICD-coded lab-confirmed influenza | NPA; Immunofluorescence and culture | 2.7 | 0.6 | NA | 1.6 | NA | 1.1 |
| **p355** | Jing Zhou, China (2010-2012) [37] | ARI AND fever | NPA; PCR and others | 6.8 | 1 | NA | 3.8 | NA | NA |
| **p39** | Australia (2006-2015) [118] | ICD-coded lab-confirmed influenza | NA; PCR and others | NA | NA | NA | NA | NA | 0.1 |
| **p491** | Turku, Finland (1988-2004) [119] | ARI | NPA; time-resolved fluoroimmunoassay and rapid influenza test | 0.2 | 0.1 | NA | 0.2 | NA | NA |
| **p520** | Germany (2004-2010) [120] | ICD-coded lab-confirmed influenza | NA; NA | NA | NA | NA | 0.1 | NA | NA |
| **p525** | Kiel, Germany (1996-2001) [††] [121] | ALRI | NPA; PCR | NA | NA | NA | NA | NA | NA |
| **p532** | Shai-Osudoku and Ningo-Prampram, Ghana (2013-2015) [47] | ARI AND fever | NPS and OPS; PCR | NA | NA | NA | 0.3 | NA | NA |
| **p536** | Athens, Greece (2002-2005) [122] | ARI OR fever | NPA; PCR | 1 | NA | NA | NA | NA | NA |
| **p567** | Ballabgarh, India (2010-2012) [51] | ARI | NS and TS; PCR | NA | NA | NA | 1.5 | NA | NA |
| **p630** | Wonosari, Indonesia (2013-2016) [54] | ARI AND fever | NA; PCR | NA | NA | NA | 0.3 | NA | NA |

[††] Data were reported for other age bands, so are not presented in this table.

| Study ID | Location (reference) | Case definition | Specimen and diagnostic test | 5-9 y | 10-14 y | 15-19 y | 5-14 y | 5-19 y | 5-17 y |
|---|---|---|---|---|---|---|---|---|---|
| **p655** | Ireland (2010-2011) [123] | ARI AND fever | NA; PCR | 1.7 | 1.3 | NA | 1.5 | NA | NA |
| **p693** | Bondo, Kenya (2007-2009) [60] | ALRI | NPS and OPS; PCR | NA | NA | NA | NA | 0.2 | NA |
| **p704** | Dadaab, Kenya (2010-2012) [61] | ALRI | NPS and OPS; PCR | NA | NA | NA | 0.7 | NA | NA |
| **p705** | Karemo, Kenya (2009-2012) [62] | ARI | NPS and OPS; PCR | NA | NA | NA | NA | NA | 0.2 |
| **p738** | Kilifi, Kenya (2007-2010) [‡‡] [124] | ALRI | NPW or NPA; PCR | NA | NA | NA | NA | NA | NA |
| **p748** | Blantyre, Malawi (2011-2014) [66] | ALRI | NPA; PCR | NA | NA | NA | 0 | NA | NA |
| **p774** | multisites, Mongolia (2007-2012) [67] | ALRI | NPS; PCR and culture | 0.9 | NA | NA | NA | 1 | NA |
| **p94** | La Paz, Bolivia (2012-2017) [71] | ARI AND fever | NA; PCR (flu) and IFA (respiratory syncytial virus) | NA | NA | NA | NA | 4.8 | NA |
| **p963** | Sohar, Oman (2010-2013) [125] | ARI | NPS and OPS; PCR | NA | NA | NA | 0 | NA | NA |
| **p983** | Oman (2012-2015) [74] | ARI | NA; PCR | NA | NA | NA | 0.3 | NA | NA |
| **p99** | Svay Rieng, Cambodia (2015-2016) [§§] [75] | ALRI | NPA; PCR | NA | NA | NA | NA | NA | NA |
| **p995** | multisites, Peru (2009-2015) [126] | ARI AND fever | NPS and OPS; PCR | NA | NA | NA | NA | NA | 0.2 |

‡‡ Data were reported for other age bands, so are not presented in this table.
§§ Data were reported for other age bands, so are not presented in this table.

**Table S5.5 Details of all studies with data on proportion positives of influenza in hospitalised ALRI.** [*†‡]

| Study ID | Location (reference) | Case definition | Specimen and test | ALRI cases for 5-9 y | Proportion for 5-9 y | ALRI cases for 5-14 y | Proportion for 5-14 y | ALRI cases for 5-19 y | Proportion for 5-19 y |
|---|---|---|---|---|---|---|---|---|---|
| **p1000** | Baguio, Philippines (2010-2011)[127] | ALRI | NPS and OPS; PCR | NA | NA | 196 | 14.8 | NA | NA |
| **p1006** | Baguio, Philippines (2012-2014)[8] | ALRI | NPS and OPS; PCR | NA | NA | 268 | 20.1 | NA | NA |
| **p101** | Yaounde, Cameroon (2011-2013) [128] | ARI AND fever | NPS; PCR | NA | NA | 40 | 10 | NA | NA |
| **p1018** | Riyadh, Saudi Arabia (2005-2010) [129] | ALRI | NPA; DFA | NA | NA | NA | NA | 50 | 0 |
| **p1071** | multisites, South Africa (2009-2012) [83] | ALRI | NPS and TS; PCR | NA | NA | 560 | 11.4 | NA | NA |
| **p1488** | Seongnam, South Korea (2006-2016) [130] | ALRI | Nasal aspirate; PCR | 1019 | 11.5 | NA | NA | 1723 | 11.2 |
| **p1489** | Seoul, South Korea (1996-1998) [131] | ALRI | NPA; Culture | 100 | 10 | 250 | 5.2 | NA | NA |
| **p1492** | Seoul, South Korea (2006-2007) [132] | ALRI | NP aspiration; PCR | 63 | 9.5 | 75 | 8 | NA | NA |
| **p1520** | multisies, Spain (2011-2013) [133] | ALRI | NP specimen; PCR | NA | NA | 40 | 25 | NA | NA |
| **p1554** | Bursa, Turkey (2015-2018) [134] | ARI | NPS or BAL (bronchoalveolar lavage); PCR | NA | NA | NA | NA | 84 | 22.6 |
| **p1557** | Istanbul, Turkey (2010-2011) [135] | ARI | NPA; PCR | 11 | 18.2 | NA | NA | NA | NA |
| **p1565** | multisites, Uganda (2010-2015) [86] | ARI | NPS and OPS; PCR | NA | NA | 404 | 13.9 | NA | NA |
| **P1566** | multisites, Uganda (2013-2016) | ALRI | NPS and OPS; PCR | NA | NA | 8912 | 13.0 | NA | NA |
| **p1612** | Memphis, Nashville, Salt Lake City, US (2010-2012)[136] | CXR-pneumonia | NPS and OPS and serum; PCR and serology | 408 | 9.1 | NA | NA | 683 | 9.8 |

[*] ARI: any respiratory symptoms (e.g., cough, runny nose, sore throat, shortness of breath, or difficulty in breathing) requiring hospital admission. ARI AND fever: fever or history of fever AND any respiratory symptoms requiring hospital admission. ALRI: any of physician-diagnosed pneumonia (and bronchiolitis), ICD-coded pneumonia (and bronchiolitis), or fever or history of fever AND cough or sour throat AND shortness of breath or difficulty breathing. CXR-pneumonia: chest radiograph confirmed pneumonia. y: years.

[†] NP: nasopharyngeal. NPS: nasopharyngeal swab. OPS: oropharyngeal swab. PCR: polymerase chain reaction. NS: nasal swab. DFA: direct fluorescent antibody test. EIA: enzyme immunoassay. SPIA: solid-phase immunoassay. TS: throat swab. NPA: nasopharyngeal aspirate. NPW: nasopharyngeal wash. BAL: bronchoalveolar lavage.

[‡] When data were reported for other age bands, data were grouped into the closest one of the above age bands. For example, 5-11 years were re-classified as 5-9 years; 5-15 years and 6-16 years were re-classified as 5-14 years; 5-17 years, 5-18 years, 6-17 years, 7-17 years, and 6-18 years were re-classified as 5-19 years.

| Study ID | Location (reference) | Case definition | Specimen and test | ALRI cases for 5-9 y | Proportion for 5-9 y | ALRI cases for 5-14 y | Proportion for 5-14 y | ALRI cases for 5-19 y | Proportion for 5-19 y |
|---|---|---|---|---|---|---|---|---|---|
| **p1733** | Ho Chi Minh, Vietnam (2004-2008) [137] | ARI | NS, TS, and NPA; PCR | NA | NA | 13 | 30.8 | NA | NA |
| **p1739** | Vietnam (2011-2014) [138] | ALRI | TS and tracheal aspirate; PCR | NA | NA | NA | NA | 156 | 49.0 |
| **p1870** | Cairo, Egypt (2010-2014) [139] | ALRI | NPS and OPS; PCR | NA | NA | NA | NA | 191 | 3.7 |
| **P1871** | Beijing, China (2014-2016) [33] | ARI AND fever | TS; PCR | NA | NA | 12 | 50.0 | NA | NA |
| **p1873** | Lanzhou, China (2004-2005) [140] | CXR-pneumonia | NPA; DFA | NA | NA | 133 | 12.8 | NA | NA |
| **p1901** | Chengdu, China (2007) [141] | ALRI | NP secretion; DFA | NA | NA | 121 | 9.1 | NA | NA |
| **p1931** | Changsha, China (2015) [142] | ARI | NP secretion; DFA | NA | NA | 285 | 1.1 | NA | NA |
| **p216** | Beijing and Shandong, China (2012-2015) [143] | ALRI | NPS, NPA and sputum; PCR | NA | NA | 850 | 5.2 | NA | NA |
| **p221** | Beijing, China (2007-2010) [144] | ALRI | NP secretion; PCR | NA | NA | 358 | 5.9 | NA | NA |
| **p226** | Beijing, China (2017-2018)[34] | ARI AND fever | pharyngeal swab; PCR | NA | NA | 64 | 23.4 | NA | NA |
| **p241** | Beijing, China (2011-2012) [145] | ALRI | Tracheal aspirate; PCR | NA | NA | NA | NA | 93 | 5.4 |
| **p264** | Changsha, China (2007-2008) [146] | ALRI | NPA; PCR | NA | NA | 42 | 7.1 | NA | NA |
| **p311** | Hebei, China (2007-2008) [147] | ALRI | TS; DFA | NA | NA | 361 | 7.5 | NA | NA |
| **p331** | China, Hong Kong SAR (2009-2013) [148] | ARI AND fever | NA; DFA, culture, PCR | NA | NA | NA | NA | 1037 | 17.9 |
| **p367** | Lanzhou,China (2006-2009) [149] | ALRI | NA; PCR | NA | NA | 67 | 7.5 | NA | NA |
| **p376** | multisites,China (2009-2013) [150] | ALRI | NS or aspirate, sputum, BAL or lung puncture; PCR | 2618 | 8.5 | 3510 | 7.9 | NA | NA |
| **p386** | Shanghai, China (2003-2006) [151] | ALRI | NPA; DFA | NA | NA | 974 | 0.8 | NA | NA |
| **p387** | Shanghai,China (2013-2015) [152] | ALRI | NP secretion; DFA | NA | NA | NA | NA | 1164 | 1.8 |
| **p388** | Shanghai, China (2016-2017) [153] | ALRI | NPS or sputum specimen; PCR | NA | NA | 106 | 17.9 | NA | NA |
| **p396** | Shantou, Shenzhen and Jieyang, China (2007-2011) [154] | ARI | NPS or sputum specimen; PCR | NA | NA | 112 | 23.2 | NA | NA |
| **p399** | Shantou, Shenzhen and JIeyang, China (2006-2008) [155] | ALRI | PCR; PCR | NA | NA | 30 | 6.7 | NA | NA |
| **p400** | nine cities, North China (2015) [156] | ALRI | NP secretion; PCR | NA | NA | 331 | 8.2 | NA | NA |
| **p404** | Shenzhen, China (2012-2015) [157] | ARI AND fever | NS; DFA | NA | NA | 1568 | 1 | NA | NA |
| **p421** | Tianjin, China (2015-2016) [158] | ARI AND fever | TS; PCR | NA | NA | 62 | 4.8 | NA | NA |



Wang X, *et al. BMJ Global Health* 2021; 6:e006014. doi: 10.1136/bmjgh-2021-006014

| Study ID | Location (reference) | Case definition | Specimen and test | ALRI cases for 5-9 y | Proportion for 5-9 y | ALRI cases for 5-14 y | Proportion for 5-14 y | ALRI cases for 5-19 y | Proportion for 5-19 y |
|---|---|---|---|---|---|---|---|---|---|
| **p427** | Zhejiang, China (2011-2015) [159] | ALRI | NPS and TS; PCR | NA | NA | 501 | 10 | NA | NA |
| **p428** | multisites, DR Congo (2015) [160] | ARI AND fever | TS and NS; PCR | 266 | 10.2 | NA | NA | 490 | 8.2 |
| **p452** | Damanhour, Egypt (2013) [161] | ARI AND fever | NPS and OPS; PCR | NA | NA | 146 | 26 | NA | NA |
| **p466** | multisites, Egypt (2007-2014) [90] | ARI AND fever | OPS and NPS; PCR | 796 | 19 | NA | NA | 1661 | 18.8 |
| **p476** | multisites, Egypt (2012-2015) [162] | ARI AND fever | NPS and OPS; PCR | NA | NA | 980 | 16.2 | NA | NA |
| **p52** | Perth, Australia (2000-2005) [163] | ALRI | NPA; PCR | 102 | 1 | NA | NA | NA | NA |
| **p523** | Kiel, Germany (1995-1999) [164] | ALRI | NPA; PCR | NA | NA | 107 | 23.4 | NA | NA |
| **p532** | Shai-Osudoku and Ningo-Prampram, Ghana (2013-2015)[47] | ARI AND fever | NPS and OPS; PCR | NA | NA | 108 | 13 | NA | NA |
| **p540** | Athens, Greece (pre 2003) [165] | CXR-pneumonia | NPW; PCR | NA | NA | 75 | 6.7 | NA | NA |
| **p612** | Odisha, India (2012-2014) [166] | ARI | multiple; PCR | 82 | 4.9 | 104 | 3.8 | NA | NA |
| **p619** | East Jakarta, Indonesia (2011-2012) [167] | ARI AND fever | NS and TS; PCR | NA | NA | NA | NA | 306 | 17 |
| **p624** | East Jakarta, Indonesia (2011-2014) [168] | ARI AND fever | NS and TS; PCR | NA | NA | NA | NA | 614 | 18.1 |
| **p630** | multisites, Indonesia (2013-2016)[54] | ARI AND fever | NA; PCR | NA | NA | 284 | 20.4 | NA | NA |
| **p671** | Naples, Italy (2016-2017) [169] | ARI | NPS; PCR | 36 | 13.9 | NA | NA | NA | NA |
| **p679** | multisites, Jordan (2008-2014) [170] | ALRI | NPS and OPS; PCR | NA | NA | 252 | 13 | NA | NA |
| **p693** | Bondo, Kenya (2007-2009)[60] | ALRI | NPS and OPS; PCR | NA | NA | NA | NA | 204 | 59.3 |
| **p704** | Dadaab, Kenya (2010-2012)[61] | ALRI | NPS and OPS; PCR | NA | NA | 180 | 41.1 | NA | NA |
| **p705** | Karemo, Kenya (2009-2012)[62] | ARI | NPS and OPS; PCR | NA | NA | NA | NA | 126 | 10.3 |
| **p738** | Kilifi, Kenya (2007-2010)[124] | ALRI | NPW or NPA; PCR | NA | NA | 149 | 8.1 | NA | NA |
| **p744** | Beirut suburbs and the southern province of Lebanon (2015-2016)[171] | ARI AND fever | NPS; PCR | NA | NA | 68 | 16.2 | NA | NA |
| **p748** | Blantyre, Malawi (2011-2014)[66] | ALRI | NPA; PCR | NA | NA | 82 | 23.2 | NA | NA |
| **p744** | Southern province, Lebanon (2015-2016) [171] | ARI AND fever | Nasopharyngeal swabs; PCR | NA | NA | 68 | 16.2 | NA | NA |
| **p775** | Morocco (2007-2009) [172] | ALRI | NPS and OPS; IFA | NA | NA | 65 | 6.2 | NA | NA |

| Study ID | Location (reference) | Case definition | Specimen and test | ALRI cases for 5-9 y | Proportion for 5-9 y | ALRI cases for 5-14 y | Proportion for 5-14 y | ALRI cases for 5-19 y | Proportion for 5-19 y |
|---|---|---|---|---|---|---|---|---|---|
| **p780** | Maputo, Mozambique (2014-2016) [92] | ARI AND fever | NPS and OPS; PCR | NA | NA | 294 | 4.8 | NA | NA |
| **p781** | 11 countries AFR (2010-2012) [173] | ALRI | NP and OP specimen; PCR | NA | NA | 200 | 10 | NA | NA |
| **p784** | 14 countries AFR and EMR (2006-2010) [174] | ALRI | NA; PCR | 3044 | 11 | 4117 | 11 | NA | NA |
| **p829** | Multi-country (2008-2009) [175] | ARI AND fever | multiple; PCR | NA | NA | 126 | 15.9 | NA | NA |
| **p94** | La Paz, Bolivia (2012-2017)[71] | ARI AND fever | NA; PCR (flu) and IFA (RSV) | NA | NA | NA | NA | 261 | 17.6 |
| **p953** | Lørenskog, Norway (2012-2014) [176] | CXR-pneumonia | NA; NA | NA | NA | NA | NA | 40 | 7.5 |
| **p983** | Oman (2012-2015)[177] | ARI | NA; PCR | NA | NA | 1837 | 29.9 | NA | NA |
| **p99** | Svay Rieng, Cambodia (2015-2016)[75] | ALRI | NPA; PCR | NA | NA | 251 | 23.1 | NA | NA |

**Table S5.6 Details of studies with data on hospital admission rates of respiratory syncytial virus-associated ALRI (per 1,000 children and adolescents per year).** *†‡

| Study ID | Location (reference) | Case definition | Specimen and test | 5-9 y | 5-14 y | 5-19 y |
|---|---|---|---|---|---|---|
| **p1000** | Baguio, Philippines (2010-2011) [7] | ALRI | NPS and OPS; PCR | NA | 0.5 | NA |
| **p1006** | Baguio, Philippines (2012-2014) [8] | ALRI | NPS and OPS; PCR | NA | 0.3 | NA |
| **p108** | Canada (2003-2007; 2010-2014) [98] | ARI | NA; NA | NA | 0 | NA |
| **p1525** | Basel, Switzerland (2001-2002) [100] | ARI | NPS; PCR and rapid antigen test | NA | NA | NA |
| **p1541** | Sa Kaeo and Nakhon Phanom, Thailand (2003-2007) [20] | ARI | NPS; PCR | NA | NA | 0.1 |
| **p1546** | Sa Kaeo and Nakhon Phanom, Thailand (2008-2011) [21] | ARI | NPS; PCR | NA | NA | 0.2 |
| **p1612** | Memphis, Nashville, Salt Lake City, US (2010-2012) [104] | CXR-pneumonia | NPS and OPS and serum; PCR and serology | 0.1 | NA | 0 |
| **p1618** | US (1996-1998) [105] | ALRI | mostly NPS; PCR, culture and EIA (enzyme immunoassay) | NA | NA | 0.1 |
| **p1621** | multisies, US (2001-2012) [178] | ALRI | NA; NA | 0.1 | NA | 0 |
| **p31** | Australia (2006-2015) [179] | ALRI | NA; NA | NA | 0.1 | NA |
| **p328** | China, Hong Kong SAR (2003-2006) [116] | ARI AND fever | NPA; DFA and culture | 0.2 | 0.1 | 0.1 |
| **p454** | Damanhour, Egypt (2009-2012) [44] | ARI AND fever | NPS and OPS; PCR | NA | NA | 0 |
| **p525** | Kiel, Germany (1996-2001) [121] | ALRI | NPA; PCR | NA | 0 | NA |
| **p562** | Santa Rosa and Quetzaltenango, Guatemala (2008-2012) [180] | ARI | NPS and OPS; PCR | NA | NA | 0.1 |
| **p705** | Karemo, Kenya (2009-2012) [62] | ARI | NPS and OPS; PCR | NA | NA | 0.1 |
| **p738** | Kilifi, Kenya (2007-2010) [124] | ALRI | NPW or NPA; PCR | NA | 0 | NA |
| **p748** | Blantyre, Malawi (2011-2014) [66] | ALRI | NPA; PCR | NA | 0 | NA |
| **p94** | La Paz, Bolivia (2012-2017) [71] | ARI AND fever | NA; PCR (flu) and IFA (respiratory syncytial virus) | NA | NA | 0.5 |
| **p957** | Trondheim, Norway (2006-2015) [181] | ALRI | NPA; PCR | NA | 0.1 | NA |

* ARI: any respiratory symptoms (e.g., cough, runny nose, sore throat, shortness of breath, or difficulty in breathing) requiring hospital admission. ARI AND fever: fever or history of fever AND any respiratory symptoms requiring hospital admission. ALRI: any of physician-diagnosed pneumonia (and bronchiolitis), ICD-coded pneumonia (and bronchiolitis), or fever or history of fever AND cough or sour throat AND shortness of breath or difficulty breathing. CXR-pneumonia: chest radiograph confirmed pneumonia. y: years.

† NP: nasopharyngeal. NPS: nasopharyngeal swab. OPS: oropharyngeal swab. PCR: polymerase chain reaction. NS: nasal swab. DFA: direct fluorescent antibody test. EIA: enzyme immunoassay. SPIA: solid-phase immunoassay. TS: throat swab. NPA: nasopharyngeal aspirate. NPW: nasopharyngeal wash. BAL: bronchoalveolar lavage.

‡ When data were reported for other age bands, data were grouped into the closest one of the above age bands. For example, 5-15 years and 5-16 years were re-classified as 5-14 years; 5-17 years and 5-18 years and 6-18 years were re-classified as 5-19 years.



Wang X, *et al. BMJ Global Health* 2021; 6:e006014. doi: 10.1136/bmjgh-2021-006014

**Table S5.7 Details of all studies with data on proportion positives of respiratory syncytial virus in hospitalised ALRI.** *†‡

| Study ID | Location (reference) | Case definition | Specimen and test | ALRI cases for 5-9 y | Proportion for 5-9 y | ALRI cases for 5-14 y | Proportion for 5-14 y | ALRI cases for 5-19 y | Proportion for 5-19 y |
|---|---|---|---|---|---|---|---|---|---|
| **p1000** | Baguio, Philippines (2010-2011) [7] | ALRI | NPS and OPS; PCR | NA | NA | 266 | 6.8 | NA | NA |
| **p1006** | Baguio, Philippines (2012-2014) [8] | ALRI | NPS and OPS; PCR | NA | NA | 268 | 4.5 | NA | NA |
| **p101** | Yaounde, Cameroon (2011-2013) [128] | ARI AND fever | NPS; PCR | NA | NA | 40 | 7.5 | NA | NA |
| **p1018** | Riyadh, Saudi Arabia (2005-2010) [129] | ALRI | NPA; DFA | NA | NA | NA | NA | 50 | 28.0 |
| **p1071** | multisites, South Africa (2009-2012) [83] | ALRI | NPS and TS; PCR | NA | NA | 550 | 6.5 | NA | NA |
| **p1488** | Seongnam, South Korea (2006-2016) [130] | ALRI | Nasal aspirate; PCR | NA | NA | NA | NA | 1723 | 10 |
| **p1489** | Seoul, South Korea (1996-1998) [131] | ALRI | NPA; Culture | NA | NA | 250 | 0 | NA | NA |
| **p1492** | Seoul, South Korea (2006-2007) [132] | ALRI | NP aspiration; PCR | NA | NA | 75 | 1.3 | NA | NA |
| **p1520** | multisies, Spain (2011-2013) [133] | ALRI | NP specimen; PCR | NA | NA | 40 | 10 | NA | NA |
| **p1541** | Sa Kaeo and Nakhon Phanom, Thailand (2003-2007) [20] | ARI | NPS; PCR | NA | NA | NA | NA | 1069 | 5.1 |
| **p1546** | Sa Kaeo and Nakhon Phanom, Thailand (2008-2011) [21] | ARI | NPS; PCR | NA | NA | NA | NA | 1802 | 4.1 |
| **p1554** | Bursa, Turkey (2015-2018) [134] | ARI | NPS or BAL (bronchoalveolar lavage); PCR | NA | NA | NA | NA | 84 | 7.1 |
| **p1557** | Istanbul, Turkey (2010-2011) [135] | ARI | NPA; PCR | 11 | 18.2 | NA | NA | NA | NA |
| **p1612** | Memphis, Nashville, Salt Lake City, US (2010-2012) [104] | CXR-pneumonia | NPS and OPS and serum; PCR and serology | NA | NA | NA | NA | 683 | 7.6 |
| **p1733** | Ho Chi Minh, Vietnam (2004-2008) [137] | ARI | NS, TS, and NPA; PCR | NA | NA | 13 | 7.7 | NA | NA |

* ARI: any respiratory symptoms (e.g., cough, runny nose, sore throat, shortness of breath, or difficulty in breathing) requiring hospital admission. ARI AND fever: fever or history of fever AND any respiratory symptoms requiring hospital admission. ALRI: any of physician-diagnosed pneumonia (and bronchiolitis), ICD-coded pneumonia (and bronchiolitis), or fever or history of fever AND cough or sour throat AND shortness of breath or difficulty breathing. CXR-pneumonia: chest radiograph confirmed pneumonia. y: years.

† NP: nasopharyngeal. NPS: nasopharyngeal swab. OPS: oropharyngeal swab. PCR: polymerase chain reaction. NS: nasal swab. DFA: direct fluorescent antibody test. EIA: enzyme immunoassay. SPIA: solid-phase immunoassay. TS: throat swab. NPA: nasopharyngeal aspirate. NPW: nasopharyngeal wash. BAL: bronchoalveolar lavage.

‡ When data were reported for other age bands, data were grouped into the closest one of the above age bands. For example, 5-11 years were re-classified as 5-9 years; 5-15 years, 5-16 years, 6-12 years, 6-15 years, and 6-16 years were classified as 5-14 years; 5-17 years, 5-18 years, 6-17 years, 7-17 years, and 6-18 years were re-classified as 5-19 years.

| Study ID | Location (reference) | Case definition | Specimen and test | ALRI cases for 5-9 y | Proportion for 5-9 y | ALRI cases for 5-14 y | Proportion for 5-14 y | ALRI cases for 5-19 y | Proportion for 5-19 y |
|---|---|---|---|---|---|---|---|---|---|
| **p1870** | Cairo, Egypt (2010-2014) [139] | ALRI | NPS and OPS; PCR | NA | NA | NA | NA | 191 | 18.8 |
| **P1872** | San Luis Potosı, Mexico (2009-2010) [182] | ALRI | pharyngeal swab/NPS; PCR | NA | NA | NA | NA | 62 | 9.7 |
| **p1873** | Lanzhou, China (2004-2005) [140] | CXR-pneumonia | NPA; DFA | NA | NA | 133 | 12.8 | NA | NA |
| **p1901** | Chengdu, China (2007) [141] | ALRI | NP secretion; DFA | NA | NA | 121 | 2.5 | NA | NA |
| **p1931** | Changsha, China (2015) [142] | ARI | NP secretion; DFA | NA | NA | 285 | 2.1 | NA | NA |
| **P1939** | Jieyang, China (2016-2017) [183] | ALRI | NP secretion; DFA | NA | NA | 67 | 1.5 | | |
| **p216** | Beijing and Shandong, China (2012-2015) [143] | ALRI | NPS, NPA and sputum; PCR | NA | NA | 850 | 4.7 | NA | NA |
| **p221** | Beijing, China (2007-2010) [144] | ALRI | NP secretion; PCR | NA | NA | 358 | 4.5 | NA | NA |
| **p241** | Beijing, China (2011-2012) [145] | ALRI | Tracheal aspirate; PCR | NA | NA | NA | NA | 93 | 47.3 |
| **p245** | Chongqing, China (2006-2008) [184] | ALRI | NPA; PCR | NA | NA | 313 | 0 | NA | NA |
| **p264** | Changsha, China (2007-2008) [146] | ALRI | NPA; PCR | NA | NA | 42 | 11.9 | NA | NA |
| **p311** | Hebei, China (2007-2008) [147] | ALRI | TS; DFA | NA | NA | 361 | 1.1 | NA | NA |
| **p367** | Lanzhou,China (2006-2009) [149] | ALRI | NA; PCR | NA | NA | 67 | 19.4 | NA | NA |
| **p376** | multisites,China (2009-2013) [150] | ALRI | NS or aspirate, sputum, BAL or lung puncture; PCR | NA | NA | 3510 | 4.3 | NA | NA |
| **p386** | Shanghai, China (2003-2006) [151] | ALRI | NPA; DFA | NA | NA | 974 | 3.6 | NA | NA |
| **p387** | Shanghai,China (2013-2015) [152] | ALRI | NP secretion; DFA | NA | NA | NA | NA | 1164 | 1.5 |
| **p388** | Shanghai, China (2016-2017) [153] | ALRI | NPS or sputum specimen; PCR | NA | NA | 106 | 2.8 | NA | NA |
| **p396** | Shantou, Shenzhen and JIeyang, China (2007-2011) [154] | ARI | NPS or sputum specimen; PCR | NA | NA | 112 | 13.4 | NA | NA |
| **p399** | Shantou, Shenzhen and JIeyang, China (2006-2008) [155] | ALRI | PCR; PCR | NA | NA | 30 | 0 | NA | NA |
| **p400** | nine cities, North China (2015) [156] | ALRI | NP secretion; PCR | NA | NA | 331 | 5.1 | NA | NA |
| **p404** | Shenzhen, China (2012-2015) [157] | ARI AND fever | NS; DFA | NA | NA | 1568 | 0.6 | NA | NA |
| **p421** | Tianjin, China (2015-2016) [158] | ARI AND fever | TS; PCR | NA | NA | 62 | 1.6 | NA | NA |
| **p454** | Damanhour, Egypt (2009-2012) [44] | ARI AND fever | NPS and OPS; PCR | NA | NA | NA | NA | 564 | 0.5 |
| **p466** | multisites, Egypt (2007-2014) [90] | ARI AND fever | OPS and NPS; PCR | NA | NA | NA | NA | 1661 | 4.8 |
| **p476** | multisites, Egypt (2012-2015) [162] | ARI AND fever | NPS and OPS; PCR | NA | NA | 980 | 4.2 | NA | NA |
| **p52** | Perth, Australia (2000-2005) [163] | ALRI | NPA; PCR | 103 | 12.6 | NA | NA | NA | NA |
| **p523** | Kiel, Germany (1995-1999) [164] | ALRI | NPA; PCR | NA | NA | 162 | 1.2 | NA | NA |
| **p524** | Germany (1996-1999) [46] | ALRI | NPA; PCR | NA | NA | 262 | 0.8 | NA | NA |

| Study ID | Location (reference) | Case definition | Specimen and test | ALRI cases for 5-9 y | Proportion for 5-9 y | ALRI cases for 5-14 y | Proportion for 5-14 y | ALRI cases for 5-19 y | Proportion for 5-19 y |
|---|---|---|---|---|---|---|---|---|---|
| **p540** | Athens, Greece (pre 2003) [165] | CXR-pneumonia | NPW; PCR | NA | NA | 75 | 2.7 | NA | NA |
| **p562** | Guatemala (2008-2012) [180] | ARI | NPS and OPS; PCR | NA | NA | NA | NA | 403 | 7.7 |
| **p587** | Lucknow, India (2011-2013) [185] | ARI AND fever | NPA; PCR | NA | NA | 59 | 0 | NA | NA |
| **p612** | Odisha, India (2012-2014) [166] | ARI | multiple; PCR | NA | NA | 104 | 0 | NA | NA |
| **p671** | Naples, Italy (2016-2017) [169] | ARI | NPS; PCR | 36 | 2.8 | NA | NA | NA | NA |
| **p705** | Karemo, Kenya (2009-2012) [62] | ARI | NPS and OPS; PCR | NA | NA | NA | NA | 126 | 5.6 |
| **p738** | Kilifi, Kenya (2007-2010) [124] | ALRI | NPW or NPA; PCR | NA | NA | 149 | 5.4 | NA | NA |
| **p742** | Nairobi, Kenya, Slum (2007-2011) [186] | ALRI | NPS and OPS; PCR | NA | NA | NA | NA | 1792 | 6.5 |
| **p829** | Multi-country (2008-2009) [175] | ARI AND fever | multiple; PCR | NA | NA | 125 | 2.4 | NA | NA |
| **p94** | La Paz, Bolivia (2012-2017) [71] | ARI AND fever | NA; PCR (flu) and IFA (respiratory syncytial virus) | NA | NA | NA | NA | 261 | 2.7 |
| **p953** | Lørenskog, Norway (2012-2014) [176] | CXR-pneumonia | NA; NA | NA | NA | NA | NA | 40 | 7.5 |



Wang X, *et al. BMJ Global Health* 2021; 6:e006014. doi: 10.1136/bmjgh-2021-006014

**Table S5.8 Details of all studies with data on proportion positives of human metapneumovirus in hospitalised ALRI.** *†‡

| Study ID | Location (reference) | Case definition | Specimen and test | ALRI cases for 5-9 y | Proportion for 5-9 y | ALRI cases for 5-14 y | Proportion for 5-14 y | ALRI cases for 5-19 y | Proportion for 5-19 y |
|---|---|---|---|---|---|---|---|---|---|
| **p101** | Yaounde, Cameroon (2011-2013) [128] | ARI AND fever | NPS; PCR | NA | NA | 40 | 7.5 | NA | NA |
| **p1071** | multisites, South Africa (2009-2012) [83] | ALRI | NPS and TS; PCR | NA | NA | 550 | 2.4 | NA | NA |
| **p1488** | Seongnam, South Korea (2006-2016) [130] | ALRI | Nasal aspirate; PCR | NA | NA | NA | NA | 1723 | 0.9 |
| **p1492** | Seoul, South Korea (2006-2007) [132] | ALRI | NPA; PCR | NA | NA | 75 | 5.3 | NA | NA |
| **p1520** | multisies, Spain (2011-2013) [133] | ALRI | NP specimen; PCR | NA | NA | 40 | 5 | NA | NA |
| **p1557** | Istanbul, Turkey (2010-2011) [135] | ARI | NPA; PCR | 11 | 0 | NA | NA | NA | NA |
| **p1612** | Memphis, Nashville, Salt Lake City, US (2010-2012) [104] | CXR-pneumonia | NPS and OPS and serum; PCR and serology | NA | NA | NA | NA | 683 | 7.6 |
| **p1733** | Ho Chi Minh, Vietnam (2004-2008) [137] | ARI | NS, TS, and NPA; PCR | NA | NA | 13 | 7.7 | NA | NA |
| **p216** | Beijing and Shandong, China (2012-2015) [143] | ALRI | NPS, NPA and sputum; PCR | NA | NA | 850 | 1.8 | NA | NA |
| **p221** | Beijing, China (2007-2010) [144] | ALRI | NP secretion; PCR | NA | NA | 358 | 0.3 | NA | NA |
| **p241** | Beijing, China (2011-2012) [145] | ALRI | Tracheal aspirate; PCR | NA | NA | NA | NA | 93 | 2.2 |
| **p245** | Chongqing, China (2006-2008) [184] | ALRI | NPA; PCR | NA | NA | 227 | 0.9 | NA | NA |
| **p257** | Guangzhou, China (2013-2016) [187] | ARI | TS; PCR | NA | NA | 734 | 0.7 | NA | NA |
| **p264** | Changsha, China (2007-2008) [146] | ALRI | NPA; PCR | NA | NA | 42 | 11.9 | NA | NA |
| **p367** | Lanzhou,China (2006-2009) [149] | ALRI | NA; PCR | NA | NA | 67 | 6 | NA | NA |
| **p376** | multisites,China (2009-2013) [150] | ALRI | NS, NA, sputum, BAL or lung puncture; PCR | NA | NA | 3510 | 1 | NA | NA |
| **p387** | Shanghai,China (2013-2015) [152] | ALRI | NP secretion; DFA | NA | NA | NA | NA | 1164 | 0.1 |

* ARI: any respiratory symptoms (e.g., cough, runny nose, sore throat, shortness of breath, or difficulty in breathing) requiring hospital admission. ARI AND fever: fever or history of fever AND any respiratory symptoms requiring hospital admission. ALRI: any of physician-diagnosed pneumonia (and bronchiolitis), ICD-coded pneumonia (and bronchiolitis), or fever or history of fever AND cough or sour throat AND shortness of breath or difficulty breathing. CXR-pneumonia: chest radiograph confirmed pneumonia. y: years.

† NP: nasopharyngeal. NPS: nasopharyngeal swab. OPS: oropharyngeal swab. PCR: polymerase chain reaction. NS: nasal swab. DFA: direct fluorescent antibody test. EIA: enzyme immunoassay. SPIA: solid-phase immunoassay. TS: throat swab. NPA: nasopharyngeal aspirate. NPW: nasopharyngeal wash. BAL: bronchoalveolar lavage.

‡ When data were reported for other age bands, data were grouped into the closest one of the above age bands. For example, 5-11 years were re-classified as 5-9 years; 5-15 years, 5-16 years, 6-12 years, 6-15 years, and 6-16 years were classified as 5-14 years; 5-17 years, 5-18 years, 6-17 years, 7-17 years, and 6-18 years were re-classified as 5-19 years.

| Study ID | Location (reference) | Case definition | Specimen and test | ALRI cases for 5-9 y | Proportion for 5-9 y | ALRI cases for 5-14 y | Proportion for 5-14 y | ALRI cases for 5-19 y | Proportion for 5-19 y |
|---|---|---|---|---|---|---|---|---|---|
| **p388** | Shanghai, China (2016-2017) [153] | ALRI | NPS or sputum specimen; PCR | NA | NA | 106 | 0.9 | NA | NA |
| **p396** | Shantou, Shenzhen and JIeyang, China (2007-2011) [154] | ARI | NPS or sputum specimen; PCR | NA | NA | 112 | 0 | NA | NA |
| **p399** | Shantou, Shenzhen and JIeyang, China (2006-2008) [155] | ALRI | PCR; PCR | NA | NA | 30 | 6.7 | NA | NA |
| **p400** | nine cities, North China (2015) [156] | ALRI | NP secretion; PCR | NA | NA | 331 | 0 | NA | NA |
| **p407** | Suzhou, China (2005-2006) [188] | ALRI | Nasal aspirate; PCR | NA | NA | 203 | 3 | NA | NA |
| **p411** | Suzhou, China (2006-2009) [189] | ARI | Nasal aspirate; PCR | 646 | 5.9 | NA | NA | NA | NA |
| **p421** | Tianjin, China (2015-2016) [158] | ARI AND fever | TS; PCR | NA | NA | 62 | 0 | NA | NA |
| **p466** | multisites, Egypt (2007-2014) [90] | ARI AND fever | OPS and NPS; PCR | NA | NA | NA | NA | 361 | 8.3 |
| **p540** | Athens, Greece (pre 2003) [165] | CXR-pneumonia | NPW; PCR | NA | NA | 75 | 1.3 | NA | NA |
| **p587** | Lucknow, India (2011-2013) [185] | ARI AND fever | NPA; PCR | NA | NA | 59 | 5.1 | NA | NA |
| **p612** | Odisha, India (2012-2014) [166] | ARI | multiple; PCR | NA | NA | 104 | 1 | NA | NA |
| **p671** | Naples, Italy (2016-2017) [169] | ARI | NPS; PCR | 36 | 2.8 | NA | NA | NA | NA |
| **p738** | Kilifi, Kenya (2007-2010) [124] | ALRI | NPW or NPA; PCR | NA | NA | 149 | 4 | NA | NA |
| **p829** | Multi-country (2008-2009) [175] | ARI AND fever | multiple; PCR | NA | NA | 125 | 0.8 | NA | NA |
| **p953** | Lørenskog, Norway (2012-2014) [176] | CXR-pneumonia | NA; NA | NA | NA | NA | NA | 40 | 5 |

**Table S5.9 Details of all studies with data on proportion positives of human parainfluenza virus in hospitalised ALRI.** [*†‡]

| Study ID | Location (reference) | Case definition | Specimen and test | ALRI cases for 5-9 y | Proportion for 5-9 y | ALRI cases for 5-14 y | Proportion for 5-14 y | ALRI cases for 5-19 y | Proportion for 5-19 y |
|---|---|---|---|---|---|---|---|---|---|
| **p101** | Yaounde, Cameroon (2011-2013) [128] | ARI AND fever | NPS; PCR | NA | NA | 40 | 5 | NA | NA |
| **p1018** | Riyadh, Saudi Arabia (2005-2010) [129] | ALRI | NPA; DFA | NA | NA | NA | NA | 50 | 0 |
| **p1071** | multisites, South Africa (2009-2012) [83] | ALRI | NPS and TS; PCR | NA | NA | 550 | 3.3 | NA | NA |
| **p1488** | Seongnam, South Korea (2006-2016) [130] | ALRI | Nasal aspirate; PCR | NA | NA | NA | NA | 1723 | 2.4 |
| **p1489** | Seoul, South Korea (1996-1998) [131] | ALRI | NPA; Culture | NA | NA | 250 | 0.4 | NA | NA |
| **p1492** | Seoul, South Korea (2006-2007) [132] | ALRI | NPA; PCR | NA | NA | 75 | 4 | NA | NA |
| **p1520** | multisies, Spain (2011-2013) [133] | ALRI | NP specimen; PCR | NA | NA | 40 | 5 | NA | NA |
| **p1557** | Istanbul, Turkey (2010-2011) [135] | ARI | NPA; PCR | 11 | 9.1 | NA | NA | NA | NA |
| **p1612** | Memphis, Nashville, Salt Lake City, US (2010-2012) [104] | CXR-pneumonia | NPS and OPS and serum; PCR and serology | NA | NA | NA | NA | 683 | 5.1 |
| **p1733** | Ho Chi Minh, Vietnam (2004-2008) [137] | ARI | NS, TS, and NPA; PCR | NA | NA | 13 | 0 | NA | NA |
| **p1870** | Cairo, Egypt (2010-2014) [139] | ALRI | NPS and OPS; PCR | NA | NA | NA | NA | 191 | 3.1 |
| **p1873** | Lanzhou, China (2004-2005) [140] | CXR-pneumonia | NPA; DFA | NA | NA | 133 | 6.8 | NA | NA |
| **p1901** | Chengdu, China (2007) [141] | ALRI | NP secretion; DFA | NA | NA | 121 | 10.7 | NA | NA |
| **p1931** | Changsha, China (2015) [142] | ARI | NP secretion; DFA | NA | NA | 285 | 1.4 | NA | NA |
| **p216** | Beijing and Shandong, China (2012-2015) [143] | ALRI | NPS, NPA and sputum; PCR | NA | NA | 850 | 5.3 | NA | NA |

[*] ARI: any respiratory symptoms (e.g., cough, runny nose, sore throat, shortness of breath, or difficulty in breathing) requiring hospital admission. ARI AND fever: fever or history of fever AND any respiratory symptoms requiring hospital admission. ALRI: any of physician-diagnosed pneumonia (and bronchiolitis), ICD-coded pneumonia (and bronchiolitis), or fever or history of fever AND cough or sour throat AND shortness of breath or difficulty breathing. CXR-pneumonia: chest radiograph confirmed pneumonia. y: years.

[†] NP: nasopharyngeal. NPS: nasopharyngeal swab. OPS: oropharyngeal swab. PCR: polymerase chain reaction. NS: nasal swab. DFA: direct fluorescent antibody test. EIA: enzyme immunoassay. SPIA: solid-phase immunoassay. TS: throat swab. NPA: nasopharyngeal aspirate. NPW: nasopharyngeal wash. BAL: bronchoalveolar lavage.

[‡] When data were reported for other age bands, data were grouped into the closest one of the above age bands. For example, 5-11 years were re-classified as 5-9 years; 5-15 years, 5-16 years, 6-12 years, 6-15 years, and 6-16 years were classified as 5-14 years; 5-17 years, 5-18 years, 6-17 years, 7-17 years, and 6-18 years were re-classified as 5-19 years.



Wang X, *et al. BMJ Global Health* 2021; 6:e006014. doi: 10.1136/bmjgh-2021-006014

| Study ID | Location (reference) | Case definition | Specimen and test | ALRI cases for 5-9 y | Proportion for 5-9 y | ALRI cases for 5-14 y | Proportion for 5-14 y | ALRI cases for 5-19 y | Proportion for 5-19 y |
|---|---|---|---|---|---|---|---|---|---|
| **p221** | Beijing, China (2007-2010) [144] | ALRI | NP secretion; PCR | NA | NA | 358 | 3.9 | NA | NA |
| **P231** | Beijing, China (2014-2016)[190] | ARI AND fever | NPS, TS, or sputum; PCR | NA | NA | 157 | 5.1 | NA | NA |
| **p241** | Beijing, China (2011-2012) [145] | ALRI | Tracheal aspirate; PCR | NA | NA | NA | NA | 93 | 14 |
| **p264** | Changsha, China (2007-2008) [146] | ALRI | NPA; PCR | NA | NA | 42 | 4.8 | NA | NA |
| **p311** | Hebei, China (2007-2008) [147] | ALRI | TS; DFA | NA | NA | 361 | 1.7 | NA | NA |
| **p367** | Lanzhou,China (2006-2009) [149] | ALRI | NA; PCR | NA | NA | 67 | 6 | NA | NA |
| **p376** | multisites,China (2009-2013) [150] | ALRI | NS, NA, sputum, BAL or lung puncture; PCR | NA | NA | 3510 | 4.4 | NA | NA |
| **p386** | Shanghai, China (2003-2006) [151] | ALRI | NPA; DFA | NA | NA | 974 | 1.7 | NA | NA |
| **p387** | Shanghai,China (2013-2015) [152] | ALRI | NP secretion; DFA | NA | NA | NA | NA | 1164 | 1.1 |
| **p396** | Shantou, Shenzhen and JIeyang, China (2007-2011) [154] | ARI | NPS or sputum specimen; PCR | NA | NA | 112 | 8 | NA | NA |
| **p399** | Shantou, Shenzhen and JIeyang, China (2006-2008) [155] | ALRI | PCR; PCR | NA | NA | 30 | 0 | NA | NA |
| **p400** | nine cities, North China (2015) [156] | ALRI | NP secretion; PCR | NA | NA | 331 | 4.8 | NA | NA |
| **p404** | Shenzhen, China (2012-2015) [157] | ARI AND fever | NS; DFA | NA | NA | 1568 | 0.3 | NA | NA |
| **p421** | Tianjin, China (2015-2016) [158] | ARI AND fever | TS; PCR | NA | NA | 62 | 1.6 | NA | NA |
| **p466** | multisites, Egypt (2007-2014) [90] | ARI AND fever | OPS and NPS; PCR | NA | NA | NA | NA | 326 | 4 |
| **p52** | Perth, Australia (2000-2005) [163] | ALRI | NPA; PCR | 97 | 2.1 | NA | NA | NA | NA |
| **p523** | Kiel, Germany (1995-1999) [164] | ALRI | NPA; PCR | NA | NA | 36 | 2.8 | NA | NA |
| **p540** | Athens, Greece (pre 2003) [165] | CXR-pneumonia | NPW; PCR | NA | NA | 75 | 8 | NA | NA |
| **p612** | Odisha, India (2012-2014) [166] | ARI | multiple; PCR | NA | NA | 104 | 0 | NA | NA |
| **p671** | Naples, Italy (2016-2017) [169] | ARI | NPS; PCR | 36 | 5.6 | NA | NA | NA | NA |
| **p738** | Kilifi, Kenya (2007-2010) [124] | ALRI | NPW or NPA; PCR | NA | NA | 149 | 5.4 | NA | NA |
| **p829** | Multi-country (2008-2009) [175] | ARI AND fever | multiple; PCR | NA | NA | 125 | 2.4 | NA | NA |
| **p953** | Lørenskog, Norway (2012-2014) [176] | CXR-pneumonia | NA; NA | NA | NA | NA | NA | 40 | 2.5 |

**Table S5.10 Details of studies with data on hospital admission rates of human metapneumovirus-associated ALRI and human parainfluenza-virus-associated ALRI (per 1,000 children and adolescents per year) *†‡**

| Virus | Study ID | Location (reference) | Case definition | Specimen and test | 5-9 y | 5-14 y | 5-19 y |
|---|---|---|---|---|---|---|---|
| **Human metapneumovirus** | p1487 | Soweto, South Africa (2009-2013) [191] | ALRI | NA; PCR | NA | NA | 0 |
| | p1612 | Memphis, Nashville, Salt Lake City, US (2010-2012) [104] | CXR-pneumonia | NPS and OPS and serum; PCR and serology | 0.1 | NA | 0 |
| | p1631 | Salt Lake City Utah, US (2007-2013) [192] | ALRI | NA; DFA and culture | NA | NA | 0.1 |
| | p561 | Santa Rosa and Quetzaltenango, Guatemala (2007-2012) [193] | ARI | OPS and NPS; PCR | NA | NA | 0 |
| | p738 | Kilifi, Kenya (2007-2010) [124] | ALRI | NPW or NPA; PCR | NA | 0 | NA |
| | p957 | Trondheim, Norway (2006-2015) [181] | ALRI | NPA; PCR | NA | 0.1 | NA |
| **Human parainfluenza virus** | p1537 | Sa Kaeo and Nakhon Phanom, Thailand (2003-2007) [194] | ARI | NPS; PCR and serology | 0.2 | NA | 0.1 |
| | p1612 | Memphis, Nashville, Salt Lake City, US (2010-2012) [104] | CXR-pneumonia | NPS and OPS and serum; PCR and serology | 0.1 | NA | 0 |
| | p1618 | US (1996-1998) [105] | ALRI | mostly NPS; PCR, culture and EIA (enzyme immunoassay) | NA | NA | 0.2 |
| | p296 | China, Hong Kong SAR (1998-2012) [191] | ARI | NPA; Immunofluorescence | 0.4 | 0.2 | NA |
| | p328 | China, Hong Kong SAR (2003-2006) [116] | ARI AND fever | NPA; DFA and culture | 0.6 | 0.3 | 0.2 |
| | p738 | Kilifi, Kenya (2007-2010) [124] | ALRI | NPW or NPA; PCR | NA | 0 | NA |

* ARI: any respiratory symptoms (e.g., cough, runny nose, sore throat, shortness of breath, or difficulty in breathing) requiring hospital admission. ARI AND fever: fever or history of fever AND any respiratory symptoms requiring hospital admission. ALRI: any of physician-diagnosed pneumonia (and bronchiolitis), ICD-coded pneumonia (and bronchiolitis), or fever or history of fever AND cough or sour throat AND shortness of breath or difficulty breathing. CXR-pneumonia: chest radiograph confirmed pneumonia. y: years.

† NP: nasopharyngeal. NPS: nasopharyngeal swab. OPS: oropharyngeal swab. PCR: polymerase chain reaction. NS: nasal swab. DFA: direct fluorescent antibody test. EIA: enzyme immunoassay. SPIA: solid-phase immunoassay. TS: throat swab. NPA: nasopharyngeal aspirate. NPW: nasopharyngeal wash. BAL: bronchoalveolar lavage.

‡ When data were reported for other age bands, data were grouped into the closest one of the above age bands. For example, 5-17 years and 5-18 years were re-classified as 5-19 years.

**Table S5.12 Details of all studies with data on in-hospital case-fatality ratios (hCFRs) of influenza-associated ALRI. *†‡**

| Study ID | Location (reference) | Case definition | Cases for 5-9 y | hCFR for 5-9 y (%) | Cases for 5-14 y | hCFR for 5-14 y (%) | Cases for 5-19 y | hCFR for 5-19 y (%) |
|---|---|---|---|---|---|---|---|---|
| **p1070** | Singapore (2013-2014) [97] | ARI AND fever | 270 | 0.7 | NA | NA | 418 | 0.7 |
| **p146** | multisites, Canada (2004-2005) [195] | ARI | NA | NA | 80 | 1.2 | NA | NA |
| **p1512** | Catalonia, Spain (2010-2015) [196] | ALRI | NA | NA | 46 | 2.2 | NA | NA |
| **p1518** | Spain (2009-2015) [197] | ICD-coded lab-confirmed influenza | NA | NA | 1525 | 1 | NA | NA |
| **p1519** | Spain (2010-2016) [99] | ALRI | NA | NA | 644 | 3.1 | NA | NA |
| **p1565** | multisites, Uganda (2010-2015) [86] | ARI | NA | NA | 20 | 5 | NA | NA |
| **p1637** | Salt Lake County, Utah, US (2001-2004) [107] | ARI | NA | NA | NA | NA | 34 | 2.9 |
| **p212** | multisites, Canada (2003-2004) [198] | ARI | NA | NA | NA | NA | 82 | 1.2 |
| **P23** | Australia (2014)[199] | ARI | 0 | 0 | 1 | 2.0 | NA | NA |
| **P244** | China (2010-2011)[200] | ARI AND fever | NA | NA | 109 | 2.8 | NA | NA |
| **p296** | China, Hong Kong SAR (1998-2012) [191] | ARI | 2384 | 0 | 3075 | 0.1 | NA | NA |
| **p466** | multisites, Egypt (2007-2014) [90] | ARI AND fever | 132 | 0.8 | 195 | 1 | 264 | 2.3 |
| **p491** | Turku, Finland (1988-2004) [119] | ARI | NA | NA | 105 | 1 | NA | NA |
| **p520** | Germany (2004-2010) [120] | ICD-coded lab-confirmed influenza | NA | NA | 8189 | 0.5 | NA | NA |
| **p58** | Sydney and Perth, Australia (2011-2013) [201] | ARI | 185 | 1.1 | 264 | 0.8 | NA | NA |
| **p611** | Madhya Pradesh, India (2009-2015) [202] | ARI AND fever | NA | NA | 116 | 1.7 | NA | NA |
| **p624** | East Jakarta, Indonesia (2011-2014) [168] | ARI AND fever | NA | NA | NA | NA | 111 | 0 |
| **p655** | Ireland (2010-2011) [123] | ARI AND fever | 53 | 3.8 | 91 | 3.3 | NA | NA |
| **p679** | multisites, Jordan (2008-2014) [170] | ALRI | NA | NA | 31 | 3.2 | NA | NA |
| **p749** | Mexico (2013-2014) [203] | ALRI | NA | NA | 127 | 7.1 | NA | NA |
| **p758** | Mexico city, Mexico (2011-2012) [204] | ALRI | NA | NA | 95 | 3.2 | NA | NA |
| **p794** | eight African countries (2009-2012) [93] | ARI AND fever | NA | NA | NA | NA | 320 | 0 |
| **p804** | Nine eastern Europe countries (2009-2012) [205] | ALRI | NA | NA | 207 | 1.9 | NA | NA |

* ARI: any respiratory symptoms (e.g., cough, runny nose, sore throat, shortness of breath, or difficulty in breathing) requiring hospital admission. ARI AND fever: fever or history of fever AND any respiratory symptoms requiring hospital admission. ALRI: any of physician-diagnosed pneumonia (and bronchiolitis), ICD-coded pneumonia (and bronchiolitis), or fever or history of fever AND cough or sour throat AND shortness of breath or difficulty breathing. CXR-pneumonia: chest radiograph confirmed pneumonia. y: years.

† NP: nasopharyngeal. NPS: nasopharyngeal swab. OPS: oropharyngeal swab. PCR: polymerase chain reaction. NS: nasal swab. DFA: direct fluorescent antibody test. EIA: enzyme immunoassay. SPIA: solid-phase immunoassay. TS: throat swab. NPA: nasopharyngeal aspirate. NPW: nasopharyngeal wash. BAL: bronchoalveolar lavage.

‡ When data were reported for other age bands, data were grouped into the closest one of the above age bands. For example, 5-11 years were re-classified as 5-9 years; 5-15 years and 6-14 years were classified as 5-14 years; 5-17 years, and 5-18 years were re-classified as 5-19 years.

| Study ID | Location (reference) | Case definition | Cases for 5-9 y | hCFR for 5-9 y (%) | Cases for 5-14 y | hCFR for 5-14 y (%) | Cases for 5-19 y | hCFR for 5-19 y (%) |
|---|---|---|---|---|---|---|---|---|
| **p818** | Four countries (Russia, Turkey, China, Spain) (2013-2014) [206] | ARI AND one systemic symptom | NA | NA | NA | NA | 102 | 0 |
| **p963** | Sohar, Oman (2010-2013) [125] | ARI | NA | NA | 53 | 1.9 | NA | NA |
| **p983** | Oman (2012-2015) [74] | ARI | NA | NA | 519 | 1.3 | NA | NA |

**Table S5.13 Estimates of in-hospital case-fatality ratios (hCFRs) of influenza-associated ALRI and in-hospital mortality in children and adolescents aged 5-14 years, by World Bank income region.**

| | World Bank income region | No. of studies | hCFR (%)[§] | In-hospital mortality for 5-14 years[**] |
|---|---|---|---|---|
| **Using hCFR data for 5-14 years or 5-19 years[††]** | Low & lower middle | 5 | 0.8 (0.2-3.3) | 3700 (500-27100) |
| | Upper middle | 6 | 2.6 (1.3-5.2) | 8200 (2300-27600) |
| | High | 15 | 0.9 (0.5-1.6) | 200 (100-500) |
| | **Global** | | | 12100 (2900-55200) |
| **Using hCFR data for 5-14 years[‡‡]** | Low & lower middle | 3 | 1.5 (0.6-3.6) | 7100 (1600-29600) |
| | Upper middle | 5 | 3.4 (2-5.8) | 10700 (3500-31600) |
| | High | 12 | 0.9 (0.5-1.7) | 200 (100-500) |
| | **Global** | | | 18000 (5200-61700) |

Given the paucity of influenza-specific hCFR data, we did a primary analysis for influenza-associated ALRI in-hospital deaths for 5-14 years. Table S6.11 shows the preliminary estimates. An estimated 10,500 (UR 2,200-49,800) influenza-associated ALRI in-hospital deaths occurred among children and adolescents aged 5-14 years globally when including all hCFR data (for 5-14 years or 5-19 years). When restricted the analysis to including only hCFR data for 5-14 years, we estimated 18,900 (UR 4,700-75,600) influenza-associated ALRI in-hospital deaths globally for 5-14 years. We were unable to estimate influenza-associated ALRI in-hospital deaths for 5-19 years due to the lack of data. We were unable to estimate in-hospital deaths for the other three viruses given the paucity of data on hCFRs of respiratory syncytial virus (four studies), human metapneumovirus (one study), and human parainfluenza virus (one study).

[§] Estimates from meta-analyses.
[**] Estimated by combining hCFRs and hospital admissions of IFV-associated ALRI by regions.
[††] hCFR estimates were based on all data for 5-14 years or 5-19 years.
[‡‡] hCFR estimates were based on the data for 5-14 years.

**Table S5.14 Details of all studies with data on in-hospital case-fatality ratios (hCFRs) of ALRI associated with respiratory syncytial virus, human metapneumovirus, and human parainfluenza virus.** [*†]

| | Study ID | Location (reference) | Case definition | Age | Virus-confirmed cases | hCFR (%) |
|---|---|---|---|---|---|---|
| **Respiratory syncytial virus** | p296 | China, Hong Kong SAR (1998-2012) [191] | ARI | 5-14 y | 318 | 2.2 |
| | p562 | Santa Rosa and Quetzaltenango, Guatemala (2008-2012)[180] | ARI | 5-17 y | 30 | 6.67 |
| | p752 | Mexico city, Mexico (2004-2008)[207] | ARI | 5-18 y | 20 | 10 |
| | p1511 | Seremban, Malaysia (2008-2013)[208] | ALRI | 5-16 y | 12 | 0 |
| **Human metapneumovirus** | p1611 | Columbus, Ohio, US (2007-2010)[209] | ARI | 5-15 y | 23 | 0 |
| **Human parainfluenza virus** | p296 | China, Hong Kong SAR (1998-2012) [191] | ARI | 5-14 y | 3075 | 0 |
| | p1567 | England and Wales, UK (1989-1997)[210] | ALRI | 5-14 y | 241 | 2.9 |

[*] ARI: any respiratory symptoms (e.g., cough, runny nose, sore throat, shortness of breath, or difficulty in breathing) requiring hospital admission. ARI AND fever: fever or history of fever AND any respiratory symptoms requiring hospital admission. ALRI: any of physician-diagnosed pneumonia (and bronchiolitis), ICD-coded pneumonia (and bronchiolitis), or fever or history of fever AND cough or sour throat AND shortness of breath or difficulty breathing. CXR-pneumonia: chest radiograph confirmed pneumonia. y: years.

[†] NP: nasopharyngeal. NPS: nasopharyngeal swab. OPS: oropharyngeal swab. PCR: polymerase chain reaction. NS: nasal swab. DFA: direct fluorescent antibody test. EIA: enzyme immunoassay. SPIA: solid-phase immunoassay. TS: throat swab. NPA: nasopharyngeal aspirate. NPW: nasopharyngeal wash. BAL: bronchoalveolar lavage.

# Appendix 6 Assessment of risk of bias

## Table S6.1 Assessment criteria

| Category | Description | Risk of bias |
|---|---|---|
| Study design | Studies where the cases were prospectively enrolled | Low |
| | Other study designs | High |
| Adjustment for healthcare utilization (only for hospital admission rate studies) | Meeting either of the following:<br>1. Including all or main hospitals in the catchment area;<br>2. Not including main hospitals, but adjusting for the proportion of patients admitted in the study hospitals | Low |
| | Not including main hospitals OR no related description; AND no adjustment for the proportion of patients admitted in the study hospitals | High |
| Patient groups excluded | No exclusions that may affect estimates | Low |
| | Exclusions that may affect estimates, such as:<br>1. Excluding children and adolescents with high-risk conditions.<br>2. Other exclusions that may affect estimates | High |
| Case definition | Any of the following:<br>1. physician-diagnosed ALRI or pneumonia<br>2. ALRI diagnosed based on clinical symptoms or signs, e.g., fever AND cough or sore throat AND shortness of breath or difficulty breathing<br>3. chest radiograph confirmed pneumonia<br>4. ARI requiring hospital admission; ARI AND fever requiring hospital admission. | Low |
| | Other definitions that are more specific or less specific, e.g., ARI or fever requiring hospital admission; hospitalised with infection infections (ICD-code) | High |
| Sampling strategy (only for studies with data on viruses) | The proportion of cases tested is available AND either of the following:<br>1. >=90% of eligible patients are tested.<br>2. Testing a systematic/random sample of patients. | Low |
| | <90% of eligible cases are tested; OR<br>The proportion of cases that are tested is unavailable. | High |
| Test (only for studies with data on viruses) | PCR;<br>Or using other diagnostic tests, but confirming negative samples with PCR | Low |
| | 1. Other diagnostic tests, e.g., culture, IFA, DFA.<br>2. No mention of test methods | High |

**Table S6.2 Risk of bias for studies reporting data on ALRI hospital admission rates.**

| Study ID | Location; period | Study design | Adjustment for healthcare utilisation | Patient groups excluded | Case definition |
|---|---|---|---|---|---|
| **p45** | Mornington, Australia; 2007-2011[43] | High | High | Low | Low |
| **p55** | Western Australia; 1996-2012[48] | High | Low | Low | Low |
| **p62** | Brazil; 2003-2004[53] | High | Low | Low | Low |
| **p73** | Brazil; 2005-2015 [64] | High | Low | Low | Low |
| **p94** | La Paz, Bolivia; 2012-2017[71] | Low | Low | Low | Low |
| **p99** | Svay Rieng, Cambodia; 2015-2016 [75] | Low | Low | Low | Low |
| **p99** | Siem Reap, Cambodia; 2015-2016 [75] | Low | Low | Low | Low |
| **p99** | Kampong Cham, Cambodia; 2015-2016 [75] | Low | Low | Low | Low |
| **p113** | Canada; 2004-2010[10] | High | Low | Low | Low |
| **p117** | Edmonton, Canada; 2000-2002[11] | Low | High | High | Low |
| **p125** | multisites, Canada; 1995-2001[12] | High | Low | Low | Low |
| **p165** | Ontario, Canada; 1992-2014[31] | High | Low | Low | Low |
| **p226** | Beijing, China; 2017-2018[34] | Low | Low | Low | Low |
| **p286** | China, Hong Kong SAR; 2005[35] | High | Low | Low | Low |
| **p321** | China, Hong Kong SAR; 2011-2015[36] | High | Low | Low | Low |
| **p355** | Jingzhou, China; 2010-2012[37] | Low | Low | Low | Low |
| **p416** | Suzhou, China; 2010-2014[38] | High | Low | Low | Low |
| **p420** | Taiwan, China; 1997-2004[39] | High | Low | Low | Low |
| **p433** | Denmark; 1995-1999[40] | High | Low | Low | Low |
| **p440** | Denmark; 1997-2011[41] | High | Low | Low | Low |
| **p447** | Ecuador; 2011-2015 [42] | High | Low | Low | Low |
| **p454** | Damanhour, Egypt; 2009-2012[44] | Low | Low | Low | Low |
| **p509** | Rhône-Alpes, France; 2005-2010[45] | High | Low | Low | Low |
| **p524** | Kiel, Germany; 1996-1999[46] | Low | Low | Low | Low |
| **p532** | Shai-Osudoku and Ningo-Prampram, Ghana; 2013-2015[47] | Low | Low | Low | Low |
| **p557** | Santa Rosa and Quetzaltenango, Guatemala; 2007-2011[49] | Low | Low | Low | Low |
| **p566** | Hungary; 2006-2011[50] | High | Low | Low | Low |
| **p567** | Ballabgarh, India; 2010-2012[51] | Low | Low | Low | Low |
| **p567** | Vadu, India; 2010-2012[51] | Low | Low | Low | Low |
| **p572** | Ballabgarh, India; 2012-2014[52] | Low | Low | Low | Low |
| **p630** | Wonosari, Indonesia; 2013-2016[54] | Low | Low | Low | Low |
| **p630** | Kanudjoso Djati, Indonesia; 2013-2016[54] | Low | Low | Low | Low |
| **p630** | Deli Serdang, Indonesia; 2013-2016[54] | Low | Low | Low | Low |
| **p633** | Iran; 2015 [55] | High | High | Low | Low |
| **p646** | multisites, Iran; 2012-2013 [56] | Low | Low | Low | Low |
| **p669** | Negev, Israel; 1989-1991[57] | High | Low | Low | Low |
| **p672** | Veneto, Italy; 2004-2012[58] | High | Low | Low | Low |
| **p689** | Bondo, Kenya; 2001-2003[59] | High | Low | Low | Low |

| Study ID | Location; period | Study design | Adjustment for healthcare utilisation | Patient groups excluded | Case definition |
|---|---|---|---|---|---|
| **p693** | Bondo, Kenya; 2007-2009[60] | Low | Low | Low | Low |
| **p704** | Dadaab, Kenya; 2010-2012[61] | Low | Low | Low | Low |
| **p705** | Karemo, Kenya; 2009-2012[62] | Low | Low | Low | Low |
| **p706** | Kenya; 2012-2014[63] | low | Low | Low | Low |
| **p731** | Kilifi, Kenya; 2002-2015[65] | Low | Low | Low | Low |
| **p748** | Blantyre, Malawi; 2011-2014[66] | Low | Low | Low | Low |
| **p774** | multisites, Mongolia; 2007-2012[67] | Low | Low | Low | Low |
| **p877** | Netherlands; 2001-2007[68] | High | Low | Low | Low |
| **p882** | Netherlands; 2008-2011[69] | High | Low | Low | Low |
| **p939** | Auckland, New Zealand; 1993-1996[70] | Low | Low | Low | Low |
| **p945** | Leo´n, Nicaragua; 2008-2015[72] | Low | Low | Low | Low |
| **p954** | Norway; 2008-2009[73] | High | Low | Low | Low |
| **p983** | Oman; 2012-2015[74] | High | Low | Low | Low |
| **p1000** | Baguio, Philippines; 2010-2011[7] | Low | Low | Low | Low |
| **p1006** | Baguio, Philippines; 2012-2014[8] | Low | Low | Low | Low |
| **p1015** | Chrzanów County, Poland; 2006-2008[9] | High | Low | Low | Low |
| **p1015** | Inowroclaw County, Poland; 2006-2008[9] | High | Low | Low | Low |
| **p1483** | South Africa; 2007-2012[13] | High | Low | Low | Low |
| **p1503** | South Korea; 2002-2005[14] | Low | Low | Low | Low |
| **p1505** | South Korea; 2007-2014[15] | High | Low | Low | Low |
| **p1516** | Spain; 1995-1998[16] | High | Low | Low | Low |
| **p1517** | Spain; 2001-2014[17] | High | Low | Low | Low |
| **p1522** | Sweden; 2005[18] | High | Low | Low | Low |
| **p1532** | Sa Kaeo, Thailand; 2002-2003[19] | Low | Low | Low | Low |
| **p1541** | Sa Kaeo and Nakhon Phanom, Thailand; 2003-2007[20] | Low | Low | Low | Low |
| **p1546** | Sa Kaeo and Nakhon Phanom, Thailand; 2008-2011[21] | Low | Low | Low | Low |
| **p1548** | Sfax, Tunisia; 2003-2015[22] | High | High | Low | Low |
| **p1566** | Wakiso, Uganda; 2013-2016[23] | Low | Low | Low | Low |
| **p1566** | Mbarara, Uganda; 2013-2016[23] | Low | Low | Low | Low |
| **p1566** | Tororo, Uganda; 2013-2016[23] | Low | Low | Low | Low |
| **p1571** | England, UK; 2001-2013[24] | High | Low | Low | Low |
| **p1577** | North East England, UK; 2001-2002[25] | Low | Low | Low | Low |
| **p1578** | North East England, UK; 2008-2009[26] | Low | Low | Low | Low |
| **p1583** | Scotland, UK; 1981-2005[27] | High | Low | Low | Low |
| **p1587** | Scotland, UK; 2000-2012[28] | High | Low | Low | Low |
| **p1604** | two municipalities, Uruguay; 2009-2012[29] | Low | High | Low | Low |
| **p1640** | US; 1996-2011[30] | High | Low | Low | Low |
| **p1654** | US; 2012-2014[32] | High | Low | Low | Low |
| **p1871** | Beijing, China; 2014-2016[33] | Low | Low | Low | Low |

**Table S6.3 Risk of bias for studies with data on influenza-associated ALRI hospital admission rates**

| Study ID | Location; period | Study design | Adjustment for healthcare utilisation | Patient groups excluded | Case definition | Sampling strategy | Test |
|---|---|---|---|---|---|---|---|
| **p39** | Australia; 2006-2015[118] | High | Low | Low | High | High | High |
| **p94** | La Paz, Bolivia; 2012-2017[71] | Low | Low | Low | Low | Low | Low |
| **p99** | Svay Rieng, Cambodia; 2015-2016 [75] | Low | Low | Low | Low | High | Low |
| **p99** | Siem Reap, Cambodia; 2015-2016 [75] | Low | Low | Low | Low | High | Low |
| **p99** | Kampong Cham, Cambodia; 2015-2016 [75] | Low | Low | Low | Low | High | Low |
| **p105** | Canada; 2003-2008[96] | High | Low | Low | High | High | High |
| **p108** | Canada; 2003-2007; 2010-2014[98] | High | Low | Low | Low | High | High |
| **p226** | Beijing, China; 2017-2018[34] | Low | Low | Low | Low | High | Low |
| **p328** | China, Hong Kong SAR; 2003-2006[116] | Low | Low | High | Low | Low | High |
| **p330** | China, Hong Kong SAR; 2005-2011[117] | High | Low | Low | High | High | High |
| **p355** | Jingzhou, China; 2010-2012[37] | Low | Low | Low | Low | Low | High |
| **p491** | Turku, Finland; 1988-2004[119] | High | Low | Low | Low | High | High |
| **p520** | Germany; 2004-2010[120] | High | Low | Low | High | High | High |
| **p525** | Kiel, Germany; 1996-2001[121] | Low | Low | Low | Low | High | Low |
| **p532** | Shai-Osudoku and Ningo-Prampram, Ghana; 2013-2015[47] | Low | Low | Low | Low | High | Low |
| **p536** | Athens, Greece; 2002-2005[122] | Low | Low | Low | High | High | Low |
| **p567** | Ballabgarh, India; 2010-2012[51] | Low | Low | Low | Low | Low | Low |
| **p567** | Vadu, India; 2010-2012[51] | Low | Low | Low | Low | Low | Low |
| **p630** | Wonosari, Indonesia; 2013-2016[54] | Low | Low | Low | Low | Low | Low |
| **p630** | Kanudjoso Djati, Indonesia; 2013-2016[54] | Low | Low | Low | Low | Low | Low |
| **p630** | Deli Serdang, Indonesia; 2013-2016[54] | Low | Low | Low | Low | Low | Low |
| **p655** | Ireland; 2010-2011[123] | High | Low | Low | Low | High | Low |
| **p693** | Bondo, Kenya; 2007-2009[60] | Low | Low | Low | Low | High | Low |
| **p704** | Dadaab, Kenya; 2010-2012[61] | Low | Low | Low | Low | Low | Low |
| **p705** | Karemo, Kenya; 2009-2012[62] | Low | Low | Low | Low | High | Low |
| **p738** | Kilifi, Kenya; 2007-2010[124] | Low | Low | Low | Low | High | Low |
| **p748** | Blantyre, Malawi; 2011-2014[66] | Low | Low | Low | Low | Low | Low |
| **p963** | Sohar, Oman; 2010-2013[125] | Low | Low | Low | Low | Low | Low |

| Study ID | Location; period | Study design | Adjustment for healthcare utilisation | Patient groups excluded | Case definition | Sampling strategy | Test |
|---|---|---|---|---|---|---|---|
| **p963** | Ibra, Oman; 2010-2013[125] | Low | Low | Low | Low | Low | Low |
| **p963** | SQH, Oman; 2010-2013[125] | Low | Low | Low | Low | Low | Low |
| **p983** | Oman; 2012-2015[74] | High | Low | Low | Low | High | Low |
| **p995** | multisites, Peru; 2009-2015[126] | Low | Low | Low | Low | Low | Low |
| **p1000** | Baguio, Philippines; 2010-2011[7] | Low | Low | Low | Low | Low | Low |
| **p1006** | Baguio, Philippines; 2012-2014[8] | Low | Low | Low | Low | Low | Low |
| **p1070** | Singapore; 2013-2014[97] | High | Low | Low | Low | High | High |
| **p1519** | Spain; 2010-2016[99] | High | Low | Low | Low | High | High |
| **p1525** | Basel, Switzerland; 2001-2002[100] | High | High | High | Low | High | High |
| **p1545** | Sa Kaeo and Nakhon Phanom, Thailand; 2005-2008 Incidence, Seasonality and Mortality Associated with Influenza Pneumonia in Thailand: 2005-2008. | Low | Low | Low | Low | High | Low |
| **p1566** | Wakiso, Uganda; 2013-2016[23] | Low | Low | Low | Low | High | Low |
| **p1566** | Mbarara, Uganda; 2013-2016[23] | Low | Low | Low | Low | High | Low |
| **p1566** | Tororo, Uganda; 2013-2016[23] | Low | Low | Low | Low | High | Low |
| **p1574** | England, UK; 2010-2015 [102] | High | Low | Low | Low | High | High |
| **p1610** | Colorado, US; 2004-2008[103] | High | Low | Low | Low | High | High |
| **p1612** | Memphis, Nashville, Salt Lake City, US; 2010-2012[104] | Low | Low | High | Low | Low | High |
| **p1618** | US; 1996-1998[105] | Low | Low | Low | Low | High | High |
| **p1625** | Philadelphia, US; 2000-2004[106] | High | Low | Low | Low | High | High |
| **p1637** | Salt Lake County, Utah, US; 2001-2004[107] | High | Low | Low | Low | High | High |
| **p1656** | US; 2003-2008[108] | Low | Low | Low | Low | High | Low |
| **p1668** | US; 2008-2011[109] | Low | Low | Low | Low | High | Low |
| **p1671** | US; 2012-2013[110] | Low | Low | Low | Low | High | Low |
| **p1672** | US; 2013-2014[111] | Low | Low | Low | Low | High | Low |
| **p1686** | US; 2017-2018[112] | Low | Low | Low | Low | High | Low |
| **p1690** | US; 2018-2019[113] | Low | Low | Low | Low | High | Low |
| **p1700** | Utah, US; 2016-2017[114] | Low | Low | Low | Low | High | Low |
| **p1735** | Nha Trang, Viet Nam; 2007-2012[115] | Low | Low | Low | Low | High | Low |

| Study ID | Location; period | Study design | Adjustment for healthcare utilisation | Patient groups excluded | Case definition | Sampling strategy | Test |
|---|---|---|---|---|---|---|---|
| **p1871** | Beijing, China; 2014-2016[33] | Low | Low | Low | Low | Low | Low |
| **p774** | multisites, Mongolia; 2007-2012[67] | Low | Low | Low | Low | Low | High |

**Table S6.4 Risk of bias for studies with data on in-hospital case-fatality ratios (hCFRs) of ALRI**

| Study ID | Location; period | Study design | Patient groups excluded | Case definition |
|---|---|---|---|---|
| **p1015** | Chrzanów County, Poland; 2006-2008[9] | High | Low | Low |
| **p1071** | multisites, South Africa; 2009-2012[83] | Low | Low | Low |
| **p113** | Canada; 2004-2010[10] | High | Low | Low |
| **p1505** | South Korea; 2007-2014[15] | High | Low | Low |
| **p1513** | Spain; 1995-1996[84] | High | Low | Low |
| **p1527** | Sa Kaeo; 2002-2003[85] | Low | Low | Low |
| **p1541** | Sa Kaeo and Nakhon Phanom, Thailand; 2003-2007[20] | Low | Low | Low |
| **p1548** | Sfax, Tunisia; 2003-2015[22] | High | Low | Low |
| **p1565** | multisites, Uganda; 2010-2015[86] | Low | Low | Low |
| **p1568** | England, UK; 2000-2007[87] | High | Low | Low |
| **p1602** | Paysandú and Salto, Uruguay; 2001-2004[88] | Low | Low | Low |
| **p1654** | US; 2012-2014[32] | High | Low | Low |
| **p246** | Guangzhou, China; 2009-2012[89] | High | Low | Low |
| **p321** | China, Hong Kong SAR; 2011-2015[36] | High | Low | Low |
| **p420** | Taiwan, China; 1997-2004[39] | High | Low | Low |
| **p440** | Denmark; 1997-2011[41] | High | Low | Low |
| **p466** | multisites, Egypt; 2007-2014[90] | Low | Low | Low |
| **p566** | Hungary; 2006-2011[50] | High | Low | Low |
| **p62** | Brazil; 2003-2007[53] | High | Low | Low |
| **p633** | Iran; 2015[55] | High | Low | Low |
| **p689** | Bondo, Kenya; 2001-2003[59] | High | Low | Low |
| **p739** | multisites, Kenya; 2014-2018[91] | High | Low | Low |
| **p780** | Maputo, Mozambique; 2014-2016[92] | Low | Low | Low |
| **p794** | eight African countries; 2009-2012[93] | Low | Low | Low |
| **p90** | Rio de Janeiro, Brazil; 1996-2011[94] | Low | Low | Low |
| **p983** | Oman; 2012-2015[74] | High | Low | Low |
| **p992** | Asuncion, Paraguay; 2004-2013[95] | High | Low | Low |

**Table S6.5 Risk of bias for studies with data on proportion positives of influenza in hospitalised ALRI**

| Study ID | Location; period | Study design | Patient groups excluded | Case definition | Sampling strategy | Test |
|---|---|---|---|---|---|---|
| **p52** | Perth, Australia; 2000-2005[163] | High | Low | Low | High | Low |
| **p101** | Yaounde, Cameroon; 2011-2013[128] | Low | Low | Low | Low | Low |
| **p216** | Beijing and Shandong, China; 2012-2015[143] | Low | Low | Low | High | Low |
| **p221** | Beijing, China; 2007-2010[144] | Low | Low | Low | High | Low |
| **p241** | Beijing, China; 2011-2012[145] | High | Low | Low | High | Low |
| **p264** | Changsha, China; 2007-2008[146] | Low | Low | Low | High | Low |
| **p311** | Hebei, China; 2007-2008[147] | Low | Low | Low | Low | High |
| **p331** | China, Hong Kong SAR; 2009-2013[148] | Low | Low | Low | Low | High |
| **p367** | Lanzhou,China; 2006-2009[149] | High | Low | Low | Low | Low |
| **p376** | multisites,China; 2009-2013[150] | Low | Low | Low | Low | Low |
| **p386** | Shanghai, China; 2003-2006[151] | High | Low | Low | High | High |
| **p387** | Shanghai,China; 2013-2015[152] | Low | Low | Low | Low | High |
| **p388** | Shanghai, China; 2016-2017[153] | Low | Low | Low | Low | Low |
| **p399** | Shantou, Shenzhen and Jieyang, China; 2006-2008[155] | Low | Low | Low | High | Low |
| **p400** | nine cities, North China; 2015[156] | Low | Low | Low | High | Low |
| **p404** | Shenzhen, China; 2012-2015[157] | Low | Low | Low | High | High |
| **p421** | Tianjin, China; 2015-2016[158] | Low | Low | Low | High | Low |
| **p427** | Zhejiang, China; 2011-2015[159] | Low | Low | Low | High | Low |
| **p428** | multisites, DR Congo; 2015[160] | Low | Low | Low | Low | Low |
| **p452** | Damanhour, Egypt; 2013[161] | Low | Low | Low | Low | Low |
| **p466** | multisites, Egypt; 2007-2014[90] | Low | Low | Low | Low | Low |
| **p476** | multisites, Egypt; 2012-2015[162] | Low | Low | Low | High | Low |
| **p523** | Kiel, Germany; 1995-1999[164] | Low | Low | Low | High | Low |
| **p540** | Athens, Greece; (pre 2003) [165] | Low | High | Low | Low | Low |
| **p612** | Odisha, India; 2012-2014[166] | Low | Low | Low | High | Low |
| **p619** | East Jakarta, Indonesia; 2011-2012[167] | Low | Low | Low | Low | Low |
| **p624** | East Jakarta, Indonesia; 2011-2014[168] | Low | Low | Low | Low | Low |
| **p671** | Naples, Italy; 2016-2017[169] | High | Low | Low | High | Low |
| **p679** | multisites, Jordan; 2008-2014[170] | Low | Low | Low | Low | Low |
| **p744** | Southern province, Lebanon; 2015-2016[171] | Low | Low | Low | Low | Low |
| **p744** | Beirut, Lebanon; 2015-2016 [171] | Low | Low | Low | Low | Low |
| **p775** | Morocco; 2007-2009[172] | Low | Low | Low | High | High |
| **p780** | Maputo, Mozambique; 2014-2016[92] | Low | Low | Low | Low | Low |
| **p781** | 11 countries AFR; 2010-2012[173] | Low | Low | Low | High | Low |
| **p784** | 14 countries AFR and EMR; 2006-2010 [174] | Low | Low | Low | High | Low |
| **p829** | Multi-country; 2008-2009[175] | High | Low | Low | High | Low |
| **p953** | Lørenskog, Norway; 2012-2014[176] | Low | High | Low | Low | Low |
| **p1018** | Riyadh, Saudi Arabia; 2005-2010[129] | High | Low | Low | High | High |
| **p1071** | multisites, South Africa; 2009-2012[83] | Low | Low | Low | Low | Low |
| **p1488** | Seongnam, South Korea; 2006-2016[130] | High | Low | Low | High | Low |

| Study ID | Location; period | Study design | Patient groups excluded | Case definition | Sampling strategy | Test |
|---|---|---|---|---|---|---|
| **p1489** | Seoul, South Korea; 1996-1998[131] | Low | Low | Low | High | High |
| **p1492** | Seoul, South Korea; 2006-2007[132] | Low | Low | Low | High | Low |
| **p1520** | multisies, Spain; 2011-2013[133] | Low | High | Low | Low | Low |
| **p1520** | London, UK; 2011-2013 [133] | Low | High | Low | Low | Low |
| **p1554** | Bursa, Turkey; 2015-2018[134] | High | Low | Low | High | Low |
| **p1557** | Istanbul, Turkey; 2010-2011[135] | High | Low | Low | High | Low |
| **p1565** | multisites, Uganda; 2010-2015[86] | Low | Low | Low | High | Low |
| **p1733** | Ho Chi Minh, Vietnam; 2004-2008[137] | Low | Low | Low | High | Low |
| **p1739** | Vietnam; 2011-2014[138] | Low | Low | Low | High | Low |
| **p1870** | Cairo, Egypt; 2010-2014[139] | Low | Low | Low | High | Low |
| **p1873** | Lanzhou, China; 2004-2005[140] | Low | Low | Low | Low | High |
| **p1901** | Chengdu, China; 2007[141] | Low | Low | Low | Low | High |
| **p1931** | Changsha, China; 2015[142] | Low | Low | Low | Low | High |
| **p396** | Shantou, Shenzhen and JIeyang, China; 2007-2011[154] | Low | Low | Low | High | Low |



Wang X, *et al. BMJ Global Health* 2021; 6:e006014. doi: 10.1136/bmjgh-2021-006014

**Table S6.5 Risk of bias for studies with data on in-hospital case-fatality ratios (hCFRs) of influenza-associated ALRI**

| Study ID | Location; period | Study design | Patient groups excluded | Case definition | Sampling strategy | Test |
|---|---|---|---|---|---|---|
| **p1070** | Singapore; 2013-2014[97] | High | Low | Low | High | High |
| **p146** | multisites, Canada; 2004-2005[195] | High | Low | Low | High | High |
| **p1512** | Catalonia, Spain; 2010-2015[196] | High | Low | Low | High | High |
| **p1518** | Spain; 2009-2015[197] | High | Low | High | High | High |
| **p1519** | Spain; 2010-2016[99] | High | Low | Low | High | High |
| **p1565** | multisites, Uganda; 2010-2015[86] | Low | Low | Low | High | Low |
| **p1637** | Salt Lake County, Utah, US; 2001-2004[107] | High | Low | Low | High | High |
| **p212** | multisites, Canada; 2003-2004[198] | High | Low | Low | High | High |
| **p296** | China, Hong Kong SAR; 1998-2012[191] | High | Low | Low | High | High |
| **p466** | multisites, Egypt; 2007-2014[90] | Low | Low | Low | Low | Low |
| **p491** | Turku, Finland; 1988-2004[119] | High | Low | Low | High | High |
| **p520** | Germany; 2004-2010[120] | High | Low | High | High | High |
| **p58** | Sydney and Perth, Australia; 2011-2013[201] | High | Low | Low | High | Low |
| **p611** | Madhya Pradesh, India; 2009-2015[202] | High | Low | Low | High | Low |
| **p624** | East Jakarta, Indonesia; 2011-2014[168] | Low | Low | Low | Low | Low |
| **p655** | Ireland; 2010-2011[123] | High | Low | Low | High | Low |
| **p679** | multisites, Jordan; 2008-2014[170] | Low | Low | Low | Low | Low |
| **p749** | Mexico; 2013-2014[203] | High | Low | Low | High | Low |
| **p758** | Mexico city, Mexico; 2011-2012[204] | High | Low | Low | High | Low |
| **p794** | eight African countries; 2009-2012[93] | Low | Low | Low | High | Low |
| **p804** | Nine eastern Europe countries; 2009-2012[205] | Low | Low | Low | Low | High |
| **p818** | Four countries (Russia, Turkey, China, Spain); 2013-2014[206] | Low | Low | Low | Low | Low |
| **p963** | Sohar, Oman; 2010-2013[125] | Low | Low | Low | Low | Low |
| **p983** | Oman; 2012-2015[74] | High | Low | Low | High | Low |

**Table S6.5 Risk of bias for studies with data on proportion positives of respiratory syncytial virus in hospitalised ALRI**

| Study ID | Location; period | Study design | Patient groups excluded | Case definition | Sampling strategy | Test |
|---|---|---|---|---|---|---|
| **p52** | Perth, Australia; 2000-2005[163] | High | Low | Low | High | Low |
| **p94** | La Paz, Bolivia; 2012-2017[71] | Low | Low | Low | Low | Low |
| **p101** | Yaounde, Cameroon; 2011-2013[128] | Low | Low | Low | Low | Low |
| **p216** | Beijing and Shandong, China; 2012-2015[143] | Low | Low | Low | High | Low |
| **p221** | Beijing, China; 2007-2010[144] | Low | Low | Low | High | Low |
| **p241** | Beijing, China; 2011-2012[145] | High | Low | Low | High | Low |
| **p245** | Chongqing, China; 2006-2008[184] | Low | Low | Low | Low | Low |
| **p264** | Changsha, China; 2007-2008[146] | Low | Low | Low | High | Low |
| **p311** | Hebei, China; 2007-2008[147] | Low | Low | Low | Low | High |
| **p367** | Lanzhou,China; 2006-2009[149] | High | Low | Low | Low | Low |
| **p376** | multisites,China; 2009-2013[150] | Low | Low | Low | Low | Low |
| **p386** | Shanghai, China; 2003-2006[151] | High | Low | Low | High | High |
| **p387** | Shanghai,China; 2013-2015[152] | Low | Low | Low | Low | High |
| **p388** | Shanghai, China; 2016-2017[153] | Low | Low | Low | Low | Low |
| **p396** | Shantou, Shenzhen and Jieyang, China; 2007-2011[154] | Low | Low | Low | High | Low |
| **p399** | Shantou, Shenzhen and Jieyang, China; 2006-2008[155] | Low | Low | Low | High | Low |
| **p400** | nine cities, North China; 2015[156] | Low | Low | Low | High | Low |
| **p404** | Shenzhen, China; 2012-2015[157] | Low | Low | Low | High | High |
| **p421** | Tianjin, China; 2015-2016[158] | Low | Low | Low | High | Low |
| **p454** | Damanhour, Egypt; 2009-2012[44] | Low | Low | Low | Low | Low |
| **p466** | multisites, Egypt; 2007-2014[90] | Low | Low | Low | Low | Low |
| **p476** | multisites, Egypt; 2012-2015[162] | Low | Low | Low | High | Low |
| **p523** | Kiel, Germany; 1995-1999[164] | Low | Low | Low | High | Low |
| **p524** | Germany; 1996-1999[46] | Low | Low | Low | High | Low |
| **p540** | Athens, Greece; (pre 2003) [165] | Low | High | Low | Low | Low |
| **p562** | Guatemala; 2008-2012[180] | Low | Low | Low | High | Low |
| **p587** | Lucknow, India; 2011-2013[185] | Low | Low | Low | High | Low |
| **p612** | Odisha, India; 2012-2014[166] | Low | Low | Low | High | Low |
| **p671** | Naples, Italy; 2016-2017[169] | High | Low | Low | High | Low |
| **p705** | Karemo, Kenya; 2009-2012[62] | Low | Low | Low | High | Low |
| **p738** | Kilifi, Kenya; 2007-2010[124] | Low | Low | Low | High | Low |
| **p742** | Nairobi, Kenya, Slum; 2007-2011[186] | Low | Low | Low | High | Low |
| **p829** | Multi-country; 2008-2009[175] | High | Low | Low | High | Low |
| **p953** | Lørenskog, Norway; 2012-2014[176] | Low | High | Low | Low | Low |
| **p1000** | Baguio, Philippines; 2010-2011[7] | Low | Low | Low | Low | Low |
| **p1006** | Baguio, Philippines; 2012-2014[8] | Low | Low | Low | Low | Low |
| **p1018** | Riyadh, Saudi Arabia; 2005-2010[129] | High | Low | Low | High | High |
| **p1071** | multisites, South Africa; 2009-2012[83] | Low | Low | Low | Low | Low |
| **p1488** | Seongnam, South Korea; 2006-2016[130] | High | Low | Low | High | Low |

| Study ID | Location; period | Study design | Patient groups excluded | Case definition | Sampling strategy | Test |
|---|---|---|---|---|---|---|
| **p1489** | Seoul, South Korea; 1996-1998[131] | Low | Low | Low | High | High |
| **p1492** | Seoul, South Korea; 2006-2007[132] | Low | Low | Low | High | Low |
| **p1520** | multisies, Spain; 2011-2013[133] | Low | High | Low | Low | Low |
| **p1520** | London, UK; 2011-2013[133] | Low | High | Low | Low | Low |
| **p1541** | Sa Kaeo and Nakhon Phanom, Thailand; 2003-2007[20] | Low | Low | Low | High | Low |
| **p1546** | Sa Kaeo and Nakhon Phanom, Thailand; 2008-2011[21] | Low | Low | Low | High | Low |
| **p1554** | Bursa, Turkey; 2015-2018[134] | High | Low | Low | High | Low |
| **p1557** | Istanbul, Turkey; 2010-2011[135] | High | Low | Low | High | Low |
| **p1612** | Memphis, Nashville, Salt Lake City, US; 2010-2012[104] | Low | High | Low | Low | High |
| **p1733** | Ho Chi Minh, Vietnam; 2004-2008[137] | Low | Low | Low | High | Low |
| **p1870** | Cairo, Egypt; 2010-2014[139] | Low | Low | Low | High | Low |
| **P1872** | San Luis Potosı, Mexico; 2009-2010[182] | Low | Low | Low | Low | Low |
| **p1873** | Lanzhou, China; 2004-2005[140] | Low | Low | Low | Low | High |
| **p1901** | Chengdu, China; 2007[141] | Low | Low | Low | Low | High |
| **p1931** | Changsha, China; 2015[142] | Low | Low | Low | Low | High |
| **P1939** | Jieyang, China; 2016-2017[183] | Low | Low | Low | High | High |

**Table S6.6 Risk of bias for studies with data on proportion positives of human metapneumovirus in hospitalised ALRI**

| Study ID | Location; period | Study design | Patient groups excluded | Case definition | Sampling strategy | Test |
|---|---|---|---|---|---|---|
| **p101** | Yaounde, Cameroon; 2011-2013[128] | Low | Low | Low | Low | Low |
| **p216** | Beijing and Shandong, China; 2012-2015[143] | Low | Low | Low | High | Low |
| **p221** | Beijing, China; 2007-2010[144] | Low | Low | Low | High | Low |
| **p241** | Beijing, China; 2011-2012[145] | High | Low | Low | High | Low |
| **p245** | Chongqing, China; 2006-2008[184] | Low | Low | Low | Low | Low |
| **p257** | Guangzhou, China; 2013-2016[187] | Low | Low | Low | High | Low |
| **p264** | Changsha, China; 2007-2008[146] | Low | Low | Low | High | Low |
| **p367** | Lanzhou,China; 2006-2009[149] | High | Low | Low | Low | Low |
| **p376** | multisites,China; 2009-2013[150] | Low | Low | Low | Low | Low |
| **p387** | Shanghai,China; 2013-2015[152] | Low | Low | Low | Low | High |
| **p388** | Shanghai, China; 2016-2017[153] | Low | Low | Low | Low | Low |
| **p396** | Shantou, Shenzhen and JIeyang, China; 2007-2011[154] | Low | Low | Low | High | Low |
| **p399** | Shantou, Shenzhen and JIeyang, China; 2006-2008[155] | Low | Low | Low | High | Low |
| **p400** | nine cities, North China; 2015[156] | Low | Low | Low | High | Low |
| **p407** | Suzhou, China; 2005-2006[188] | High | Low | Low | High | Low |
| **p411** | Suzhou, China; 2006-2009[189] | Low | Low | Low | Low | Low |
| **p421** | Tianjin, China; 2015-2016[158] | Low | Low | Low | High | Low |
| **p466** | multisites, Egypt; 2007-2014[90] | Low | Low | Low | Low | Low |
| **p540** | Athens, Greece; (pre 2003) [165] | Low | High | Low | Low | Low |
| **p587** | Lucknow, India; 2011-2013[185] | Low | Low | Low | High | Low |
| **p612** | Odisha, India; 2012-2014[166] | Low | Low | Low | High | Low |
| **p671** | Naples, Italy; 2016-2017[169] | High | Low | Low | High | Low |
| **p738** | Kilifi, Kenya; 2007-2010[124] | Low | Low | Low | High | Low |
| **p829** | Multi-country; 2008-2009[175] | High | Low | Low | High | Low |
| **p953** | Lørenskog, Norway; 2012-2014[176] | Low | High | Low | Low | Low |
| **p1071** | multisites, South Africa; 2009-2012[83] | Low | Low | Low | Low | Low |
| **p1488** | Seongnam, South Korea; 2006-2016[130] | High | Low | Low | High | Low |
| **p1492** | Seoul, South Korea; 2006-2007[132] | Low | Low | Low | High | Low |
| **p1520** | multisies, Spain; 2011-2013[133] | Low | High | Low | Low | Low |
| **p1520** | London, UK; 2011-2013[133] | Low | High | Low | Low | Low |
| **p1557** | Istanbul, Turkey; 2010-2011[135] | High | Low | Low | High | Low |
| **p1612** | Memphis, Nashville, Salt Lake City, US; 2010-2012[104] | Low | High | Low | Low | High |
| **p1733** | Ho Chi Minh, Vietnam; 2004-2008[137] | Low | Low | Low | High | Low |

**Table S6.7 Risk of bias for studies with data on proportion positives of human parainfluenza virus in hospitalised ALRI**

| Study ID | Location; period | Study design | Patient groups excluded | Case definition | Sampling strategy | Test |
|---|---|---|---|---|---|---|
| **p52** | Perth, Australia; 2000-2005[163] | High | Low | Low | High | Low |
| **p101** | Yaounde, Cameroon; 2011-2013[128] | Low | Low | Low | Low | Low |
| **p216** | Beijing and Shandong, China; 2012-2015[143] | Low | Low | Low | High | Low |
| **p221** | Beijing, China; 2007-2010[144] | Low | Low | Low | High | Low |
| **p241** | Beijing, China; 2011-2012[145] | High | Low | Low | High | Low |
| **p264** | Changsha, China; 2007-2008[146] | Low | Low | Low | High | Low |
| **p311** | Hebei, China; 2007-2008[147] | Low | Low | Low | Low | High |
| **p367** | Lanzhou,China; 2006-2009[149] | High | Low | Low | Low | Low |
| **p376** | multisites,China; 2009-2013[150] | Low | Low | Low | Low | Low |
| **p386** | Shanghai, China; 2003-2006[151] | High | Low | Low | High | High |
| **p387** | Shanghai,China; 2013-2015[152] | Low | Low | Low | Low | High |
| **p396** | Shantou, Shenzhen and Jieyang, China; 2007-2011[154] | Low | Low | Low | High | Low |
| **p399** | Shantou, Shenzhen and Jieyang, China; 2006-2008[155] | Low | Low | Low | High | Low |
| **p400** | nine cities, North China; 2015[156] | Low | Low | Low | High | Low |
| **p404** | Shenzhen, China; 2012-2015[157] | Low | Low | Low | High | High |
| **p421** | Tianjin, China; 2015-2016[158] | Low | Low | Low | High | Low |
| **p466** | multisites, Egypt; 2007-2014[90] | Low | Low | Low | Low | Low |
| **p523** | Kiel, Germany; 1995-1999[164] | Low | Low | Low | High | Low |
| **p540** | Athens, Greece; (pre 2003) [165] | Low | High | Low | Low | Low |
| **p612** | Odisha, India; 2012-2014[166] | Low | Low | Low | High | Low |
| **p671** | Naples, Italy; 2016-2017[169] | High | Low | Low | High | Low |
| **p738** | Kilifi, Kenya; 2007-2010[124] | Low | Low | Low | High | Low |
| **p829** | Multi-country; 2008-2009[175] | High | Low | Low | High | Low |
| **p953** | Lørenskog, Norway; 2012-2014[176] | Low | High | Low | Low | Low |
| **p1018** | Riyadh, Saudi Arabia; 2005-2010[129] | High | Low | Low | High | High |
| **p1071** | multisites, South Africa; 2009-2012[83] | Low | Low | Low | Low | Low |
| **p1488** | Seongnam, South Korea; 2006-2016[130] | High | Low | Low | High | Low |
| **p1489** | Seoul, South Korea; 1996-1998[131] | Low | Low | Low | High | High |
| **p1492** | Seoul, South Korea; 2006-2007[132] | Low | Low | Low | High | Low |
| **p1520** | multisies, Spain; 2011-2013[133] | Low | High | Low | Low | Low |
| **p1520** | London, UK; 2011-2013[133] | Low | High | Low | Low | Low |
| **p1557** | Istanbul, Turkey; 2010-2011[135] | High | Low | Low | High | Low |
| **p1612** | Memphis, Nashville, Salt Lake City, US; 2010-2012[104] | Low | High | Low | Low | High |
| **p1733** | Ho Chi Minh, Vietnam; 2004-2008[137] | Low | Low | Low | High | Low |
| **p1870** | Cairo, Egypt; 2010-2014[139] | Low | Low | Low | High | Low |
| **p1873** | Lanzhou, China; 2004-2005[140] | Low | Low | Low | Low | High |
| **p1901** | Chengdu, China; 2007[141] | Low | Low | Low | Low | High |
| **p1931** | Changsha, China; 2015[142] | Low | Low | Low | Low | High |

GATHER Guidelines for Accurate and Transparent Health Estimates Reporting

## Checklist of information that should be included in new reports of global health estimates

| Item # | Checklist item | Reported on page # |
|---|---|---|
| **Objectives and funding** | | |
| 1 | Define the indicator(s), populations (including age, sex, and geographic entities), and time period(s) for which estimates were made. | Summary |
| 2 | List the funding sources for the work. | Summary |
| **Data Inputs** | | |
| *For all data inputs from multiple sources that are synthesized as part of the study:* | | |
| 3 | Describe how the data were identified and how the data were accessed. | Page4 |
| 4 | Specify the inclusion and exclusion criteria. Identify all ad-hoc exclusions. | Page5 |
| 5 | Provide information on all included data sources and their main characteristics. For each data source used, report reference information or contact name/institution, population represented, data collection method, year(s) of data collection, sex and age range, diagnostic criteria or measurement method, and sample size, as relevant. | Appendix pp18-41 |
| 6 | Identify and describe any categories of input data that have potentially important biases (e.g., based on characteristics listed in item 5). | Appendix pp42-55 |
| *For data inputs that contribute to the analysis but were not synthesized as part of the study:* | | |
| 7 | Describe and give sources for any other data inputs. | Appendix pp18-41 |
| *For all data inputs:* | | |
| 8 | Provide all data inputs in a file format from which data can be efficiently extracted (e.g., a spreadsheet rather than a PDF), including all relevant meta-data listed in item 5. For any data inputs that cannot be shared because of ethical or legal reasons, such as third-party ownership, provide a contact name or the name of the institution that retains the right to the data. | Data have been presented in the supplementary material. Data will be made available on Edinburgh Datashare (https://datashare.is.ed.ac.uk/) later. |
| **Data analysis** | | |
| 9 | Provide a conceptual overview of the data analysis method. A diagram may be helpful. | Figure 1 |
| 10 | Provide a detailed description of all steps of the analysis, including mathematical formulae. This description should cover, as relevant, data cleaning, data pre-processing, data adjustments and weighting of data sources, and mathematical or statistical model(s). | Page 6-7; Appendix pp5-7 |
| 11 | Describe how candidate models were evaluated and how the final model(s) were selected. | Page 7 |
| 12 | Provide the results of an evaluation of model performance, if done, as well as the results of any relevant sensitivity analysis. | Appendix pp8-14 |
| 13 | Describe methods for calculating uncertainty of the estimates. State which sources of uncertainty were, and were not, accounted for in the uncertainty analysis. | Page 6. |
| 14 | State how analytic or statistical source code used to generate estimates can be accessed. | Major codes used in this study will be made available upon request. |
| **Results and Discussion** | | |
| 15 | Provide published estimates in a file format from which data can be efficiently extracted. | Estimates can be easily extracted in main table and supplementary table. Main tables will also be provided on Edinburgh Datashare (https://datashare.is.ed.ac.uk/) later. |
| 16 | Report a quantitative measure of the uncertainty of the estimates (e.g. uncertainty intervals). | Uncertainty is reported for burden estimates in main tables, supplementary tables and Results. |
| 17 | Interpret results in light of existing evidence. If updating a previous set of estimates, describe the reasons for changes in estimates. | Page 13. |
| 18 | Discuss limitations of the estimates. Include a discussion of any modelling assumptions or data limitations that affect interpretation of the estimates. | Page 11-13 |

References


1. Wang X, Li Y, O'Brien KL, et al. Global burden of respiratory infections associated with seasonal influenza in children under 5 years in 2018: a systematic review and modelling study. *The Lancet Global Health* 2020; **8**(4): e497-e510.
2. Sterne JAC, White IR, Carlin JB, et al. Multiple imputation for missing data in epidemiological and clinical research: potential and pitfalls. *BMJ* 2009; **338**: b2393.
3. United Nations Department of Economic and Social Affairs Population Division. World Population Prospects 2019. Online Edition. Rev. 1.; 2019.
4. Rücker G, Krahn U, König J, Efthimiou O, G. S. Network Meta-Analysis using Frequentist Methods. R package version 1.2-1, 2020.
5. Rubin DB. Multiple Imputation for Nonresponse in Surveys. New York: Wiley; 1987.
6. Honaker J, King G, Blackwell M. Amelia II: A Program for Missing Data. *2011* 2011; **45**(7): 47.
7. Tallo VL, Kamigaki T, Tan AG, et al. Estimating influenza outpatients' and inpatients' incidences from 2009 to 2011 in a tropical urban setting in the Philippines. *Influenza and other Respiratory Viruses* 2014; **8**(2): 159-68.
8. Kamigaki T, Aldey PP, Mercado ES, et al. Estimates of influenza and respiratory syncytial virus incidences with fraction modeling approach in Baguio City, the Philippines, 2012-2014. *Influenza and other Respiratory Viruses* 2017; **11**(4): 311-8.
9. Harat R, Gorny G, Jorgensen L, et al. A retrospective study of hospitalized pneumonia in two Polish counties (2006-2008). *Pneumonologia i alergologia polska* 2013; **81**(5): 429-38.
10. McNeil SA, Qizilbash N, Ye J, et al. A Retrospective Study of the Clinical Burden of Hospitalized All-Cause and Pneumococcal Pneumonia in Canada. *Canadian Respiratory Journal* 2016; **2016**: 3605834.
11. Marrie TJ, Huang JQ. Epidemiology of community-acquired pneumonia in Edmonton, Alberta: an emergency department-based study. *Canadian Respiratory Journal* 2005; **12**(3): 139-42.
12. Alaghehbandan R, Gates KD, MacDonald D. Hospitalization due to pneumonia among Innu, Inuit and non-Aboriginal communities, Newfoundland and Labrador, Canada. *International Journal of Infectious Diseases* 2007; **11**(1): 23-8.
13. Kyeyagalire R, Tempia S, Cohen AL, et al. Hospitalizations associated with influenza and respiratory syncytial virus among patients attending a network of private hospitals in South Africa, 2007-2012. *BMC Infectious Diseases* 2014; **14**: 694.
14. Kim SA, Kilgore PE, Lee SY, Nyambat B, Ki M. Trends in pneumonia and influenza-associated hospitalizations in South Korea, 2002-2005. *Journal of Health, Population & Nutrition* 2011; **29**(6): 574-82.
15. Shin EJ, Kim Y, Jeong JY, Jung YM, Lee MH, Chung EH. The changes of prevalence and etiology of pediatric pneumonia from national emergency department information system in Korea, between 2007 and 2014. *Korean Journal of Pediatrics* 2018; **61**(9): 291-300.
16. Gil A, San-Martin M, Carrasco P, Gonzalez A. Epidemiology of pneumonia hospitalizations in Spain, 1995-1998. *Journal of Infection* 2002; **44**(2): 84-7.
17. Trujillo IJ, de Andres AL, Hernandez-Barrera V, Martinez-Huedo MA, de Miguel-Diez J, Jimenez-Garcia R. Decrease in the incidence and in hospital mortality of community-acquired pneumonia among children in Spain (2001-2014). *Vaccine* 2017; **35**(30): 3733-40.

18. Silfverdal SA, Berg S, Hemlin C, Jokinen I. The cost-burden of paediatric pneumococcal disease in Sweden and the potential cost-effectiveness of prevention using 7-valent pneumococcal vaccine. *Vaccine* 2009; **27**(10): 1601-8.
19. Jordan HT, Prapasiri P, Areerat P, et al. A comparison of population-based pneumonia surveillance and health-seeking behavior in two provinces in rural Thailand. *International Journal of Infectious Diseases* 2009; **13**(3): 355-61.
20. Fry AM, Chittaganpitch M, Baggett HC, et al. The burden of hospitalized lower respiratory tract infection due to respiratory syncytial virus in rural Thailand. *PLoS ONE [Electronic Resource]* 2010; **5**(11): e15098.
21. Naorat S, Chittaganpitch M, Thamthitiwat S, et al. Hospitalizations for acute lower respiratory tract infection due to respiratory syncytial virus in Thailand, 2008-2011. *Journal of Infectious Diseases* 2013; **208**: S238-45.
22. Ben Ayed H, Yaich S, Ben Jmaa M, et al. Pediatric respiratory tract diseases: Chronological trends and perspectives. *Pediatrics International* 2018; **60**(1): 76-82.
23. Emukule GO, Namagambo B, Owor N, et al. Influenza-associated pneumonia hospitalizations in Uganda, 2013-2016. *PLoS ONE* 2019; **14**(7).
24. Been JV, Millett C, Lee T, Schayck CPv, Sheikh A. Smoke-free legislation and childhood hospitalisations for respiratory tract infections. *European Respiratory Journal* 2015; **46**(3): 697-706.
25. Clark JE, Hammal D, Hampton F, Spencer D, Parker L. Epidemiology of community-acquired pneumonia in children seen in hospital. *Epidemiology & Infection* 2007; **135**(2): 262-9.
26. Elemraid MA, Rushton SP, Shirley MDF, et al. Impact of the 7-valent pneumococcal conjugate vaccine on the incidence of childhood pneumonia. *Epidemiology and Infection* 2013; **141**(8): 1697-704.
27. Roxburgh CS, Youngson GG, Townend JA, Turner SW. Trends in pneumonia and empyema in Scottish children in the past 25 years. *Archives of Disease in Childhood* 2008; **93**(4): 316-8.
28. Nair H, Watts AT, Williams LJ, et al. Pneumonia hospitalisations in Scotland following the introduction of pneumococcal conjugate vaccination in young children. *BMC Infectious Diseases* 2016; **16**: 390.
29. Hortal M, Estevan M, Meny M, Iraola I, Laurani H. Impact of pneumococcal conjugate vaccines on the incidence of pneumonia in hospitalized children after five years of its introduction in Uruguay. *PLoS ONE [Electronic Resource]* 2014; **9**(6): e98567.
30. Chang DH, Bednarczyk RA, Becker ER, et al. Trends in U.S. hospitalizations and inpatient deaths from pneumonia and influenza, 1996-2011. *Vaccine* 2016; **34**(4): 486-94.
31. Luca DL, Kwong JC, Chu A, et al. Impact of Pneumococcal Vaccination on Pneumonia Hospitalizations and Related Costs in Ontario: A Population-Based Ecological Study. *Clinical Infectious Diseases* 2018; **66**(4): 541-7.
32. Hayes BH, Haberling DL, Kennedy JL, Varma JK, Fry AM, Vora NM. Burden of Pneumonia-Associated Hospitalizations: United States, 2001-2014. *Chest* 2018; **153**(2): 427-37.
33. 赵小娟. 2014-2016 年北京市怀柔区严重急性呼吸道感染病例流感病毒感染情况及其住院率分析. *实用预防医学* 2019; **9**(26).

34. Chen J, Wang L, Sun L. Epidemiological characteristics of infectious diseases in children in child care settings in Huairou district of Beijing, 2008-2017. [Chinese]. *Disease Surveillance* 2019; **34**(8): 746-9.
35. Chan-Yeung M, Lai CKW, Chan KS, et al. The burden of lung disease in Hong Kong: A report from the Hong Kong Thoracic Society. *Respirology* 2008; **13**(SUPPL. 4): S133-S65.
36. Li X, Blais JE, Wong ICK, et al. Population-based estimates of the burden of pneumonia hospitalizations in Hong Kong, 2011-2015. *European Journal of Clinical Microbiology & Infectious Diseases* 2019; **38**(3): 553-61.
37. Yu H, Huang J, Huai Y, et al. The substantial hospitalization burden of influenza in central China: surveillance for severe, acute respiratory infection, and influenza viruses, 2010-2012. *Influenza and other Respiratory Viruses* 2014; **8**(1): 53-65.
38. Shan W, Shi T, Zhang X, et al. Hospitalization Rate and Population-based Incidence of Hospitalization for Community-acquired Pneumonia Among Children in Suzhou, China. *Pediatric Infectious Disease Journal* 2018; **37**(12): 1242-7.
39. Wu PS, Chang IS, Tsai FY, et al. Epidemiology and impacts of children hospitalized with pneumonia from 1997 to 2004 in Taiwan. *Pediatric Pulmonology* 2009; **44**(2): 162-6.
40. Jensen-Fangel S, Mohey R, Johnsen SP, Andersen PL, Sorensen HT, Ostergaard L. Gender differences in hospitalization rates for respiratory tract infections in Danish youth. *Scandinavian Journal of Infectious Diseases* 2004; **36**(1): 31-6.
41. Sogaard M, Nielsen RB, Schonheyder HC, Norgaard M, Thomsen RW. Nationwide trends in pneumonia hospitalization rates and mortality, Denmark 1997-2011. *Respiratory Medicine* 2014; **108**(8): 1214-22.
42. Chicaiza-Ayala W, Henriquez-Trujillo AR, Ortiz-Prado E, Douce RW, Coral-Almeida M. The burden of acute respiratory infections in Ecuador 2011-2015. *PLoS ONE [Electronic Resource]* 2018; **13**(5): e0196650.
43. Janu EK, Annabattula BI, Kumariah S, et al. Paediatric hospitalisations for lower respiratory tract infections in Mount Isa. *Medical Journal of Australia* 2014; **200**(10): 591-4.
44. Rowlinson E, Dueger E, Taylor T, et al. Incidence and clinical features of respiratory syncytial virus infections in a population-based surveillance site in the Nile Delta Region. (Special Issue: Surveillance for respiratory syncytial virus in CDC's Global Disease Detection network: Epidemiology, disease burden and clinical characteristics.). *Journal of Infectious Diseases* 2013; **208**(Suppl. 3): S189-S96.
45. Casez P, Fauconnier J, Jorgensen L, et al. Longitudinal DRG-based survey of all-cause and pneumococcal pneumonia and meningitis for inpatients in France (2005-2010). *Medecine Et Maladies Infectieuses* 2015; **45**(11-12): 446-55.
46. Weigl JA, Puppe W, Schmitt HJ. Incidence of respiratory syncytial virus-positive hospitalizations in Germany. *European Journal of Clinical Microbiology & Infectious Diseases* 2001; **20**(7): 452-9.
47. Ntiri MP, Duque J, McMorrow ML, et al. Incidence of medically attended influenza among residents of Shai-Osudoku and Ningo-Prampram Districts, Ghana, May 2013 - April 2015. *BMC Infectious Diseases* 2016; **16**(1): 757.
48. Fathima P, Blyth CC, Lehmann D, et al. The Impact of Pneumococcal Vaccination on Bacterial and Viral Pneumonia in Western Australian Children: Record Linkage Cohort Study of 469589 Births, 1996-2012. *Clinical Infectious Diseases* 2018; **66**(7): 1075-85.

49. Verani JR, McCracken J, Arvelo W, et al. Surveillance for Hospitalized Acute Respiratory Infection in Guatemala. *Plos One* 2013; **8**(12).
50. Ludwig E, Jorgensen L, Gray S, Munson S, Chou K, Gutterman EM. [Clinical burden of multi-cause and pneumococcal pneumonia, meningitis, and septicemia in Hungary. Results of a retrospective study (2006-2011)]. *Orvosi Hetilap* 2014; **155**(36): 1426-36.
51. Hirve S, Krishnan A, Dawood FS, et al. Incidence of influenza-associated hospitalization in rural communities in western and northern India, 2010-2012: a multi-site population-based study. *Journal of Infection* 2015; **70**(2): 160-70.
52. Krishnan A, Kumar R, Broor S, et al. Epidemiology of viral acute lower respiratory infections in a community-based cohort of rural north Indian children. *Journal of Global Health* 2019; **9**(1): 010433.
53. Berezin EN, de Moraes JC, Hong T, Todd M, Seljan MP. Pneumonia hospitalization in Brazil from 2003 to 2007. *International Journal of Infectious Diseases* 2012; **16**(8): E583-E90.
54. Susilarini NK, Haryanto E, Praptiningsih CY, et al. Estimated incidence of influenza-associated severe acute respiratory infections in Indonesia, 2013-2016. *Influenza & Other Respiratory Viruses* 2018; **12**(1): 81-7.
55. Piroozi B, Alinia C, Moradi G, et al. Incidence, mortality, and burden of severe acute respiratory infection in Iran in 2015. *Iranian Journal of Public Health* 2019; **48**(Suppl. 1): 62-8.
56. Gouya M, Rezaei F, Haghdoost A, et al. Estimation of influenza and severe acute respiratory illness incidence (Burden) in three provinces of the Islamic Republic of Iran, 2012 and 2013. *Eastern Mediterranean Health Journal* 2016; **22**(7): 432-9.
57. Levy A, Fraser D, Vardi H, Dagan R. Hospitalizations for infectious diseases in Jewish and Bedouin children in southern Israel. *European Journal of Epidemiology* 1998; **14**(2): 179-86.
58. Baldo V, Cocchio S, Baldovin T, et al. A population-based study on the impact of hospitalization for pneumonia in different age groups. *BMC Infectious Diseases* 2014; **14**: 485.
59. Tornheim JA, Manya AS, Oyando N, Kabaka S, Breiman RF, Feikin DR. The epidemiology of hospitalized pneumonia in rural Kenya: the potential of surveillance data in setting public health priorities. *International Journal of Infectious Diseases* 2007; **11**(6): 536-43.
60. Feikin DR, Ope MO, Aura B, et al. The population-based burden of influenza-associated hospitalization in rural western Kenya, 2007-2009. *Bulletin of the World Health Organization* 2012; **90**(4): 256-63A.
61. Mohamed GA, Ahmed JA, Marano N, et al. Etiology and Incidence of Viral Acute Respiratory Infections Among Refugees Aged 5 Years and Older in Hagadera Camp, Dadaab, Kenya. *American Journal of Tropical Medicine & Hygiene* 2015; **93**(6): 1371-6.
62. Emukule GO, Khagayi S, McMorrow ML, et al. The burden of influenza and RSV among inpatients and outpatients in rural western Kenya, 2009-2012. *PLoS ONE [Electronic Resource]* 2014; **9**(8): e105543.
63. Dawa JA, Chaves SS, Nyawanda B, et al. National burden of hospitalized and non-hospitalized influenza-associated severe acute respiratory illness in Kenya, 2012-2014. *Influenza & Other Respiratory Viruses* 2018; **12**(1): 30-7.
64. Andrade AL, Afonso ET, Minamisava R, et al. Direct and indirect impact of 10-valent pneumococcal conjugate vaccine introduction on pneumonia hospitalizations and economic burden in all age-groups in Brazil: A time-series analysis. *PLoS ONE [Electronic Resource]* 2017; **12**(9): e0184204.

65. Silaba M, Ooko M, Bottomley C, et al. Effect of 10-valent pneumococcal conjugate vaccine on the incidence of radiologically-confirmed pneumonia and clinically-defined pneumonia in Kenyan children: an interrupted time-series analysis. *Lancet Global Health* 2019; **7**(3): e337-e46.
66. Peterson I, Bar-Zeev N, Kennedy N, et al. Respiratory virus-associated severe acute respiratory illness and viral clustering in Malawian children in a setting with a high prevalence of HIV infection, malaria, and malnutrition. *Journal of Infectious Diseases* 2016; **214**(11): 1700-11.
67. Burmaa A, Kamigaki T, Darmaa B, Nymadawa P, Oshitani H. Epidemiology and impact of influenza in Mongolia, 2007-2012. *Influenza and other Respiratory Viruses* 2014; **8**(5): 530-7.
68. van Gageldonk-Lafeber AB, Bogaerts MA, Verheij RA, van der Sande MA. Time trends in primary-care morbidity, hospitalization and mortality due to pneumonia. *Epidemiology & Infection* 2009; **137**(10): 1472-8.
69. Rozenbaum MH, Mangen MJ, Huijts SM, van der Werf TS, Postma MJ. Incidence, direct costs and duration of hospitalization of patients hospitalized with community acquired pneumonia: A nationwide retrospective claims database analysis. *Vaccine* 2015; **33**(28): 3193-9.
70. Grant CC, Scragg R, Tan D, Pati A, Aickin R, Yee RL. Hospitalisation for pneumonia in children in Auckland, New Zealand. *Journal of Paediatrics and Child Health* 1998; **34**(4): 355-9.
71. Chavez D, Gonzales-Armayo V, Mendoza E, et al. Estimation of influenza and respiratory syncytial virus hospitalizations using sentinel surveillance data-La Paz, Bolivia. 2012-2017. *Influenza & Other Respiratory Viruses* 2019; **13**(5): 477-83.
72. Becker-Dreps S, Blette B, Briceno R, et al. Changes in the incidence of pneumonia, bacterial meningitis, and infant mortality 5 years following introduction of the 13-valent pneumococcal conjugate vaccine in a "3+0" schedule. *PLoS ONE* 2017; **12**(8).
73. Munson S, Raluy-Callado M, Lambrelli D, Wasiak R, Eriksson D, Gray S. Clinical burden of pneumonia, meningitis and septicemia in Norway 2 years after 7-valent pneumococcal conjugate vaccine introduction. *Scandinavian Journal of Public Health* 2015; **43**(6): 657-66.
74. Abdel-Hady DM, Al-Balushi RM, Al-Abri BA, et al. Estimating the burden of influenza-associated hospitalization and deaths in Oman (2012-2015). (Special Issue: Influenza disease burden.). *Influenza and other Respiratory Viruses* 2018; **12**(1): 146-52.
75. Ieng V, Tolosa MX, Tek B, et al. National burden of influenza-associated hospitalizations in Cambodia, 2015 and 2016. (Special Issue: Centennial influenza pandemic.). *Western Pacific Surveillance and Response* 2018; **9**(Suppl. 1).
76. Myles PR, McKeever TM, Pogson Z, et al. The incidence of pneumonia using data from a computerized general practice database. *Epidemiology & Infection* 2009; **137**(5): 709-16.
77. Ashworth M, Charlton J, Latinovic R, Gulliford M. Age-related changes in consultations and antibiotic prescribing for acute respiratory infections, 1995-2000. Data from the UK General Practice Research Database. *Journal of Clinical Pharmacy and Therapeutics* 2006; **31**(5): 461-7.
78. Been JV, Szatkowski L, Staa TPv, et al. Smoke-free legislation and the incidence of paediatric respiratory infections and wheezing/asthma: interrupted time series analyses in the four UK nations. *Scientific Reports* 2015; **5**(1).
79. Kronman MP, Hersh AL, Feng R, Huang YS, Lee GE, Shah SS. Ambulatory visit rates and antibiotic prescribing for children with pneumonia, 1994-2007. *Pediatrics* 2011; **127**(3): 411-8.

80. Tong S, Amand C, Kieffer A, Kyaw MH. Trends in healthcare utilization and costs associated with pneumonia in the United States during 2008-2014. *BMC Health Services Research* 2018; **18**(1): 715.
81. Nelson JC, Jackson M, Yu O, et al. Impact of the introduction of pneumococcal conjugate vaccine on rates of community acquired pneumonia in children and adults. *Vaccine* 2008; **26**(38): 4947-54.
82. Van Deursen AMM, Verheij TJM, Rovers MM, et al. Trends in primary-care consultations, comorbidities, and antibiotic prescriptions for respiratory infections in The Netherlands before implementation of pneumococcal vaccines for infants. *Epidemiology and Infection* 2012; **140**(5): 823-34.
83. Cohen C, Walaza S, Moyes J, et al. Epidemiology of Severe Acute Respiratory Illness (SARI) among Adults and Children Aged >= 5 Years in a High HIV-Prevalence Setting, 2009-2012. *Plos One* 2015; **10**(2).
84. Monge V, González A. Hospital admissions for pneumonia in Spain. *Infection* 2001; **29**(1): 3-6.
85. Olsen SJ, Laosiritaworn Y, Siasiriwattana S, Chunsuttiwat S, Dowell SF. The incidence of pneumonia in rural Thailand. *International Journal of Infectious Diseases* 2006; **10**(6): 439-45.
86. Cummings MJ, Bakamutumaho B, Kayiwa J, et al. Epidemiologic and Spatiotemporal Characterization of Influenza and Severe Acute Respiratory Infection in Uganda, 2010-2015. *Annals of the American Thoracic Society* 2016; **13**(12): 2159-68.
87. Cromer D, van Hoek AJ, Jit M, Edmunds WJ, Fleming D, Miller E. The burden of influenza in England by age and clinical risk group: a statistical analysis to inform vaccine policy. *Journal of Infection* 2014; **68**(4): 363-71.
88. Iraola Ferro MI, Estevan Collazo MA, Bueno Pérez SM, et al. La neumonía del niño hospitalizado de cinco a catorce años de edad. *Arch pediatr Urug* 2005; **76**(3): 196-201.
89. Hui LIU, Xin-cai X, Jian-yun LU, Zong-qiu C, Lei LUO, Zhi-cong Y. Study on epidemic characteristics and etiology of community acquired pneumonia in Guangzhou from 2009 to 2012. *Chinese Journal of Preventive Medicine* 2013: 1089-94.
90. Kandeel A, Dawson P, Labib M, et al. Morbidity, Mortality, and Seasonality of Influenza Hospitalizations in Egypt, November 2007-November 2014. *PLoS ONE [Electronic Resource]* 2016; **11**(9): e0161301.
91. Macpherson L, Ogero M, Akech S, et al. Risk factors for death among children aged 5-14 years hospitalised with pneumonia: a retrospective cohort study in Kenya. *Bmj Global Health* 2019; **4**(5).
92. Nguenha N, Tivane A, Pale M, et al. Clinical and epidemiological characterization of influenza virus infections in children with severe acute respiratory infection in Maputo, Mozambique: results from the implementation of sentinel surveillance, 2014-2016. *PLoS ONE* 2018; **13**(3).
93. McMorrow ML, Wemakoy EO, Tshilobo JK, et al. Severe Acute Respiratory Illness Deaths in Sub-Saharan Africa and the Role of Influenza: A Case Series From 8 Countries. *Journal of Infectious Diseases* 2015; **212**(6): 853-60.
94. Ferreira S, Sant'anna CC, March Mde F, Santos MA, Cunha AJ. Lethality by pneumonia and factors associated to death. *Jornal de Pediatria* 2014; **90**(1): 92-7.

95. Araya S, Lovera D, Zarate C, et al. Application of a Prognostic Scale to Estimate the Mortality of Children Hospitalized with Community-acquired Pneumonia. *Pediatric Infectious Disease Journal* 2016; **35**(4): 369-73.
96. Schanzer DL, McGeer A, Morris K. Statistical estimates of respiratory admissions attributable to seasonal and pandemic influenza for Canada. *Influenza and other Respiratory Viruses* 2013; **7**(5): 799-808.
97. Chong CY, Yung CF, Gan C, et al. The burden and clinical manifestation of hospitalized influenza among different pediatric age-groups in the tropics. *Influenza and Other Respiratory Viruses* 2020; **14**(1): 46-54.
98. Schanzer DL, Saboui M, Lee L, Nwosu A, Bancej C. Burden of influenza, respiratory syncytial virus, and other respiratory viruses and the completeness of respiratory viral identification among respiratory inpatients, Canada, 2003-2014. (Special Issue: Influenza disease burden.). *Influenza and other Respiratory Viruses* 2018; **12**(1): 113-21.
99. Oliva J, Delgado-Sanz C, Larrauri A, Spanish Influenza Surveillance S. Estimating the burden of seasonal influenza in Spain from surveillance of mild and severe influenza disease, 2010-2016. *Influenza & Other Respiratory Viruses* 2018; **12**(1): 161-70.
100. Meury S, Zeller S, Heininger U. Comparison of clinical characteristics of influenza and respiratory syncytial virus infection in hospitalised children and adolescents. *European Journal of Pediatrics* 2004; **163**(7): 359-63.
101. Simmerman JM, Chittaganpitch M, Levy J, et al. Incidence, seasonality and mortality associated with influenza pneumonia in Thailand: 2005-2008. *PLoS ONE [Electronic Resource]* 2009; **4**(11): e7776.
102. Boddington NL, Verlander NQ, Pebody RG. Developing a system to estimate the severity of influenza infection in England: findings from a hospital-based surveillance system between 2010/2011 and 2014/2015. *Epidemiology & Infection* 2017; **145**(7): 1461-70.
103. Proff R, Gershman K, Lezotte D, Nyquist AC. Case-based surveillance of influenza hospitalizations during 2004-2008, Colorado, USA. *Emerging Infectious Diseases* 2009; **15**(6): 892-8.
104. Jain S, Williams DJ, Arnold SR, et al. Community-acquired pneumonia requiring hospitalization among U.S. children. *New England Journal of Medicine* 2015; **372**(9): 835-45.
105. Henrickson KJ, Hoover S, Kehl KS, Hua W. National disease burden of respiratory viruses detected in children by polymerase chain reaction. *Pediatric Infectious Disease Journal* 2004; **23**(1 Suppl): S11-8.
106. Coffin SE, Zaoutis TE, Rosenquist AB, et al. Incidence, complications, and risk factors for prolonged stay in children hospitalized with community-acquired influenza. *Pediatrics* 2007; **119**(4): 740-8.
107. Ampofo K, Gesteland PH, Bender J, et al. Epidemiology, complications, and cost of hospitalization in children with laboratory-confirmed influenza infection. *Pediatrics* 2006; **118**(6): 2409-17.
108. Dawood FS, Fiore A, Kamimoto L, et al. Burden of seasonal influenza hospitalization in children, United States, 2003 to 2008. *Journal of Pediatrics* 2010; **157**(5): 808-14.
109. Kostova D, Reed C, Finelli L, et al. Influenza Illness and Hospitalizations Averted by Influenza Vaccination in the United States, 2005-2011. *PLoS ONE [Electronic Resource]* 2013; **8**(6): e66312.

110. Centers for Disease C, Prevention. Estimated influenza illnesses and hospitalizations averted by influenza vaccination - United States, 2012-13 influenza season. *MMWR - Morbidity & Mortality Weekly Report* 2013; **62**(49): 997-1000.
111. Reed C, Kim IK, Singleton JA, et al. Estimated influenza illnesses and hospitalizations averted by vaccination--United States, 2013-14 influenza season. *MMWR - Morbidity & Mortality Weekly Report* 2014; **63**(49): 1151-4.
112. Rolfes MA, Flannery B, Chung JR, et al. Effects of influenza vaccination in the United States during the 2017-2018 influenza season. *Clinical Infectious Diseases* 2019; **69**(11): 1845-53.
113. Xu X, Blanton L, Elal AIA, et al. Update: Influenza Activity in the United States During the 2018-19 Season and Composition of the 2019-20 Influenza Vaccine. *MMWR - Morbidity & Mortality Weekly Report* 2019; **68**(24): 544-51.
114. Hughes MM, Carmack AE, McCaffrey K, et al. Estimating the Incidence of Influenza at the State Level - Utah, 2016-17 and 2017-18 Influenza Seasons. *Mmwr* 2019; **Morbidity and mortality weekly report. 68**(50): 1158-61.
115. Yoshihara K, Le MN, Toizumi M, et al. Influenza B associated paediatric acute respiratory infection hospitalization in central vietnam. *Influenza and Other Respiratory Viruses* 2019; **13**(3): 248-61.
116. Chiu SS, Chan KH, Chen H, et al. Virologically confirmed population-based burden of hospitalization caused by respiratory syncytial virus, adenovirus, and parainfluenza viruses in children in Hong Kong. *Pediatric Infectious Disease Journal* 2010; **29**(12): 1088-92.
117. Nelson EAS, Ip M, Tam JS, et al. Burden of influenza infection in hospitalised children below 6 months of age and above in Hong Kong from 2005 to 2011. *Vaccine* 2014; **32**(49): 6692-8.
118. Li-Kim-Moy J, Yin JK, Patel C, et al. Australian vaccine preventable disease epidemiological review series: influenza 2006 to 2015. *Communicable Diseases Intelligence* 2016; **40**(4): E482-E95.
119. Silvennoinen H, Peltola V, Vainionpaa R, Ruuskanen O, Heikkinen T. Incidence of influenza-related hospitalizations in different age groups of children in Finland: a 16-year study. *Pediatric Infectious Disease Journal* 2011; **30**(2): e24-8.
120. von der Beck D, Seeger W, Herold S, Gunther A, Loh B. Characteristics and outcomes of a cohort hospitalized for pandemic and seasonal influenza in Germany based on nationwide inpatient data. *PLoS ONE [Electronic Resource]* 2017; **12**(7): e0180920.
121. Weigl JAI, Puppe W, Schmitt HJ. The incidence of influenza-associated hospitalizations in children in Germany. *Epidemiology & Infection* 2002; **129**(3): 525-33.
122. Sakkou Z, Stripeli F, Papadopoulos NG, et al. Impact of influenza infection on children's hospital admissions during two seasons in Athens, Greece. *Vaccine* 2011; **29**(6): 1167-72.
123. Rebolledo J, Igoe D, O'Donnell J, et al. Influenza in hospitalized children in Ireland in the pandemic period and the 2010/2011 season: risk factors for paediatric intensive-care-unit admission. *Epidemiology & Infection* 2014; **142**(9): 1826-35.
124. Onyango CO, Njeru R, Kazungu S, et al. Influenza surveillance among children with pneumonia admitted to a district hospital in coastal Kenya, 2007-2010. *Journal of Infectious Diseases* 2012; **206 Suppl 1**: S61-7.

BMJ Publishing Group Limited (BMJ) disclaims all liability and responsibility arising from any reliance placed on this supplemental material which has been supplied by the author(s)


125. Al-Awaidy S, Hamid S, Al-Obaidani I, et al. The burden of influenza-associated hospitalizations in Oman, January 2008-June 2013. *PLoS ONE* 2015; **10**(12).
126. Tinoco YO, Azziz-Baumgartner E, Uyeki TM, et al. Burden of Influenza in 4 Ecologically Distinct Regions of Peru: Household Active Surveillance of a Community Cohort, 2009-2015. *Clinical Infectious Diseases* 2017; **65**(9): 1532-41.
127. Tallo VL, Kamigaki T, Tan AG, et al. Estimating influenza outpatients' and inpatients' incidences from 2009 to 2011 in a tropical urban setting in the Philippines. *Influenza & Other Respiratory Viruses* 2014; **8**(2): 159-68.
128. Kenmoe S, Tchendjou P, Vernet MA, et al. Viral etiology of severe acute respiratory infections in hospitalized children in Cameroon, 2011-2013. *Influenza & Other Respiratory Viruses* 2016; **10**(5): 386-93.
129. Bukhari EE, Elhazmi MM. Viral agents causing acute lower respiratory tract infections in hospitalized children at a tertiary care center in Saudi Arabia. *Saudi Medical Journal* 2013; **34**(11): 1151-5.
130. Seung-Jin LEE, Shin-Hae LEE, Eun-Kyo HA, et al. Prevalence of respiratory virus infection with regard to age, sex, and seasonality factors: A single center experience against children hospitalized during the 10 years. *Allergy, Asthma & Respiratory Disease* 2017: 320-5.
131. Kang-Mo AHN, So-Hee C, Eun-Hee C, et al. Clinical characteristics of acute viral lower respiratory tract infections in hospitalized children in Seoul, 1996-1998. *Journal of Korean Medical Science* 1999: 405-11.
132. Gwi-Ok P, Ji-Hyun KIM, Jae-Hee LEE, et al. Epidemiologic and clinical features in children with acute lower respiratory tract infection caused by human metapneumovirus in 2006-2007. *Korean Journal of Pediatrics* 2009: 330-8.
133. Cebey-Lopez M, Herberg J, Pardo-Seco J, et al. Viral Co-Infections in Pediatric Patients Hospitalized with Lower Tract Acute Respiratory Infections. *PLoS ONE [Electronic Resource]* 2015; **10**(9): e0136526.
134. Harun A, Beyza E. Viral and Atypical Bacterial Respiratory Infections in a University Teaching Hospital. *Japanese Journal of Infectious Diseases* 2019; **72**(5): 318-22.
135. Bicer S, Giray T, Col D, et al. Virological and clinical characterizations of respiratory infections in hospitalized children. *Italian Journal of Pediatrics* 2013; **39**: 22.
136. Jain S, Self WH, Wunderink RG, et al. Community-Acquired Pneumonia Requiring Hospitalization among U.S. Adults. *New England Journal of Medicine* 2015; **373**(5): 415-27.
137. Anh Ha Lien D, Doorn HRv, My Ngoc N, et al. Viral etiologies of acute respiratory infections among hospitalized Vietnamese children in Ho Chi Minh City, 2004-2008. *PLoS ONE* 2011; **38**.
138. Nguyen HKL, Nguyen SV, Nguyen AP, et al. Surveillance of Severe Acute Respiratory Infection (SARI) for Hospitalized Patients in Northern Vietnam, 2011-2014. *Japanese Journal of Infectious Diseases* 2017; **70**(5): 522-7.
139. Hatem A, Mohamed S, Abu Elhassan UE, et al. Clinical characteristics and outcomes of patients with severe acute respiratory infections (SARI): results from the Egyptian surveillance study 2010-2014. *Multidiscip Respir Med* 2019; **14**: 11.
140. Zhang Q, Guo Z, MacDonald NE. Vaccine preventable community-acquired pneumonia in hospitalized children in Northwest China. *Pediatr Infect Dis J* 2011; **30**(1): 7-10.

141. 张蕾. 儿童下呼吸道感染的病毒病原检测分析 [硕士]: 西南医科大学;泸州医学院; 2008.
142. 谢红军, 李征. 小儿急性呼吸道感染 3309 例病毒抗原检测及分析. *湖南师范大学学报（医学版）* 2017; **14**(1): 52-5.
143. Yu J, Xie Z, Zhang T, et al. Comparison of the prevalence of respiratory viruses in patients with acute respiratory infections at different hospital settings in North China, 2012-2015. *BMC Infectious Diseases* 2018; **18**(1): 72.
144. Zheng-de XIE, Yan X, Chun-yan LIU, et al. Three years surveillance of viral etiology of acute lower respiratory tract infection in children from 2007 to 2010. *Chinese Journal of Pediatrics* 2011: 745-9.
145. Zhang TG, Li AH, Lyu M, Chen M, Huang F, Wu J. Detection of respiratory viral and bacterial pathogens causing pediatric community-acquired pneumonia in Beijing using real-time PCR. *Chronic Diseases and Translational Medicine* 2015; **1**(2): 110-6.
146. Ni-Guang X, Bing Z, Zhao-Jun D, et al. Viral etiology of 1165 hospitalized children with acute lower respiratory tract infection. *Chinese Journal of Contemporary Pediatrics* 2012: 28-32.
147. Quan-Heng LI, Wen-Jie GAO, Jin-Ying LI, et al. Detection of respiratory viruses in children with acute lower respiratory tract infection: an analysis of 5,150 children. *Chinese Journal of Contemporary Pediatrics* 2016: 51-4.
148. Cowling BJ, Chan KH, Feng S, et al. The effectiveness of influenza vaccination in preventing hospitalizations in children in Hong Kong, 2009-2013. *Vaccine* 2014; **32**(41): 5278-84.
149. Jin Y, Zhang RF, Xie ZP, et al. Newly identified respiratory viruses associated with acute lower respiratory tract infections in children in Lanzou, China, from 2006 to 2009. *Clinical Microbiology and Infection* 2012; **18**(1): 74-80.
150. Feng L, Li Z, Zhao S, et al. Viral etiologies of hospitalized acute lower respiratory infection patients in China, 2009-2013. *PLoS ONE* 2014; **9**(6).
151. Mei Z, Xiaohong W, Hui YU, Qirong ZHU. Epidemiological characteristics of common respiratory viruses among children with acute respiratory tract infections in Shanghai. *Chinese Journal of Infectious Diseases* 2008: 527-32.
152. Liu P, Xu M, He L, et al. Epidemiology of Respiratory Pathogens in Children with Lower Respiratory Tract Infections in Shanghai, China, from 2013 to 2015. *Jpn J Infect Dis* 2018; **71**(1): 39-44.
153. Li J, Tao Y, Tang M, et al. Rapid detection of respiratory organisms with the FilmArray respiratory panel in a large children's hospital in China. *BMC Infectious Diseases* 2018; **18**(510).
154. Cai XY, Wang Q, Lin GY, et al. Respiratory virus infections among children in South China. *Journal of Medical Virology* 2014; **86**(7): 1249-55.
155. Lie H, Fansheng Z, Qiong W, Runxiang WU, Xiaofang LUO, Xuedong LU. Detection for respiratory viruses in children with multiplex PCR. *Chinese Journal of Microbiology and Immunology* 2009: 664-7.
156. Oumei H, Xuefeng W, Jianping L, et al. Etiology of community-acquired pneumonia in 1500 hospitalized children. *Journal of Medical Virology* 2018; **90**(3): 421-8.
157. Wang H, Zheng Y, Deng J, et al. Prevalence of respiratory viruses among children hospitalized from respiratory infections in Shenzhen, China. *Virology Journal* 2016; **13**: 39.

158. Xu W, Guo L, Dong X, et al. Detection of Viruses and Mycoplasma pneumoniae in Hospitalized Patients with Severe Acute Respiratory Infection in Northern China, 2015-2016. *Japanese Journal of Infectious Diseases* 2018; **71**(2): 134-9.
159. Cheng W, Yu Z, Liu S, et al. Comparison of Influenza Epidemiological and Virological Characteristics between Outpatients and Inpatients in Zhejiang Province, China, March 2011-June 2015. *International Journal of Environmental Research & Public Health [Electronic Resource]* 2017; **14**(2): 22.
160. Kavunga-Membo H, Nkwembe E, Simulundu E, et al. Epidemiology of circulating human influenza viruses from the democratic republic of congo, 2015. *PLoS ONE* 2018; **13 (9) (no pagination)**(e0203995).
161. Refaey S, Hassan M, Mansour A, Kandeel A. Incidence of influenza virus-associated severe acute respiratory infection in Damanhour district, Egypt, 2013. *Eastern Mediterranean Health Journal* 2016; **22**(7): 503-12.
162. Refaey S, Amin M, Labib M, Kandeel A. Influenza virus positivity and circulating subtypes among cases of influenza-like illness and severe acute respiratory infection, Egypt, 2012-2015. *Eastern Mediterranean Health Journal* 2016; **22**(7): 527-36.
163. Moore HC, de Klerk N, Keil AD, et al. Use of data linkage to investigate the aetiology of acute lower respiratory infection hospitalisations in children. *Journal of Paediatrics & Child Health* 2012; **48**(6): 520-8.
164. Weigl JA, Puppe W, Grondahl B, Schmitt HJ. Epidemiological investigation of nine respiratory pathogens in hospitalized children in Germany using multiplex reverse-transcriptase polymerase chain reaction. *European Journal of Clinical Microbiology & Infectious Diseases* 2000; **19**(5): 336-43.
165. Tsolia MN, Psarras S, Bossios A, et al. Etiology of community-acquired pneumonia in hospitalized school-age children: evidence for high prevalence of viral infections. *Clinical Infectious Diseases* 2004; **39**(5): 681-6.
166. Panda S, Mohakud NK, Suar M, Kumar S. Etiology, seasonality, and clinical characteristics of respiratory viruses in children with respiratory tract infections in Eastern India (Bhubaneswar, Odisha). *Journal of Medical Virology* 2017; **89**(3): 553-8.
167. Storms AD, Kusriastuti R, Misriyah S, et al. The East Jakarta Project: surveillance for highly pathogenic avian influenza A(H5N1) and seasonal influenza viruses in patients seeking care for respiratory disease, Jakarta, Indonesia, October 2011-September 2012. *Epidemiology & Infection* 2015; **143**(16): 3394-404.
168. Lafond KE, Praptiningsih CY, Mangiri A, et al. Seasonal influenza and avian influenza A(H5N1) virus surveillance among inpatients and outpatients, East Jakarta, Indonesia, 2011-2014. *Emerging Infectious Diseases* 2019; **25**(11): 2031-9.
169. Botti C, Micillo A, Ricci G, et al. Characterization of respiratory infection viruses in hospitalized children from Naples province in Southern Italy. *Experimental and Therapeutic Medicine* 2018; **15**(6): 4805-9.
170. Al-Abdallat M, Dawson P, Haddadin AJ, et al. Influenza hospitalization epidemiology from a severe acute respiratory infection surveillance system in Jordan, January 2008-February 2014. *Influenza & Other Respiratory Viruses* 2016; **10**(2): 91-7.

171. Saleh M, Bazzi L, Ismail E, et al. Influenza-associated severe acute respiratory infections in 2 sentinel sites in Lebanon-September 2015 to August 2016. *Influenza & Other Respiratory Viruses* 2018; **12**(3): 331-5.
172. Barakat A, Ihazmad H, Benkaroum S, et al. Influenza surveillance among outpatients and inpatients in Morocco, 1996-2009. *PLoS ONE [Electronic Resource]* 2011; **6**(9): e24579.
173. Talla Nzussouo N, Duque J, Adedeji AA, et al. Epidemiology of influenza in West Africa after the 2009 influenza A(H1N1) pandemic, 2010-2012. *BMC Infectious Diseases* 2017; **17 (1) (no pagination)**(745).
174. Radin JM, Katz MA, Tempia S, et al. Influenza surveillance in 15 countries in Africa, 2006-2010. *Journal of Infectious Diseases* 2012; **206 Suppl 1**: S14-21.
175. Wertheim HFL, Nadjm B, Thomas S, et al. Viral and atypical bacterial aetiologies of infection in hospitalised patients admitted with clinical suspicion of influenza in Thailand, Vietnam and Indonesia. *Influenza and other Respiratory Viruses* 2015; **9**(6): 315-22.
176. Berg AS, Inchley CS, Aase A, et al. Etiology of Pneumonia in a Pediatric Population with High Pneumococcal Vaccine Coverage A Prospective Study. *Pediatric Infectious Disease Journal* 2016; **35**(3): E69-E75.
177. Abdel-Hady DM, Al Balushi RM, Al Abri BA, et al. Estimating the burden of influenza-associated hospitalization and deaths in Oman (2012-2015). *Influenza and other Respiratory Viruses* 2018; **12**(1): 146-52.
178. Goldstein E, Nguyen HH, Liu P, et al. On the Relative Role of Different Age Groups during Epidemics Associated with Respiratory Syncytial Virus. *Journal of Infectious Diseases* 2018; **217**(2): 238-44.
179. Saravanos GL, Sheel M, Homaira N, et al. Respiratory syncytial virus-associated hospitalisations in Australia, 2006-2015. *Medical Journal of Australia* 2019; **210**(10): 447-53.
180. McCracken JP, Prill MM, Arvelo W, et al. Respiratory Syncytial Virus Infection in Guatemala, 2007-2012. *Journal of Infectious Diseases* 2013; **208**: S197-S206.
181. Moe N, Stenseng IH, Krokstad S, et al. The Burden of Human Metapneumovirus and Respiratory Syncytial Virus Infections in Hospitalized Norwegian Children. *Journal of Infectious Diseases* 2017; **216**(1): 110-6.
182. Lovato-Salas F, Matienzo-Serment L, Monjaras-Avila C, et al. Pandemic influenza A(H1N1) 2009 and respiratory syncytial virus associated hospitalizations. *Journal of Infection* 2010; **61**(5): 382-90.
183. 黄育涛, 蔡幸生, 朱勇斌. 急性下呼吸道感染住院患儿病毒病原学分析. *广州医药* 2018; (03): 62-4.
184. Chen X, Zhang ZY, Zhao Y, Liu EM, Zhao XD. Acute lower respiratory tract infections by human metapneumovirus in children in Southwest China: a 2-year study. *Pediatric Pulmonology* 2010; **45**(8): 824-31.
185. Jain B, Singh AK, Dangi T, et al. High prevalence of human metapneumovirus subtype B in cases presenting as severe acute respiratory illness: an experience at tertiary care hospital. *The clinical respiratory journal* 2014; **8**(2): 225-33.
186. Bigogo GM, Breiman RF, Feikin DR, et al. Epidemiology of respiratory syncytial virus infection in rural and urban Kenya. *Journal of Infectious Diseases* 2013; **208 Suppl 3**: S207-16.

187. Zhang L, Liu W, Liu D, et al. Epidemiological and clinical features of human metapneumovirus in hospitalised paediatric patients with acute respiratory illness: a cross-sectional study in Southern China, from 2013 to 2016. *BMJ Open* 2018; **8**(2): e019308.
188. Ji W, Wang Y, Chen Z, Shao X, Ji Z, Xu J. Human metapneumovirus in children with acute respiratory tract infections in Suzhou, China 2005-2006. *Scandinavian Journal of Infectious Diseases* 2009; **41**(10): 735-44.
189. Wang Y, Chen Z, Yan YD, et al. Seasonal distribution and epidemiological characteristics of human metapneumovirus infections in pediatric inpatients in Southeast China. *Archives of Virology* 2013; **158**(2): 417-24.
190. Pan Y, Zhang Y, Shi W, et al. Human parainfluenza virus infection in severe acute respiratory infection cases in Beijing, 2014-2016: A molecular epidemiological study. *Influenza & Other Respiratory Viruses* 2017; **11**(6): 564-8.
191. Chan PK, Tam WW, Lee TC, et al. Hospitalization Incidence, Mortality, and Seasonality of Common Respiratory Viruses Over a Period of 15 Years in a Developed Subtropical City. *Medicine* 2015; **94**(46): e2024.
192. Davis CR, Stockmann C, Pavia AT, et al. Incidence, Morbidity, and Costs of Human Metapneumovirus Infection in Hospitalized Children. *Journal of the Pediatric Infectious Diseases Societ* 2016; **5**(3): 303-11.
193. McCracken JP, Arvelo W, Ortiz J, et al. Comparative epidemiology of human metapneumovirus- and respiratory syncytial virus-associated hospitalizations in Guatemala. *Influenza and Other Respiratory Viruses* 2014; **8**(4): 414-21.
194. Morgan OW, Chittaganpitch M, Clague B, et al. Hospitalization due to human parainfluenza virus-associated lower respiratory tract illness in rural Thailand. *Influenza and other Respiratory Viruses* 2013; **7**(3): 280-5.
195. Anonymous. The epidemiology of influenza in children hospitalized in Canada, 2004-2005, in Immunization Monitoring Program Active (IMPACT) centres. *Canada Communicable Disease Report = Releve des Maladies Transmissibles au Canada* 2006; **32**(7): 77-86.
196. Torner N, Martinez A, Basile L, et al. Descriptive study of severe hospitalized cases of laboratory-confirmed influenza during five epidemic seasons (2010-2015). *BMC research notes* 2018; **11**(1): 244.
197. San-Román-Montero JM, Gil Prieto R, Gallardo Pino C, Hinojosa Mena J, Zapatero Gaviria A, Gil de Miguel A. Inpatient hospital fatality related to coding (ICD-9-CM) of the influenza diagnosis in Spain (2009-2015). *BMC Infectious Diseases* 2019; **19**(1): N.PAG-N.PAG.
198. Moore DL, Vaudry W, Scheifele DW, et al. Surveillance for influenza admissions among children hospitalized in Canadian immunization monitoring program active centers, 2003-2004. *Pediatrics* 2006; **118**(3): e610-9.
199. Blyth CC, Macartney KK, Hewagama S, et al. Influenza epidemiology, vaccine coverage and vaccine effectiveness in children admitted to sentinel Australian hospitals in 2014: the Influenza Complications Alert Network (FluCAN). *Euro Surveillance: Bulletin Europeen sur les Maladies Transmissibles = European Communicable Disease Bulletin* 2016; **21**(30): 28.
200. Xu CL, Iuliano AD, Chen M, et al. Characteristics of Hospitalized Cases with Influenza A (H1N1) pdm09 Infection during First Winter Season of Post-Pandemic in China. *Plos One* 2013; **8**(2).

201. Li-Kim-Moy J, Yin JK, Blyth CC, et al. Influenza hospitalizations in Australian children. *Epidemiology & Infection* 2017; **145**(7): 1451-60.
202. Sahu M, Singh N, Shukla MK, et al. Molecular and epidemiological analysis of pandemic and post-pandemic influenza A(H1N1)pdm09 virus from central India. *Journal of Medical Virology* 2018; **90**(3): 447-55.
203. Davila-Torres J, Chowell G, Borja-Aburto VH, Viboud C, Grajalez-Muniz C, Miller MA. Intense seasonal A/H1N1 influenza in Mexico, winter 2013-2014. *Archives of Medical Research* 2015; **46**(1): 63-70.
204. Borja-Aburto VH, Chowell G, Viboud C, et al. Epidemiological characterization of a fourth wave of pandemic A/H1N1 influenza in Mexico, winter 2011-2012: age shift and severity. *Archives of Medical Research* 2012; **43**(7): 563-70.
205. Meerhoff TJ, Simaku A, Ulqinaku D, et al. Surveillance for severe acute respiratory infections (SARI) in hospitals in the WHO European region - an exploratory analysis of risk factors for a severe outcome in influenza-positive SARI cases. *BMC Infectious Diseases* 2015; **15**: 1.
206. Puig-Barbera J, Natividad-Sancho A, Trushakova S, et al. Epidemiology of Hospital Admissions with Influenza during the 2013/2014 Northern Hemisphere Influenza Season: Results from the Global Influenza Hospital Surveillance Network. *PLoS ONE [Electronic Resource]* 2016; **11**(5): e0154970.
207. Rodriguez-Auad JP, Nava-Frias M, Casasola-Flores J, et al. The epidemiology and clinical characteristics of respiratory syncytial virus infection in children at a public pediatric referral hospital in Mexico. *International Journal of Infectious Diseases* 2012; **16**(7): e508-e13.
208. Ng KF, Tan KK, Sam ZH, Ting GSS, Gan WY. Epidemiology, clinical characteristics, laboratory findings and severity of respiratory syncytial virus acute lower respiratory infection in Malaysian children, 2008-2013. *Journal of Paediatrics and Child Health* 2017; **53**(4): 399-407.
209. Hahn A, Wang W, Jaggi P, et al. Human metapneumovirus infections are associated with severe morbidity in hospitalized children of all ages. *Epidemiology & Infection* 2013; **141**(10): 2213-23.
210. Laurichesse H, Dedman D, Watson JM, Zambon MC. Epidemiological features of parainfluenza virus infections: laboratory surveillance in England and Wales, 1975-1997. *European Journal of Epidemiology* 1999; **15**(5): 475-84.
